# Integrative Analysis of DNA Methylation and Gene Expression Data Identifies *EPAS1* as a Key Regulator of COPD

**DOI:** 10.1371/journal.pgen.1004898

**Published:** 2015-01-08

**Authors:** Seungyeul Yoo, Sachiko Takikawa, Patrick Geraghty, Carmen Argmann, Joshua Campbell, Luan Lin, Tao Huang, Zhidong Tu, Robert Feronjy, Avrum Spira, Eric E. Schadt, Charles A. Powell, Jun Zhu

**Affiliations:** Institute of Genomics and Multiscale Biology, Mount Sinai School of Medicine, New York, New York, United States of America; Department of Genetics and Genomic Sciences, Mount Sinai School of Medicine, New York, New York, United States of America; Division of Pulmonary, Critical Care and Sleep Medicine, Mount Sinai School of Medicine, New York, New York, United States of America; Department of Medicine, St. Luke's Roosevelt Medical Center, Mount Sinai School of Medicine, New York, New York, United States of America; Division of Computational Biomedicine, Department of Medicine, Boston University School of Medicine, Boston, Massachusetts, United States of America; Georgia Institute of Technology, United States of America

## Abstract

Chronic Obstructive Pulmonary Disease (COPD) is a complex disease. Genetic, epigenetic, and environmental factors are known to contribute to COPD risk and disease progression. Therefore we developed a systematic approach to identify key regulators of COPD that integrates genome-wide DNA methylation, gene expression, and phenotype data in lung tissue from COPD and control samples. Our integrative analysis identified 126 key regulators of COPD. We identified *EPAS1* as the only key regulator whose downstream genes significantly overlapped with multiple genes sets associated with COPD disease severity. *EPAS1* is distinct in comparison with other key regulators in terms of methylation profile and downstream target genes. Genes predicted to be regulated by *EPAS1* were enriched for biological processes including signaling, cell communications, and system development. We confirmed that EPAS1 protein levels are lower in human COPD lung tissue compared to non-disease controls and that *Epas1* gene expression is reduced in mice chronically exposed to cigarette smoke. As *EPAS1* downstream genes were significantly enriched for hypoxia responsive genes in endothelial cells, we tested *EPAS1* function in human endothelial cells. *EPAS1* knockdown by siRNA in endothelial cells impacted genes that significantly overlapped with *EPAS1* downstream genes in lung tissue including hypoxia responsive genes, and genes associated with emphysema severity. Our first integrative analysis of genome-wide DNA methylation and gene expression profiles illustrates that not only does DNA methylation play a ‘causal’ role in the molecular pathophysiology of COPD, but it can be leveraged to directly identify novel key mediators of this pathophysiology.

## Author Summary

Chronic Obstructive Pulmonary Disease (COPD) is a common lung disease. It is the fourth leading cause of death in the world and is expected to be the third by 2020. COPD is a heterogeneous and complex disease consisting of obstruction in the small airways, emphysema, and chronic bronchitis. COPD is generally caused by exposure to noxious particles or gases, most commonly from cigarette smoking. However, only 20–25% of smokers develop clinically significant airflow obstruction. Smoking is known to cause epigenetic changes in lung tissues. Thus, genetics, epigenetic, and their interaction with environmental factors play an important role in COPD pathogenesis and progression. Currently, there are no therapeutics that can reverse COPD progression. In order to identify new targets that may lead to the development of therapeutics for curing COPD, we developed a systematic approach to identify key regulators of COPD that integrates genome-wide DNA methylation, gene expression, and phenotype data in lung tissue from COPD and control samples. Our integrative analysis identified 126 key regulators of COPD. We identified *EPAS1* as the only key regulator whose downstream genes significantly overlapped with multiple genes sets associated with COPD disease severity.

## Introduction

Chronic Obstructive Pulmonary Disease (COPD) is a common lung disease. It is the fourth leading cause of death in the world and is expected to be the third by 2020 [1]. COPD is a heterogeneous and complex disease consisting of obstruction in the small airways, emphysema, and chronic bronchitis [2]. Patients with COPD generally have an increased level of systemic inflammation and progressive loss of lung function by irreversible airflow limitations [3]. COPD is generally caused by exposure to noxious particles or gases, most commonly from cigarette smoking [4]–[6]. However, only 20–25% of smokers develop clinically significant airflow obstruction [7], which suggests that inter-individual differences related in part to genetic susceptibility play an important role in modifying the risk of disease in individuals [8].

Genome-wide association studies (GWAS) have recently identified several risk loci for COPD and/or smoking associated genes [4], [9]–[13]. While these studies have provided an initial look into the genetic architecture of COPD, they have been limited by size, by heterogeneity of disease phenotype, and by potential confounders relating to the amount of cigarette smoking. The smaller genetic variance component for COPD identified to date could be due to environmental factors and/or epigenetic regulation. Indeed, epigenetic changes are a factor in many diseases, including many different types of cancer [14]. In the lung DNA methylation is an important factor for normal lung function [15], and several studies have recently confirmed that DNA methylation is significantly associated with lung cancer [16]–[18]. Moreover, smoking, which is one of the major risk factors of COPD, is considered as one of important modifiers of DNA methylation [19], [20] and it is also known to cause epigenetic changes in lung tissue [21], [22]. Therefore, understanding the transcriptional regulation by epigenetic factors such as DNA methylation may shed light on understanding the biological processes associated with COPD susceptibility, severity, and COPD comorbidities such as lung cancer. Recently, DNA methylation is shown to be associated with COPD and lung function, suggesting that genetic and epigenetic pathways may contribute to COPD [23], [24]. While previous studies have provided potentially important CpG loci associated with COPD, they have not yet clarified the role variations in methylation play in regulating global gene expression and the biological consequences of such regulation.

In this study we present a novel systematic approach for identifying key regulators in COPD by integrating functional genomic, epigenetic data, and higher order phenotypic data. We studied 100 COPD and 52 control (CTRL) lung samples to investigate the relationship between methylation status of DNA and expression level of a gene either in close (*cis*) or far (*trans*) proximity to the methylated site. The primary focus of this study is not only to identify those regions that are differentially methylated between COPD cases and controls, but to resolve the gene expression changes that follow as a result of these differentially methylated regions and the biological consequences that these regulatory changes induce with respect to disease development or progression. Our integrative analysis of DNA methylation and gene expression validates the importance of DNA methylation in COPD and enables the direct identification of novel key regulators modulated by epigenetic changes in this multifactorial, systematic disease. When comparing downstream genes controlled by the key regulators with gene sets related to COPD disease severity, we identified *EPAS1* as the only key regulator whose downstream genes significantly overlapped with multiple gene sets related to COPD. We further show that EPAS1 protein levels are lower in lung tissues of COPD patients. *Epas1* is down-regulated transcriptionally by chronic smoke exposure in mice, and the *EPAS1* knockdown signature in human endothelial cells significantly overlaps with our predicted *EPAS1* downstream genes. These data combined suggest that our systematic approach can provide important insights into understanding the mechanisms underlying epigenetic regulation, via DNA methylation, that in turn alters transcriptional programs that lead to COPD pathogenesis and progression.

## Results

Both genome-wide DNA methylation and gene expression profiles of 62 non-COPD controls (CTRL) and 148 COPD lung samples were obtained from the collaborative project, Lung Genomics Research Consortium (LGRC). To carry out an integrative analysis, we required that methylation and gene expression data were properly aligned. Therefore, we developed a multi-omic data alignment procedure to iteratively match methylation and gene expression profiles within each individual in the study [25]. Samples that could not be ambiguously matched were filtered out, leaving a final dataset for analysis consisting of 52 CTRL and 100 COPD sample pairs. Demographic characteristics of these samples are listed in S1 Table. After quality checks of methylation intensity, 1.7 M and 1.78 M methyl probes for CTRL and COPD samples, respectively, were retained for further analysis (S2 Table). We further selected common methyl probes in the CTRL and COPD groups within promoter regions and/or CpG islands. The expression data for these groups was comprised of 15,261 mRNA probes (see Methods for details), with each methyl probe mapped to the closest genes corresponding to the mRNA probes (described in Methods). A total of 658,108 methyl probes were located in promoter regions of these mRNA probes and were considered in all future analyses.

### Differentially methylated CpG islands and differentially expressed genes are associated

Prior to integrating the molecular traits, we first characterized the differentially expressed and methylated genes between the COPD and CTRL groups. We identified 1,594 genes as differentially expressed between the COPD and CTRL groups (t-test p-value<0.01, corresponding to a false discovery rate, or FDR, of 0.09 based on permutation tests). We also identified 92,606 methylation probes corresponding to 8,848 genes that were differentially methylated between the COPD and CTRL groups (t-test p-value<0.01, FDR = 0.06 based on permutation tests). There are 990 genes overlapping the set of differentially methylated and expressed genes (Fisher's exact test p-value = 0.009). Given methylation data are known to be noisy [26], [27], we focused on methyl probes located in CpG island defined by a hierarchical hidden Markov model [28], resulting in 26,143 differentially methylated probes corresponding to 6,416 genes for further analyses. Among them, 704 genes overlapped with differentially expressed genes (Fisher's exact test p-value = 6.6×10^−6^). Additional constraints could be applied to further enrich for biologically relevant methylation, such as 1) self-consistent methyl probes (at least two methyl probes differentially methylated for a single gene), and 2) methyl probes close to transcription start sites (<1 kb). These filters were potentially useful for enriching for genes that are both differentially methylated and expressed (S3 Table), but were not used in the analysis because they were too stringent, resulting in smaller signature sizes.

In general, when DNA methylation levels of methyl probes in CpG islands for COPD were compared with those of CTRL samples, the COPD samples were predominantly hypermethylated (S1A Fig.). Results based on probe-by-probe comparison also showed that CpG islands were more likely to be hypermethylated than hypomethylated in lung tissues of COPD patients (S4 Table). However, when comparing non-CpG island methyl probes, the pattern was very different and the numbers of hyper- and hypo- methylated probes were evenly distributed (S1B Fig. and S4 Table). This different pattern suggested there was no global methylation level difference between COPD and CTRL samples. The main difference between them was methylation levels of CpG islands, suggesting biological importance of methylation of CpG islands in transcription regulation. The hypermethylation pattern in CpG islands was observed in a lung cancer study [16]. Recently, Vucic *et al.* reported that DNA methylation levels in COPD were different from ones in CTRL samples and 90% of differentially methylated CpG island probes were hypermethylated in small airways epithelium cells from COPD patients [24]. The differentially methylated or differentially expressed genes were not associated with potential biological subtypes in the samples (S1 Text). These results suggest that there were significant differences in methylation levels between the CTRL and COPD groups and, hence, these differences may be involved in epigenetic regulations causing pathogenesis and progression of COPD.

### Both differentially methylated and expressed genes are related to lung function

Of the 704 differentially methylated genes within CpG islands that are also differentially expressed between the CTRL and COPD groups, most (696 out of 704) were hypermethylated in COPD, whereas only about half of the corresponding gene expression levels (for 378 genes) were downregulated (S5 Table). The remaining 318 genes were upregulated even when their promoter regions were hypermethylated. While this pattern does not match the expected classical inverse relationship between DNA methylation and gene expression levels, a number of studies have shown that the DNA methylation – gene expression relationship may be more complicated [29]–[31]. While promoter methylation most often leads to gene silencing, DNA methylation of promoter regions, in some cases, can be associated with transcription activation; for example through blocking repressor proteins binding to the promoter region [32], [33]. Vucic *et al.* also shows that methylation levels of many genes were positively associated with gene expression levels when comparing small airways epithelium cells of non-COPD controls and COPD patients [24].

Genes that are hypermethylated and downregulated in COPD, including genes related to lung function, such as *EP300, EPAS1, FOXF1, FOXA2, KDR, LAMA5, SHH, NKX2-1, VEGFA, FZD1, NUMB*, and *PKDCC* [34]–[37], are enriched for GO biological processes (S6 Table) such as regulation of cell communication (p-value = 1.98×10^−8^), regulation of multicellular organismal development (p-value = 2.13×10^−7^), and tissue morphogenesis (p-value = 4.43×10^−7^). The other set of genes that are hypermethylated and upregulated in COPD are enriched for a number of GO categories as well, including co-translational protein targeting to membrane (p-value = 2.27×10^−13^), protein targeting to ER (p-value = 3.07×10^−12^), translational initiation (p-value = 5.34×10^−9^), translational termination (p-value = 1.23×10^−8^), and cellular protein complex disassembly (p-value = 1.06×10^−6^) (S7 Table). These results indicate that both epigenetic and transcriptional regulations contribute to COPD pathogenesis. Hence, knowing the causal relationship between DNA methylation and gene expression is critical to understand the complex and systematic molecular underpinnings of COPD.

### The relationships between gene expression and DNA methylation levels are different in the COPD and CTRL groups

**Figure 1:**
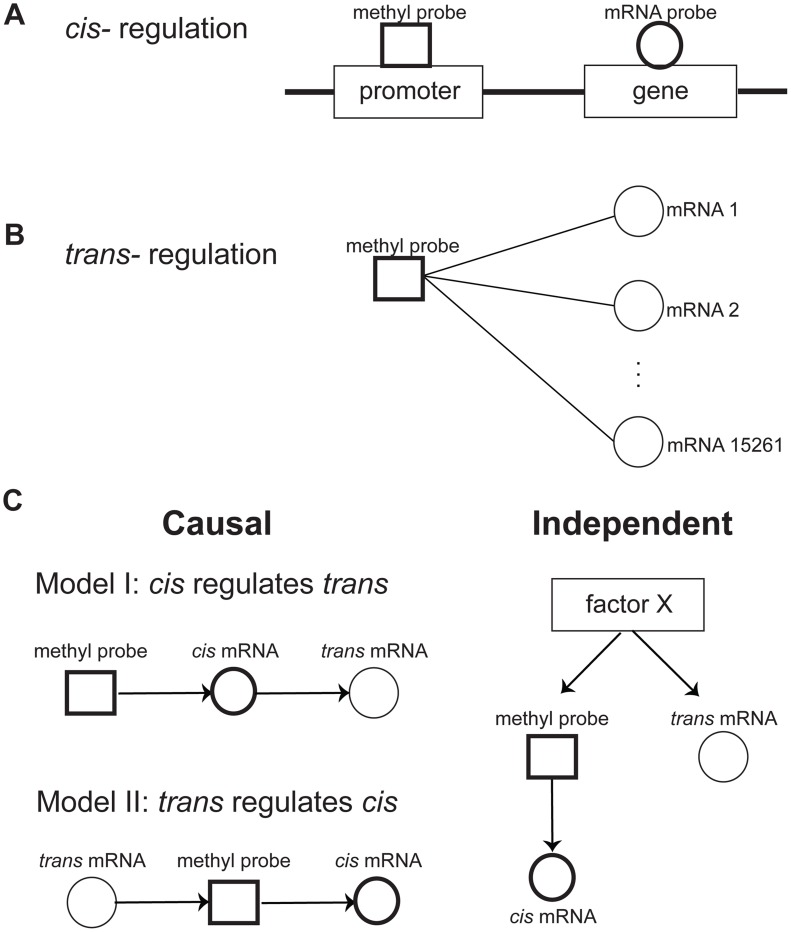
Relationships between DNA methylation and gene expression. **A**) *Cis* regulation was defined by the correlation of the methylation level at the promoter region of a gene with expression level of the gene. **B**) *Trans* regulation was defined by the correlation of a methylation level at the promoter region of a gene with expression level of other genes. **C**) Potential relationships between *cis* and *trans* regulations. There are two potential causal mechanisms of *cis* and *trans* connections: Model I, where the methylation level regulates *trans* gene expression via the *cis* gene expression, and Model II, where *Trans* gene expression regulates the *cis* gene via controlling its methylation level. It is also possible that *cis* and *trans* connections are independently regulated by a factor X.

While CpG islands in COPD are hypermethylated in general, variations in the expression levels of individual genes are mainly influenced by cis-acting methylation levels in a given gene's promoter region [38], [39]. The association of DNA methylation and gene expression was computed in a non-parametric fashion using Spearman correlation statistics [40]. *Cis* regulation was defined as significant correlation between the expression levels of a gene and methylation levels in the promoter region of the gene (Fig. 1A). DNA methylation levels in the promoter region of a gene may also influence the expression of genes that are distal to the given promoter region (*trans* regulation described in Fig. 1B) [41], [42]. At Spearman correlation p-value<0.01, we identified 7,353 and 2,825 *cis* regulated methylation-mRNA probe pairs for COPD and CTRL, respectively. The corresponding *cis* regulated genes significantly overlapped with the 704 differentially methylated and differentially expressed gene set above (Fisher Exact Test p-values = 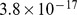 and 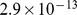 for COPD and CTRL, respectively). We also identified 8,335,177 and 1,338,232 *trans* methylation-mRNA probe pairs at p-value <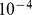 (FDRs = 0.04 and 0.2) for COPD and CTRL, respectively. There were 859,430 and 52,033 *trans* methyl-mRNA probes pairs in COPD and CTRL where a genes's methylation *cis* regulates its own expression and *trans* regulates other genes' expression. These pairs were subjected to the causality test below. While the differences in the numbers of *cis* and *trans* pairs in the CTRL and COPD groups may be at least partially due to power differences (52 versus 100 sample pairs in CTRL and COPD, respectively), we observed similar differences after constraining each group to have the same number of samples (S2 Fig.). There are 218 *cis* pairs in common between the CTRL and COPD groups, a statistically significant enrichment (Fisher's exact test p-value = 1.6×10^−7^). However, there are only 171 *trans* pairs shared between the CTRL and COPD groups (Fisher's exact test p-value = 1). Therefore, the relationships between gene expression and DNA methylation are likely different between the COPD and non-disease CTRL groups.

### Methylation variation is generally causal for *trans* gene expression

**Figure 2:**
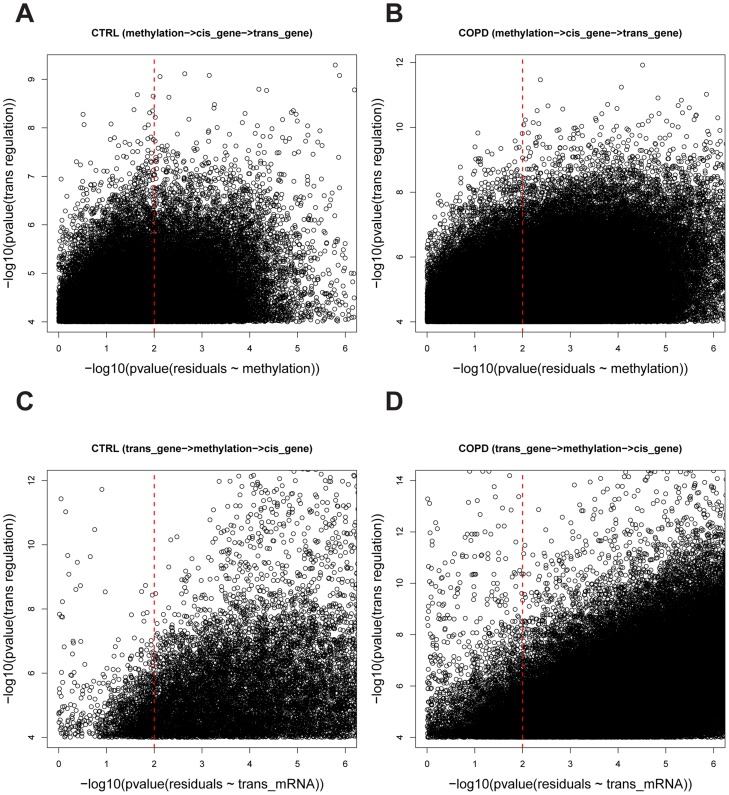
The causality test of *trans* methyl-mRNA pairs. **A**) and **B**) are causality test results for the causal model whereby methylation regulates *trans* gene expression (methylation 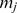
→ *cis* gene expression 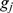
→*trans* gene expression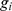) in control and COPD data sets, respectively. The Y-axis is the –log10 of the p-values for the Spearman correlation between 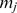 and 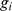 and the X-axis is –log10 of the p-values for the Spearman correlation between 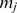 and 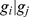. A causal relationship (methylation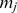 → *cis* gene expression 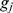
→*trans* gene expression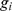) was defined if the p-value of 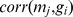 was <0.0001 and the p-value of 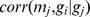
was>0.01 (see Methods for details). A total of 30,177 and 362,095 causal pairs were inferred in control and COPD samples, respectively. **C**) and **D**) are the causality test results for the causal model whereby *trans* gene expression regulates methylation variation (*trans* gene expression
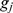
→methylation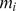 → *cis* gene expression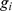) in control and COPD data sets, respectively. The Y-axis is the –log10 of the p-values for the Spearman correlation 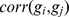
and the X-axis is –log10 of the p-values for the Spearman correlation 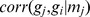. A causal relationship (*trans* gene expression
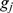
→methylation
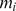
→ *cis* gene expression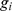) was defined if the p-value of 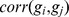
was <0.0001 and the p-value of 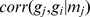 was>0.01 (see Methods for details). A total of 1,241 and 19,173 causal pairs were inferred in control and COPD, respectively.

While it is reasonable to assume that methylation variation in the promoter region of a gene is causal for changes in the gene's expression (*cis* regulation), changes in gene expression may also be causal for methylation changes via *trans* regulations that can affect processes such as the transfer of methyl groups [43], [44]. These can be represented as two possible causal models of *cis* and *trans* regulation (shown in Fig. 1C): in model I, the expression of a *trans* gene is regulated by gene expression that is *cis* modulated by variations of the methylation levels in the corresponding gene's promoter region; and model II, the methylation levels for a gene in *cis* is regulated by the expression level of a gene in *trans*. In addition to these causal relationships, the *cis* and *trans* regulation can be independent, regulated by an unknown factor X (model III). To infer the causal relationship between gene expression and methylation variations, we developed a causality test similar to previously developed causality tests [45]–[47] (see Methods for details). By applying the causality test, we identified 30,177 *trans* pairs in the CTRL group (FDR = 0.03) and 362,095 *trans* pairs in the COPD group (FDR = 0.0014) whose methylation levels likely regulated the expression of *trans* genes (Fig. 2A and 2B). For all of these *trans* pairs, the strong correlation between methylation and *trans* gene expression was predicted by our modeling to be mediated by the expression levels of the *cis* gene (Model I in Fig. 1C). Of the putative causal relationships identified by our approach, only 1,241 and 19,173 *trans* gene-methylation pairs identified in the CTRL and COPD groups, respectively, were of the Model II type (Fig. 1C) in which *trans* gene's expression → methylation in *cis* gene → *cis* gene's expression (Fig. 2C and 2D). These results indicate that the association between *trans* gene expression and methylation in a *cis* gene, is overwhelmingly driven by changes in *cis* gene expression which is regulated by methylation changes in the *cis* gene.

### Key regulators in CTRL and COPD lung

**Figure 3:**
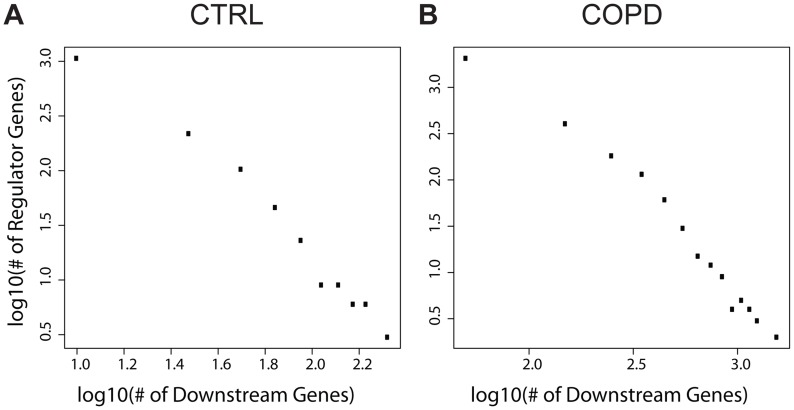
The numbers of downstream genes regulated by DNA methylation level variation follow a scale-free distribution (a linear relationship in log-log plots). **A**) The numbers in control; **B**) The numbers in COPD.

We next assessed whether there were any epigenetic hotspots in which the expression levels of many genes in *trans* varied as a consequence of a single *cis* gene whose expression levels were altered by methylation events in *cis*. Such *cis* genes can be considered as key regulator genes. Towards this end we characterized the number of trans genes causally associated with *cis* genes as determined by the causality test above and found that the numbers of *cis* genes and their causally regulated *trans* genes follow a scale-free distribution (Fig. 3; linear in the log-log plot). That is, most *cis* genes regulate a small number of *trans* genes, but there are a few *cis* genes that regulate a large number of *trans* genes as downstream targets. We defined key regulators as genes whose number of downstream targets is larger than the mean plus two standard deviations across all *cis* genes. Given this definition, we identified 67 genes as key regulators in the CTRL group and 126 in the COPD group (S8–S9 Tables). These key regulators influence a significant number of downstream genes. There are 6 regulators in common between the CTRL and COPD groups: *FOXK2*, *HEATR2*, *EPAS1*, *PLXNB2*, *GAK*, and *YOD1*. However, only a small portion of their downstream target genes is shared, with the biological enrichments revealed by these downstream targets are different between the CTRL and COPD groups. These results suggest that epigenetic regulation of gene expression mediated by DNA methylation has different biological consequences between the COPD and CTRL groups. More detailed analyses of these key regulators in terms of methylation patterns, downstream targets, and their regulated biological processes are presented in the S1 Text. In summary, we reveal that multiple key regulators target similar sets of genes indicating that the epigenetic control of gene expression by methylation is seemingly complex (S3–S4 Fig.). The patterns were similar if more stringent definition (the number of downstream targets> the mean plus 3 standard deviations) of key regulator was used (S5 Fig.). There were multiple key regulators in COPD regulating genes involved in metabolic processes and immune response, which are processes known to be involved in COPD pathogenesis and progression.

### *EPAS1* is the only key regulator consistently associated with multiple COPD disease severity traits

To further investigate key regulators in COPD development and progression we compared the expression levels of the key regulators and their downstream genes with genes associated with COPD disease severity related clinical features. Five COPD related severity measures were available in the LGRC data set, including DLCO (**D**iffusing capacity of the **L**ung for **C**arbon **M**onoxide) [48], BODE (**B**ody mass index, airflow **O**bstruction, **D**yspnea and **E**xercise capacity) index [49], FEV1 (**F**orced **E**xpiratory **V**olume) percentage predicted [50], FEV1/FVC (**F**orced **V**ital **C**apacity) ratio, and emphysema percentage. DLCO, FEV1 percentage predicted, and FEV1/FVC ratio decrease as COPD severity increases, while BODE index and emphysema percentage increase with disease severity.

**Figure 4:**
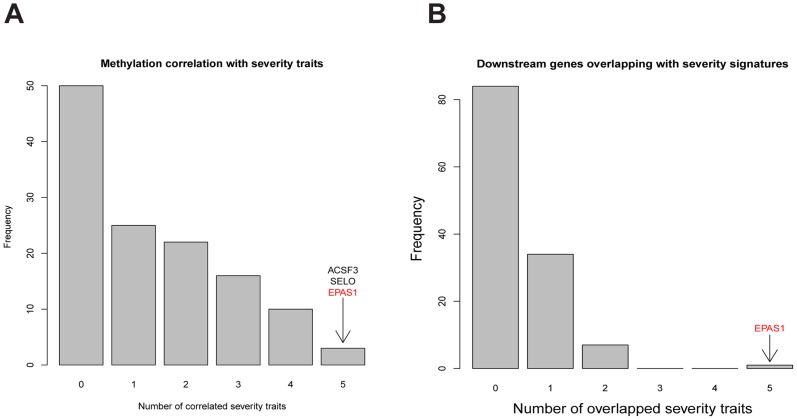
Comparing characteristics of key regulators with 5 COPD severity related traits in LGRC. **A**) Comparing lung DNA methylation profiles of key regulators with 5 COPD severity related traits by Spearman correlation. At the Fisher's exact test p-value <0.05, the DNA methylation level variations of 3 key regulators, *ACSF3*, *SELO*, and *EPAS1*, were correlated with all 5 COPD severity related traits. **B**) Comparing downstream genes of key regulators with gene signature sets for 5 COPD severity related traits by the hypergeometric test. At the Fisher’s exact test p-value<0.05, only the key regulator *EPAS1*'s downstream genes significantly overlapped with gene signature sets for all 5 COPD severity related traits.

At p-value<0.05, methylation levels of the promoter regions of 3 of the 126 key regulators in COPD groups, *ACSF3*, *SELO*, and *EPAS1*, significantly correlated with all 5 disease severity phenotypes (Fig. 4A; S10 Table). At p-value<0.01, we identified 572 expression traits in the COPD group as significantly correlated with DLCO (FDR = 0.24), 1164 genes with BODE (FDR = 0.12), 545 genes with FEV1 percentage predicted (FDR = 0.27), 333 genes with FEV1/FVC (FDR = 0.40), and 1702 genes with emphysema percentage (FDR = 0.09). There was no key regulator gene whose expression levels consistently correlated with all 5 COPD severity phenotypes. Therefore, to strengthen the association between key regulators and COPD, we compared the disease phenotype gene expression signature sets with each of the key regulator's downstream targets (Fig. 4B; S11 Table). Of the 126 key regulator genes, *EPAS1* was the only gene whose downstream genes were significantly overlapping with all disease phenotype gene expression signature sets (Fig. 4B).

We further compared the downstream genes of key regulators in COPD with known COPD signatures. Recently, Campbell *et al.* reported a set of 127 genes whose expression levels were significantly associated with regional emphysema severity in a mouse model [51]. Our human mRNA dataset includes 104 orthologous genes out of these 127 mouse emphysema severity associated genes. When directly comparing the emphysema associated genes in mouse and our emphysema percentage related genes in human, only 10 of them overlap (Fisher's exact test p-value = 0.76). When comparing these 104 genes to the downstream target genes of all key regulators in COPD, only the downstream genes of *EPAS1* significantly overlap with this emphysema severity associated gene set (S12 Table); *EPAS1* itself is one of the emphysema severity associated genes in mouse. Among the 104 emphysema severity associated genes in mouse, 30 of them overlap with the downstream genes regulated by *EPAS1* (p-value = 5.1×10^−15^). Expression levels of 4 of the 30 overlapping genes are positively correlated with *EPAS1* methylation levels indicating that their expression levels increase as emphysema severity increases. One of the four genes, *CD79B,* was positively correlated with *EPAS1* methylation levels, which is consistent with previous reports that B cell abundance increases as emphysema severity increases [51], [52]. Expression levels of the remaining 26 genes are anti-correlated with *EPAS1* methylation levels; their expression levels are expected to decrease as emphysema severity increases. For example, gene expression levels of members of the TGF-beta pathway such as *ACVRL1* are inversely correlated with *EPAS1* methylation levels. This observation agrees with previous reports in which *TGFBR2* was shown to be down regulated in regions of severe emphysema [53].

### *EPAS1* regulates a unique and significant set of downstream genes in COPD

*EPAS1*'s methylation profile and its downstream genes are distinct from ones of other regulators (S4 Fig.). Only 6% of downstream targets of the key regulator *GAK*, which regulated the largest number of downstream target genes, overlapped with the *EPAS1* downstream target genes (Fisher's exact test p-value = 1). *EPAS1* downstream target genes are enriched for multiple GO biological processes (S13 Fig.) including anatomical structure formation involved in morphogenesis (p-value = 1.17×10^−6^), adherens junction assembly (p-value = 3.53×10^−6^), locomotion (p-value = 5.92×10^−6^), angiogenesis (p-value = 1.22×10^−5^), and cell division (p-value = 1.52×10^−5^). *EPAS1* is differentially expressed and methylated between the CTRL and COPD groups (S6 Fig.). The putative causal relationships identified between *EPAS1* and *trans* genes associated with methylation changes in the *EPAS1* promoter region, the association of *EPAS1* with COPD severity measures, and its differences between the CTRL and COPD groups indicate that *EPAS1* is a putative key causal regulator of multiple COPD severity phenotypes in human and emphysema severity associated genes in mouse.

### *EPAS1* downstream genes overlap with hypoxia responsive genes in pulmonary artery endothelial cells

*EPAS1* is a hypoxia-responsive transcription factor and is also known as Hypoxia-inducible Factor 2 alpha (HIF2α) [54], . It is regulated by oxygen through enzymatic post-translational hydroxylation of the α subunit [56]. With a sufficient supply of oxygen, HIF genes are degraded. But under hypoxic conditions, HIF genes bind directly to DNA and enhance transcription of target genes [57], [58]. While several studies have revealed that HIF2α has been implicated in cancer [59]–[62], the specific physiological functions of *EPAS1* are not yet fully understood. There have been several studies regarding hypoxic response genes in different tissues including breast, kidney, head and neck, and lung [63]–[67]. From these data we found that our predicted *EPAS1* downstream target genes significantly overlapped with HIF regulated genes only in primary human pulmonary artery endothelial cells (Fisher's exact test p-value = 0.004) [63], but not with the other hypoxia signatures defined in other tissues such as breast cancer, head and neck cancer, and normal kidney (p-values = 0.74, 0.24, and 0.15, respectively). These results suggest that the regulation of hypoxia responsive genes by *EPAS1* may be a unique characteristic of COPD lung samples. In addition to directly binding to HIF response elements, *EPAS1* may regulate downstream gene expression by regulating or interacting with other transcription factors such as *AREB6*/*ZEB1* or miRNAs (see S1 Text).

### EPAS1 protein abundance is lower in lung tissues of COPD patients

*EPAS1* expression levels are lower in COPD lung tissue compared to CTRL lung (S6A Fig.). To test whether EPAS1 protein abundance concordantly changes with *EPAS1* gene expression levels in lung tissues of COPD patients, we stained lung tissue blocks from 5 COPD patients and 4 non-COPD patients using a polyclonal anti-EPAS1 antibody (NB10-122; Novus Biologicals, CO, USA) and categorized EPAS1 abundance. All 4 slides from non-COPD patients contained high levels of EPAS1, and 3 of 5 slides from the COPD patients contained low levels of EPAS1 as shown in S7 Fig., so that a statistically significant difference in EPAS1 protein levels was observed between the two groups (p-value = 0.03). The difference was similar for endothelial cells (EPAS1 high in 4 of 4 non-COPD samples and low in 3 of 5 COPD samples) and alveolar (EPAS1 high in 4 of 4 non-COPD samples and low in 3 of 5 COPD samples) cells.

### *EPAS1* expression levels are lower in lung tissue of mice chronically exposed to smoking

**Figure 5:**
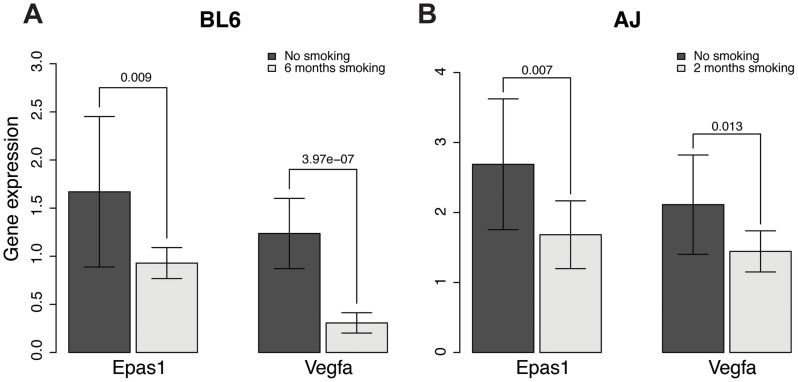
Gene expression levels of *Epas1* and *Vegfa* were lower in chronic smoking mice than non-smoking age-matched mice at the time when COPD develops in different mouse models. **A**) Gene expression levels of *Epas1* and *Vegfa* in C57BL/6J mice that develop COPD after 6 months chronic exposure to cigarette smoke. **B**) Gene expression levels of *Epas1* and *Vegfa* in A/J mice that develop COPD after 2 months chronic exposure to cigarette smoke. The t-test was used to compare *Epas1* or *Vegfa* expression levels in mice with or without chronic smoke exposure.

The *EPAS1* target genes we predicted significantly overlap with genes associated with emphysema caused by smoking in mouse, as indicated above. To investigate whether *EPAS1* expression levels change when mice start to develop emphysema after chronic smoking exposure, we checked *Epas1* expression levels in two different chronic smoking mouse models using C57BL/6J and A/J mice. C57BL/6J mice start to develop emphysema after 6 month exposure to chronic smoking [68] while A/J mice start to develop emphysema after only 2 months of exposure to chronic smoking [69]. *Epas1* expression levels in smoking mice (6 months of smoking for C57BL/6J and 2 month for A/J) are significantly lower than levels in corresponding age-matched non-smoking mice (Fig. 5, p-value of the t-test = 0.009 and 0.007 for the C57BL/6J and A/J models, respectively). We also checked the *Epas1* downstream target gene vascular endothelial growth factor (*Vegfa*), given it is also a hypoxia responsive gene. Smoke exposed mice had lower amount of *Vegfa* expression as well (Fig. 5, p-value of the t-test = 4.0×10^−7^ and 0.01 for the C57BL/6J and A/J smoking models, respectively), which suggests that *Epas1* downstream target genes were down regulated in the smoke exposed mice at the time when emphysema develops in these models. These results are consistent with our causal predictions relating to *EPAS1.*

### The *EPAS1* knockdown signatures in human and mouse endothelial cell lines match the predicted EPAS1 downstream target genes

To test whether *EPAS1* causally regulates the downstream target genes we predicted, we knocked down *EPAS1* expression via siRNA in human umbilical vein endothelial cells (HUVEC) and mouse endothelial cell line C166 (see Methods for details) and then performed RNASeq analysis to quantify genome wide gene expression changes. When comparing endothelial cells treated with *EPAS1* siRNAs and scrambled siRNAs, we identified an *EPAS1* siRNA signature consisting of 2796 and 3730 genes in human and mouse endothelial cell lines, respectively, whose expression levels significantly changed (t-test p-value<0.05), including *EPAS1* itself (p-value = 0.002 and 0.02) and the *EPAS1* downstream target gene *VEGFA* (p-value = 0.03 and 0.01). The *EPAS1* siRNA signatures derived from human and mouse cell lines were highly consistent, with 695 genes in common to both signatures (p-value = 7.2×10^−65^). Both signatures not only significantly overlapped with *EPAS1* downstream genes (p-value = 7.3×10^−7^ and 1.5×10^−12^), but also with hypoxia response genes in endothelial cells (Fisher's Exact Test p-value = 5.8×10^−8^ and 1.2×10^−12^ in the human and mouse signatures, respectively). Moreover, the *EPAS1* siRNA signatures consistently overlapped genes associated with the COPD severity phenotypes (Table 1). These results together validate that *EPAS1* causally regulates the downstream target genes we predicted, and that these genes in turn affect COPD development and progression.

**Table 1:** *EPAS1* siRNA signatures in human and mouse endothelial cells overlap with multiple COPD disease severity related signature sets.

	BODE	DLCO	FEV1	FEV1/FVC RATIO	RCL Emphysema Percentage	Emphysema signatures (mouse)
**Human siRNA (2796)**	179 (p = 6.7e-8)	85 (p = 0.001)	73 (p = 0.02)	44 (p = 0.11)	291 (p = 4.2e-20)	29 (p = 4.4e-5)
**C166 siRNA (3730)**	235 (p = 6.9e-13)	115 (p = 5.5e-6)	86 (p = 0.038)	58 (p = 0.027)	318 (p = 7.4e-16)	28 (p = 0.004)

## Discussion

Genetic, epigenetic, and environmental factors are known to contribute to COPD risk and disease progression. Therefore to elucidate more comprehensive molecular regulations of COPD disease, we developed a novel systematic approach to identify key regulators in COPD and CTRL lung tissue by integrating genome-wide DNA methylation and gene expression patterns. Using our causality test, we link the variation of the expression of numerous genes to only a few key regulators that are systematically regulated by variations in DNA methylation including 126 for COPD and 67 for non-disease lung. These key regulators such as *EPAS1* can be targets of potential therapeutic intervention.

We also highlighted important biological pathways associated with these key regulators in normal and diseased lung by hierarchical clustering of their common downstream genes. We observed common epigenetic regulations in both CTRL and COPD samples in expression of genes involved in metabolic- and cilium related- biological processes. Although cilium-related genes display the most varying expression levels both in CTRL and COPD samples they are not associated with disease phenotypes. This is an interesting observation as in the lung ciliary-related proteins keep the airways clear of mucus and dirt, allowing one to breathe easily and without irritation. Key regulators of these genes are *WDR90* in CTRL and *PAX9* in COPD. Since mRNA levels of *PAX9* are associated with *WDR90* methylation in CTRL, this suggests the wide variance of expression of the cilium related genes are explained by epigenetic regulations via methylation level of the regulators in the same pathway.

Similarly, we observed common epigenetic regulations with metabolic processes, including RNA processing and chromatin modifications, by key regulators both in CTRL and COPD. However, unlike the ciliary-related pathway; the key regulators are not exactly the same for COPD and CTRL. These observations highlight in part potential mediators of COPD pathophysiology. In COPD, there are three groups of key regulators obtained based on their shared downstream genes (S4B Fig.). The key regulators in the two large clusters control similar downstream genes involved in metabolic processes, RNA processing, chromatin modification, immune response and cell cycle. This type of coordinated yet diverse pathway regulation seems fitting with the current view of COPD, in that the disease pathologically is not limited to the lungs, but rather a disorder with systemic features. This view is driven in part by the strong associations of COPD with increased CVD risk, anemia, musculoskeletal diseases as well as the metabolic syndrome and Type 2 diabetes mellitus [70]. While the underlying molecular basis linking COPD with these comorbidities is still not fully understood, alterations in several pathophysiological features have been considered important such as systemic inflammation, oxidative stress, adipokine metabolism, insulin resistance and obesity.

Importantly, beyond the pathway level, we were able to identify genes of importance through looking at the key regulators associated with these cluster. One interesting gene was *GAK*, as it was predicted to regulate the largest number of downstream genes. *GAK* is a cyclin G associated kinase, and is known to regulate clathrin-mediated membrane trafficking [71]. Recently, it has also been shown that the disruption of the kinase domain of *GAK*, in mice, causes embryonic lethality due to pulmonary dysfunction including notable alterations in the distribution of lung surfactant protein A [72], a known biomarker of COPD disease severity [73]. These studies in mice were prompted by the fact that gefitinib, which is an inhibitor of the epidermal growth factor receptor and used to treat non-small cell lung cancer in humans, has significant adverse side effects in therapy, such as respiratory dysfunction, which in part has been attributed to the fact the gefitinib also inhibits *GAK* [72]. While a role for *GAK* in COPD has not been previously linked, our observations would suggest further investigation is warranted. Importantly, about 87% of key regulators in COPD (111/126) share similar downstream genes with *GAK*. In addition, some of their methylation levels are highly correlated each other, indicating overall that regulation of downstream genes may be mediated by multiple key regulators in a systematic way rather than by single master controllers.

Other potentially relevant mediators of COPD pathophysiology are those key regulators that showed a different methylation profile and different downstream target gene set as compared to all other regulators, such as was the case with *EPAS1*. To our knowledge *EPAS1* has not been previously linked with COPD pathophysiology. This is despite the fact that *EPAS1* is one of the major mediators of the transcriptional response to physiological hypoxia, an environment typical of lung alveolar as progressive airflow limitation increases with COPD severity. *EPAS1* is a hypoxia-responsive transcription factor and is also known as hypoxia-inducible factor 2 alpha (HIF-2α) [54], [55]. Interestingly, compared to the ubiquitous expression of *HIF1a*, another key mediator of hypoxic responses, *EPAS1* has relatively high levels of expression in the placenta, heart, lung and endothelial cells. Importantly, a previous study reported alveolar hypoxia increases in prevalence as disease severity increases [74] and mounting evidence suggests, hypoxia is more than a signifier of advanced COPD but rather a key player in many of the maladaptive processes as well as the systemic comorbidities associated with COPD. Since sustained exposure of cultured lung alveolar epithelial cells to hypoxia maintained the induction of *EPAS1* expression as induced by short-term hypoxic exposure, the decreased *EPAS1* expression observed in COPD may in fact result in maladaptive hypoxia responses [75]. Thus understanding the contribution of *EPAS1* to disease and its mechanisms in it would be very promising for treatment of disease.

In this study we demonstrate that *EPAS1* methylation level is significantly associated with disease severity and that an increase in methylation decreases *EPAS1* gene expression. Thus we hypothesize that disease severity may be systematically controlled by altered regulation of a large set of *EPAS1* downstream genes. Several observations in humans and mouse have demonstrated that altered *EPAS1* expression can affect lung physiology. Specifically gain-of-function mutations in humans were associated with pulmonary hypertension, increased cardiac output and heart rate as well as increased pulmonary ventilation relative to metabolism [76]. However, in a heterozygous *EPAS1* mutant mouse, haploinsufficiency for the oxygen-sensing factor resulted in augmented carotid body sensitivity to hypoxia, including irregular breathing, apneas, hypertension and elevated norephinephrine levels on one mouse strain background, but protection against pulmonary hypertension on a different strain [77], [78]. There are several consequences of hypoxia in COPD which may contribute to disease severity, with pulmonary hypertension in part due to hypoxic pulmonary vasoconstriction driven by alveolar hypoxia, being one of them.

Another possible link between hypoxia mediated COPD disease severity and *EPAS1* may be the fact that *EPAS1* is a known transcriptional activator of the VEGF [55], which was shown in our study to be one of *EPAS1* downstream genes and one of EPAS1 siRNA signature genes. *VEGF* expression level is associated with COPD phenotypes and downregulated in COPD samples in the LGRC dataset. *VEGF* is involved both in the regulation of the bronchial microvascular changes as well as in the inflammatory airway changes in COPD. In patients with emphysema, low levels of VEGF are thought to promote the destruction of alveoli, since VEGF normally acts to induce the expression of anti-apoptotic proteins and acts as a survival factor for endothelial cells. The importance of VEGF in survival signals necessary for the maintenance of normal lung structure and consequences characteristics of emphysema has also been confirmed in animal studies disrupting VEGF signaling either through genetic deletion of lung *VEGF* or through VEGF receptor blockage. *VEGF* is also thought to play a dual role in the lung by regulating not only apoptosis but also efferocytosis, which is the process involved in phagocytosis of apoptotic cells. The net effect of efferocytosis is anti-inflammatory because dying cells are removed before they undergo postapoptotic necrosis and anti-inflammatory mediators are released thereby suppressing further adaptive immune responses. Therefore, dysregulation of *VEGF* via altered *EPAS1* regulation could link hypoxia to mechanisms of COPD severity [79].

One other point of interest is the fact that neonatal mice lacking complete *EPAS1* expression have deficient lung surfactant, such as surfactant D (SP-D), in addition to other lung abnormalities and die of respiratory failure [80]. This deficiency has been attributed to reduced expression of *VEGF* as VEGF rescue therapy resulted in restoration of surfactant production and less respiratory distress in the *EPAS1* null mice compared to wildtype. Surfactants, such as SP-D have many functional properties including anti-inflammatory and anti-oxidant capacities, and protection against respiratory infections. In various mouse models, SP-D appears to play a distinct role in protecting murine lungs from the development of emphysematous changes possibly by reducing inflammation and oxidative stress in the lungs. While in humans, elevated serum SP-D level is an apparent biomarker of COPD, there is a reported inverse relationship with bronchoalveolar lavage fluid levels, whether elevated or decreased levels of SP-D are important in pathogenesis are still unclear [81]. Nonetheless, this is another clear example of how *EPAS1*, through modulation of *VEGF*, may contribute to the chronic inflammatory response and tissue destruction in COPD through augmented apoptosis, impaired efferocytosis, and abnormal tissue remodeling.

Many studies focus on genetic contribution to COPD development and phenotypes [82]–[84] and a recent review paper provides an updated list of COPD associated genes [85]. There are 140 COPD susceptible genes identified in at least one of COPD GWAS studies. When we overlapped these CODP susceptible genes with *EPAS1* downstream genes, the overlap is marginal significant (Fisher's exact test p-value = 0.053), but it is the best overlap comparing with other regulator's downstream genes (the second best p-value is above 0.1). This enrichment of COPD GWAS genes in *EPAS1* downstream further substantiates critical role of *EPAS1* in the disease.

At present it is still unclear how the methylation level of key regulators, in particular the predominant hypermethylation seen in COPD is regulated upstream. A recent study has also demonstrated that DNA methylation is widely disrupted and predominantly hypermethylated in small airway epithelia of COPD patients [86]. In addition to cigarette smoking, evidence has shown that hypoxia is also an important regulator of a cell's global epigenetic profile. For an example, chronic hypoxia induces a significant increase in global DNA methylation such as in human pulmonary fibroblasts [87]. Some of the underlying mechanisms that may account for global epigenetic alterations in DNA methylation include changes in the activity of epigenetic modifying enzymes such as DNA methyltransferases (DNMT) or in levels of the methyl-donor S-adenosylmethionine (SAM). DNA hypermethylation has also been demonstrated in PwR-1E prostate cell cultures in response to chronic hypoxia, a consequence linked to increased *de novo* DNMT activity due to elevated expression of *DNMT3B* as well as a hypoxia-induced decrease in levels of SAM suggesting an increase in SAM usage in hypoxic cells [88]. Interestingly, low circulating levels of folate and increased homocysteine levels, which are involved in the generation of SAM via the one-carbon cycle, have been associated with COPD patients [89].

Compared with the number of inferred relationships that methylation variations causally regulate gene expression (methylation → gene expression) in *trans*, the number of inferred relationships that gene expression variations causally regulate methylation variations (gene expression →methylation) in COPD is small (362,095 vs. 19,173). Similar to the methylation → gene expression relationships, the numbers of genes' methylation levels regulated by a gene's expression level in *trans* follow a scale-free distribution (S8 Fig.). The top putative causal regulator *CDK5RAP1* controls methylation levels of 152 genes in COPD. *CDK5RAP1* is a RNA methyltransferase [90]. Both DNA and RNA transmethylations are affected by the availability of the universal methyl donor substrate S-adenosylmethionine (SAM). Interesting, 44 of 126 key methylation → gene expression regulators overlap with *CDK5RAP1* downstream genes (p-value = 4.9×10^−57^). And among the 44 genes in the overlap, 32 genes methylation levels negatively correlate with *CDK5RAP1* expression level and 12 of them positively correlate. This result suggests that one possible mechanism *CDK5RAP1* regulating methylation levels of key COPD regulators is through affecting availability of SAM.

It is worth to note that there are differences between statistical causal and biological causal relationships. Similar to other causal inference studies [45]–[47], [91], all causal relationships inferred from the causality test in this study imply statistical causal relationships. Perspective validations are needed to convert statistical relationships into biological causal relationships [92]. It is also worth to note that the causal relationship 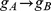 does not imply gene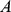 regulates gene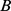 by direct physically interact even the causal relationship is biologically validated. Gene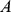 might regulate gene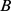 through gene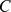.

There are some limitations in the array-based technologies used for measuring gene expression and methylation profiles. Transcript levels of different splicing isoforms were not uniquely measured in the Agilent arrays. Different splicing isoforms of genes, such as *NOD2* [93] and *RAGE* [94], associate with COPD severity and progression. Differential splicing is as prevalent as differential gene expression based on RNAseq analysis of other complex lung diseases such as idiopathic pulmonary fibrosis [95]. Similarly, methylation arrays can't differentiate methylation forms of cytosine, 5-methylcytosine (mC) and 5-hydroxymethylcytosine (hmC). DNA demethylation in mammals involves oxidizing mC to hmC followed by deamination or oxidation steps [96]. It was shown that hmC can offset mC's repression on gene expression [97] and hmC plays an important role in embryogenesis and brain development [98]. However, hmC level in lung tissues is low [99] so that we can assume that the DNA methylation level measured by arrays was mainly due to mC level. RNA sequencing technologies are needed to precisely quantify contributions of transcript splicing isoforms or hmC levels in COPD pathogenesis or progress.

In summary, we propose a potential epigenetic mechanism of COPD using a novel systematic approach integrating *cis* and *trans* regulation between DNA methylation and gene expression. This approach provides mechanisms of how variation of the expression of genes is systematically regulated by DNA methylation level of key regulators in COPD. The severity of COPD can be regulated by methylation level of *EPAS1* and, in turn, it regulates large numbers of gene expression variations. Therefore, if lowering methylation level of *EPAS1* or increasing *EPAS1* expression level might be very useful to treat patients with this irreversible disease. This approach can be applied to other diseases where DNA methylation can contribute to disease development such as lung cancer to find key epigenetic contribution to the diseases.

## Methods

### Samples, gene expression and DNA methylation data in the LGRC

The LTRC is a resource program of the NHLBI that provides human lung tissues to qualified investigators for use in their research. The program enrolls donor subjects who are anticipating lung surgery, collects blood and extensive phenotypic data from the prospective donors, and then processes their surgical tissues for research use. The diagnoses of COPD are based on clinical, imaging and pathological data including chest CT images, pulmonary function tests, exposure and symptom questionnaires, and exercise tests. The COPD class in this study was based on having a FEV1/FVC<.7 on pulmonary function testing. The “control” lungs consist of adjacent histologically normal lung tissues obtained at time of nodule resection from patients with normal lung function testing parameters. In terms of tissue collection procedure, all lung tissue cores were collected at the time of surgical resection, surgical biopsy or transplantation and flash frozen in liquid nitrogen prior to being stored at −80.

Data used in the study were obtained from the publicly available LGRC data portal (http://www.lung-genomics.org). All LGRC lung mRNA data were generated using Agilent V2 human whole genome arrays and were deposited into GEO database as GSE47460 by LGRC consortium. All RNA samples subjected to gene expression profiling were with RIN>7.0. Due to the number of samples, multiple batches of arrays were necessary, so 10% of the arrays were picked at random to have replicates throughout each batch to account for possible batch effects. The feature extracted data was normalized using a pairwise cyclic loess approach, and the probes were collapsed to one probe per gene by selecting the probe with the highest average signal. The processed mRNA arrays data were directly downloaded from the LGRC data portal.

DNA methylation data were generated using Nimblegen 2.1 M Whole-Genome Tiling array. The quality of each probe was compared with the background probe signals and probes with low quality were removed from the dataset. The DNA methylation level (β value) of each tiling probe was estimated using the CHARM method [27], [100]. The estimated methylation level for each sample from the raw data was almost identical with the processed methylation level downloaded from the LGRC data portal. For COPD and controls there are 218 and 94 gene expression arrays, respectively. There are 179 and 76 methylation arrays for COPD and controls, respectively. To check for potential errors in labeling of the sample name, we applied the MODMatcher (Multi-Omics Data Matcher) procedure to identify matched methylation and gene expression samples based on the assumption that the correlation of methylation mRNA profiles from the same individual was significantly higher than ones from randomly paired samples [25]. The matching result was stable after 25 iterations of sample alignments with 100 COPD sample pairs and 52 control sample pairs selected for further analysis (S14 Table). The demographic characteristics of these samples are listed in S1 Table. Both gene expression and methylation profiling data were adjusted for covariates as 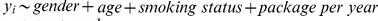 where 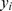 is gene
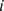
's expression or methylation level. Means plus residuals were used for further analysis.

Potential biological subtypes in the samples were compared with disease status S9 Fig., S15–S19 Tables, detailed in S1 Text).

### Disease phenotypic information in LGRC

There are 5 different measurements of lung function for patients in the LGRC cohort: 1) DLCO (**D**iffusing capacity of the **L**ung for **C**arbon **M**onoxide) [48], 2) BODE (**B**ody mass index, airflow **O**bstruction, **D**yspnea and **E**xercise capacity) index [49], 3) FEV1 (**F**orced **E**xpiratory **V**olume) percentage predicted [50], 4) FEV1/FVC (**F**orced **V**ital **C**apacity) ratio, and 5) emphysema percentage. For each clinical phenotype, only a part of COPD patients were measured: 85 for DLCO, 98 for BODE, 81 for FEV1 and FEV1/FVC ratio, and 62 for the emphysema percentage.

### Mapping of methyl probes to corresponding genes

Each methyl probe was mapped to the nearest transcript starting site. Transcription information of hg18 was fetched from UCSC Genome browser database and further processed using the Bioconductor GenomicFeature package. A probe was mapped to the nearest gene if the distance between the probe and the nearest gene's transcription starting site in was less than 10 kilobases.

### Estimating False Discovery Rates (FDRs) based on permutation tests

FDRs were estimated in multiple statistic tests based on permutation tests. For differential gene expression analysis between COPD and control samples, we permuted sample labels (COPD or CTRL), then applied the t-test to the permuted data to identify significant differentially expressed genes. We performed the permutation test 100 times to estimate FDRs. Similarly, for differentially methylation analysis between COPD and control samples, we permuted sample labels, and applied the permutation scheme 100 times to estimate FDRs for differentially methylated genes at each p-value of the t-test.

For estimating FDRs of *cis* or *trans* acting methylation-mRNA probe pairs in COPD or control samples we permuted genome-wide gene expression data 5 times, calculated pairwise correlation between methylation and permuted gene expression profiles for all possible pairs, and then counted *cis* or *trans* acting pairs in permuted data at each p-value cutoff. Similarly, for association analysis between gene expression and phenotypical data we permuted genome-wide gene expression data 5 times, calculated pairwise correlation between gene expression and phenotypical data for all possible pairs, and then counted significant pairs in permuted data at each p-value cutoff.

### Gene Ontology (GO) analysis

To identify potential functions of selected gene sets, we compared these gene sets with each GO biological process [101] and computed functional enrichment using the hypergeometric test. For the annotation, Agilent hgug4845a annotation data corresponding to the mRNA microarray was used in the Bioconductor GOstats package [102]. The embedded function called “geneIdsByCategory” was used to fetch the list of genes overlapping with each GO term. Any GO biological process consisting of more than 1500 genes was considered non-specific and was removed from the analysis.

### The causality test for determining the relationship between methylation and gene expression

For simplification purposes, we describe the causality test using the COPD dataset, the corresponding values for the control dataset were generated in a similar fashion. Given a significant *cis* methylation-mRNA relationship for gene 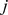 (empirical probability estimate 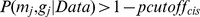) and a significant trans methylation-mRNA relationship between genes 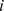 and 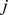 (empirical probability estimate 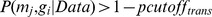), where 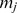 is the methylation level of CpG islands within gene
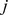
's promoter region, and 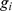 and 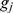 are mRNA expression levels of genes 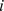 and 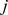, there are multiple causal reactive relationships among 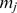, 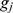, and 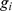 (Fig. 1C). We focused on two possible causal/reactive models: model I (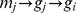), where the methylation level of gene 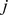 causally regulates *trans* gene expression of gene 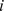 through *cis* regulation on gene
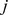
's expression level; and model II (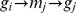), where the expression level of gene 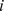 *trans* regulates the methylation level of gene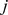. As there are many potential models with hidden regulators [47] we can't enumerate all possible causal reactive models, therefore, we modeled the causality test as an empirical Bayesian estimation of the significance of each causal relationship [46], [47] instead of a model selection problem [45]. As shown by Chen *et al* [46] and Millstein *et al* [47], the probability of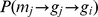 can be decomposed as a product of probabilities of a chain of statistic tests 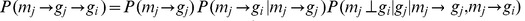. Instead of calculating 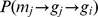 for all possible trios (171,750*15,260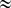 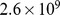), we required each association test (p-values<0.01 and 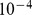 for *cis* and *trans* regulations determined above) to be significant so that only a small fraction of all possible trios were subjected to the causality test.

If assuming that all methylation levels and gene expression levels are normally distributed and that all causal relationships are linear, the probability of
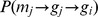
can be estimated analytically. However, the empirical data never perfectly fit to the underlying model assumption. Thus, we applied a permutation approach to estimate a null distribution at each step similar to Chen et al [46]. In all permutation tests, we permuted only the gene expression data. Note that all our tests are non-parametric. The p-values based on permutation tests were similar to the nominal p-values. For example, given a *cis* association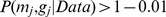, 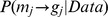
>0.99 based on permutation tests. The two models 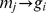 and 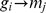
are equally possible given that 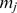 and 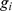 are associated. Given a significant *cis* regulation and a *trans* association 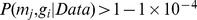 and a non-informative prior of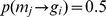, we got 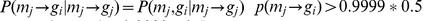. Thus, 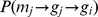
was mainly determined by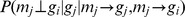.


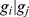
 was calculated as residuals from the linear regression of *trans* gene expression 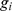
on *cis* gene expression 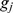. At Spearman correlation p-value>0.01, 42.1% of tested pairs were independent. When checking pairs selected from permuted data sets, only 21.4% of tested pairs were independent. To estimate the FDR of the causality test, 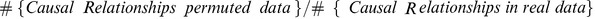, we permuted the whole gene expression data 5 times. At the cutoff values noted above, we identified 362,095 pairs of causal relationships in COPD and 518 pairs in the permuted data on average, with the corresponding FDR 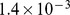. It is possible to set more stringent p-value cutoffs for the conditional independent test 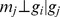. At p-value>0.05 and 0.1, the corresponding FDRs were 0.001 and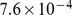, respectively. As at the independent test p-value>0.01, the corresponding FDR for the causality test was far less than 0.05, so that we chose this set of causal relationships for further analyses. Similarly, to test causality in the opposite direction, 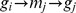, where *trans* gene expression 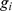
regulates gene 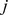
's DNA methylation level, we decomposed 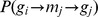 as 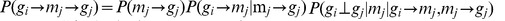. At the same cutoff values noted above, we identified 19,173 pairs of causal relationships 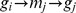 in COPD and 2 pairs in the permuted data, corresponding to an FDR 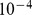. In the CTRL data set, we identified 30,177 causal relationships as 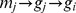 (FDR = 0.03) and 1, 241 causal relationships as 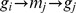 (FDR = 0.006).

A similar causality test 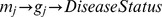 can be applied to infer genes that are potentially causal to COPD (see S1 Text for details).

### Ethics statement

Immunohistochemistry staining of paraffin embedded human lung tissue of de-identified patients was carried out with the IRB approval (HS#12-00171) from Mount Sinai Hospital. Institutional Animal Care and Use Committee (IACUC) approval (FO0501) was obtained for the chronic smoke exposure mouse model systems at St. Lukes Roosevelt Hospital.

### Chronic smoking mouse models

C57Bl/6J and A/J mice (Jackson Labs, Bar Harbor, ME) were exposed to cigarette smoke for 6 or 2 months respectively in a specially designed chamber (Teague Enterprises, Davis, CA) for 4 hours a day, 5 days per week at a total particulate matter concentration of 80 mg/m^3^. Animals were sacrificed 12 hours after the last smoke exposure. Comparative analyses were made with age-matched air-exposed C57Bl/6J and A/J mice that were treated in an identical manner.

### Quantifying *Epas1* and *Vegfa* expression levels in mouse lung tissues by qPCR

The total RNA from mouse lung tissues was extracted using RNeasy Mini Kit (QIAGEN, Germany). Then cDNA was synthesized with SuperScript III (Life Technologies, CA, USA). For quantitative PCR, we utilized TaqMan gene expression assays (Applied Biosystems, Canada), which contain prevalidated primers and TaqMan probe for the individual genes. TaqMan Gene Expression Assay IDs are Mm01236112_m1 and Mm01281449_m1 for mouse *Epas1* and *Vegfa*, respectively. The real-time PCR reactions were carried out following the manufacturer's protocol, and the gene expressions were normalized to *Rn18s* (Mm03928990_g1).

### IHC staining of human lung tissues of COPD and non-COPD patients

The paraffin sections of human lung tissues were provided by Histology Shared Resource Facility of Mount Sinai Hospital with the IRB approval. The immunostaining was performed using Vectastain ABC Elite Kit (Vector Laboratories, CA, USA) with polyclonal anti-EPAS1 antibody (NB10-122; Novus Biologicals, CO, USA). Following deparaffinization and hydration of sections, antigen retrieval with 10 mM citrate buffer and blocking of endogenous peroxidase with 0.3% H2O2-methanol were performed. The tissue sections were blocked with 5% goat serum diluted in 0.1% Tween-20 in phosphate buffered saline (PBS-T) for 30 minutes, and then incubated in anti-EPAS1 (1∶100) at room temperature for 1 hour. The tissue sections were washed and incubated with the secondary antibody anti-rabbit-HRP. After washing, DAB substrate (3, 3′-diaminobenzidine) was utilized to obtain positive reactions.

### *EPAS1* siRNA in human and mouse endothelial cell lines

The cell lines of HUVEC (Lonza, MD, USA) and C166 (American Type Culture Collection, VA, USA) were cultured in the appropriate media at 37°C with 5% CO_2_. The cells were transfected with *EPAS1* siRNA and non-targeting negative control siRNA (Life Technologies, CA, USA) using Lipofectamine RNAiMAX as recommended transfection protocols by the manufacturer. After the treatments with 5 nM Silencer Select siRNA (s4700 for *EPAS1*, s65525 for *Epas1*; Life Technologies, USA) for 48 hours, the total RNA was purified with RNeasy Mini Kit (QIAGEN, Germany). The efficiencies of knocked down the *EPAS1* expression were assessed by qPCR with 1.4% for HUVEC, 3.2% for C166.

Approximately 250 ng of total RNA per sample were used for library construction by the TruSeq RNA Sample Prep Kit (Illumina) and sequenced using the Illumina HiSeq 2500 instrument with 100 nt single read setting according to the manufacturer's instructions. The RNAseq data set was deposited in GEO as GSE62974. Sequence reads were aligned to human genome assembly hg19 and mouse genome assembly mm10, respectively, using Tophat [103]. Total 23,228 human and 22,609 mouse genes were quantified using Cufflinks [103]. siRNA signatures were derived by comparing expression profiles of *EPAS1* or *Epas1* siRNAs with non-targeting siRNAs at paired t-test p-value cutoff 0.05 with resulting signature sizes of 2,796 and 3,730, and corresponding q-values [104] 0.11 and 0.07 for HUVEC and C166, respectively.

## Supporting Information

S1 Fig Comparison of DNA methylation profiles between COPD and CTRL samples. DNA methylation level was measure by β value and the mean of β value of methyl probes of CpG islands within 1 million bases is shown for all chromosomes. Global methylation levels were compared between CTRL (red) and COPD (blue) (the upper panel) and most regions were hypermethylated in COPD comparing with CTRL across whole genomes (the lower panel). A) CpG island probes; B) non-CpG island probes.
(TIF)

S2 Fig The numbers of *cis* and *trans* pairs derived using the same number of samples in CTRL and COPD. A) The numbers of *cis* pairs; B) The numbers of *trans* pairs.
(TIF)

S3 FigClustering results of 67 CTRL key regulators **A**) The clustering result based on the methylation levels of key regulators. The distance was measured as (1-Spearman correlation coefficient). There were two large clusters of key regulators (shown in red boxes). **B**) The clustering result based on topological overlaps of key regulators' downstream genes. The distance was measured as 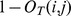. Key regulators were grouped into two clusters similar as shown in S3A Fig.(TIF)

S4 Fig Clustering results of 126 COPD key regulators. **A**) The clustering result based on similarity of key regulators' methylation levels. The distance between methylation levels of COPD key regulators was measured in the same way as in S3 Fig. Key regulators were grouped into three clusters. The key regulators in the blue dashed box were enriched for the GO biological processes metabolic process, RNA processing, cell cycle, chromatin modification. Genes in the cluster in the middle were enriched for genes involved in the GO biological process immune response and T-cell activation. *EPAS1* was not included in any cluster. **B**) The clustering result based on the topological overlaps of COPD key regulators' downstream genes. There were three distinct clusters of COPD key regulators. The first and second cluster, C1 and C2, shared some common downstream genes but key regulators in the C2 cluster regulated genes involved in immune response and other defense processes specifically. The C3 cluster consisted of regulators involved in ciliary related function. *EPAS1* downstream genes were also unique compared to others, and *EPAS1* was not included in the three clusters.
(TIF)

S5 Fig The clustering results of key regulators with higher number of downstream genes>mean+3SD. There were 33 and 60 key regulators for CTRL and COPD set, respectively. Key regulators were clustered based on their methylation profiles or overlaps of their downstream genes. The clustering patterns were similar to the corresponding ones based on key regulators of the number of downstream genes>mean+2SD in S3–S4 Fig. *EPAS1* (marked with the red arrow) was not included in these clusters in COPD.
(TIF)

S6 Fig *EPAS1* methylation and gene expression levels in CTRL and COPD lung tissues. A) *EPAS1* gene expression level was lower in lung tissues of COPD patients; B) Methylation level of *EPAS1* promoter region was higher in lung tissues of COPD patients; C) Methylation and gene expression levels of *EPAS1* were anti-correlated in lung tissues of COPD patients.
(TIF)

S7 Fig An example of immunohistochemistry staining of lung tissues from COPD (A) and non-COPD (B) patients using EPAS1 antibody.
(TIF)

S8 Fig Numbers of downstream genes' methylation levels *trans* regulated a key regulator gene in lung tissues of COPD patients followed a scale-free distribution.
(TIF)

S9 Fig Heterogeneities of molecular traits in CTRL and COPD lung samples. **A**) The clustering result of COPD samples by gene expression levels of 1000 genes with largest variances in COPD. COPD samples can be partitioned into two groups based on expression levels of 306 cilium related genes (marked by a red box in the top-left corner). **B**) The clustering result of CTRL samples based on gene expression levels of 1000 genes with largest variances in CTRL samples. A set of 339 genes classified CTRL samples into two subgroups. 250 of these genes overlap with COPD classifier genes in S9A Fig. **C**) The clustering result of COPD samples based on methylation profiles of 1000 methylation probes with largest variances in COPD. A set of 447 genes (in the red box) classified COPD samples into two groups. **D**) The clustering result of CTRL samples based on methylation profiles of 1000 methylation probes with largest variances in CTRL. A set of 391 genes (in the red box) clustered CTRL samples into two groups. Among them, 95 out of 391 genes overlap with the COPD classifier genes in S9C Fig. For figures, rows are molecular traits (mRNA expression or methylation probes) and columns are samples.
(TIF)

S1 Table Demographic characteristics of samples in LGRC cohort.
(PDF)

S2 Table Characteristics of data used in the analysis.
(PDF)

S3 Table Significance of overlaps between differentially methylated and expressed genes.
(PDF)

S4 Table The distribution of probes differentially methylated between controls and COPD samples.
(PDF)

S5 Table Gene expression levels and DNA methylation levels of 704 genes in S3 Table.
(PDF)

S6 Table GO enrichment analysis of the 378 genes that were hypermethylated and downregulated in COPD.
(PDF)

S7 Table GO enrichment analysis of the 318 genes that were hypermethylated and upregulated in COPD.
(PDF)

S8 Table 67 key regulators in CTRL lung tissues that regulated a large number of downstream genes.
(PDF)

S9 Table 126 key regulators in COPD lung tissues that regulated a large number of downstream genes.
(PDF)

S10 Table Correlation between key regulators' methylation levels in promoter regions in COPD lung tissues and COPD severity Traits.
(PDF)

S11 Table Overlap between downstream genes of key regulators in COPD lung tissues and COPD severity signatures in Human.
(PDF)

S12 Table Overlap between downstream genes of key regulators in COPD lung tissues and COPD emphysema signature in mouse.
(PDF)

S13 Table GO enrichment analysis of *EPAS1* downstream genes in COPD.
(PDF)

S14 Table Matched methylation and gene expression profile pairs identified by MODMatcher.
(PDF)

S15 Table GO enrichment analysis of the 306 genes in the upper left corner of S9A Fig.
(PDF)

S16 Table GO enrichment analysis of *GAK* downstream genes in COPD.
(PDF)

S17 Table GO enrichment analysis of *ETF1* downstream genes in COPD.
(PDF)

S18 Table GO enrichment analysis of *PAX9* downstream genes in COPD.
(PDF)

S19 Table Motif enrichment analysis of downstream genes of key regulator *EPAS1* in COPD.
(PDF)

S1 Text Supplementary results and methods.
(DOCX)
