# Correction: Integrative Analysis of DNA Methylation and Gene Expression Data Identifies *EPAS1* as a Key Regulator of COPD

**DOI:** 10.1371/journal.pgen.1005032

**Published:** 2015-03-05

**Authors:** The PLOS Genetics Staff

The ninth author's name is spelled incorrectly. The correct name is: Robert F. Foronjy.
