# Supplementary figures and images for: Integrative Analysis of DNA Methylation and Gene Expression Data Identifies EPAS1 as a Key Regulator of COPD
Source: PLoS Genet. 2015 Jan 8;11(1):e1004898. doi: 10.1371/journal.pgen.1004898 (PMC4287352; doi:10.1371/journal.pgen.1004898)

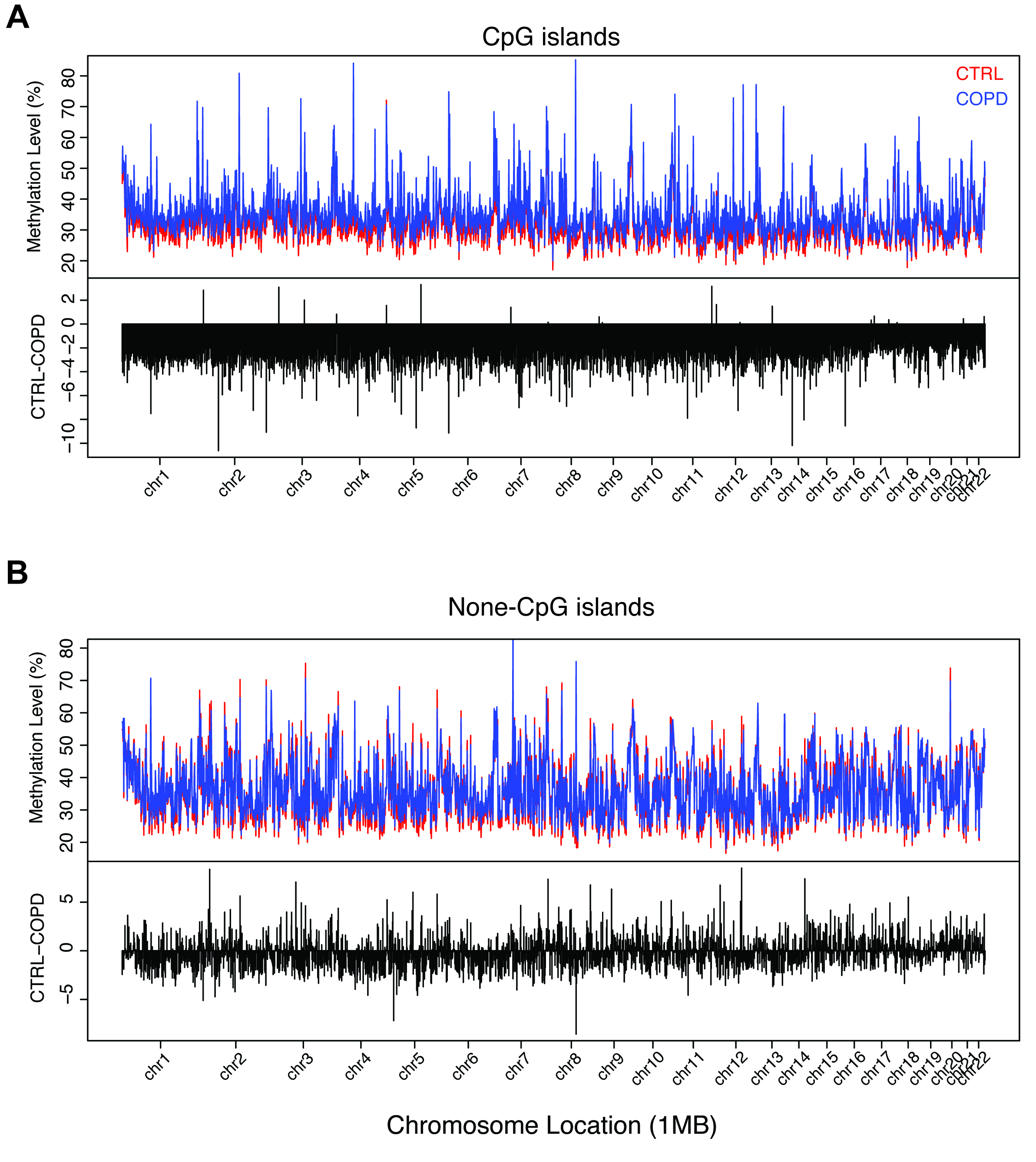

Supplement: S1 Fig — Comparison of DNA methylation profiles between COPD and CTRL samples. DNA methylation level was measure by β value and the mean of β value of methyl probes of CpG islands within 1 million bases is shown for all chromosomes. Global methylation levels were compared between CTRL (red) and COPD (blue) (the upper panel) and most regions were hypermethylated in COPD comparing with CTRL across whole genomes (the lower panel). A) CpG island probes; B) non-CpG island probes. (TIF) [file pgen.1004898.s001.tif]

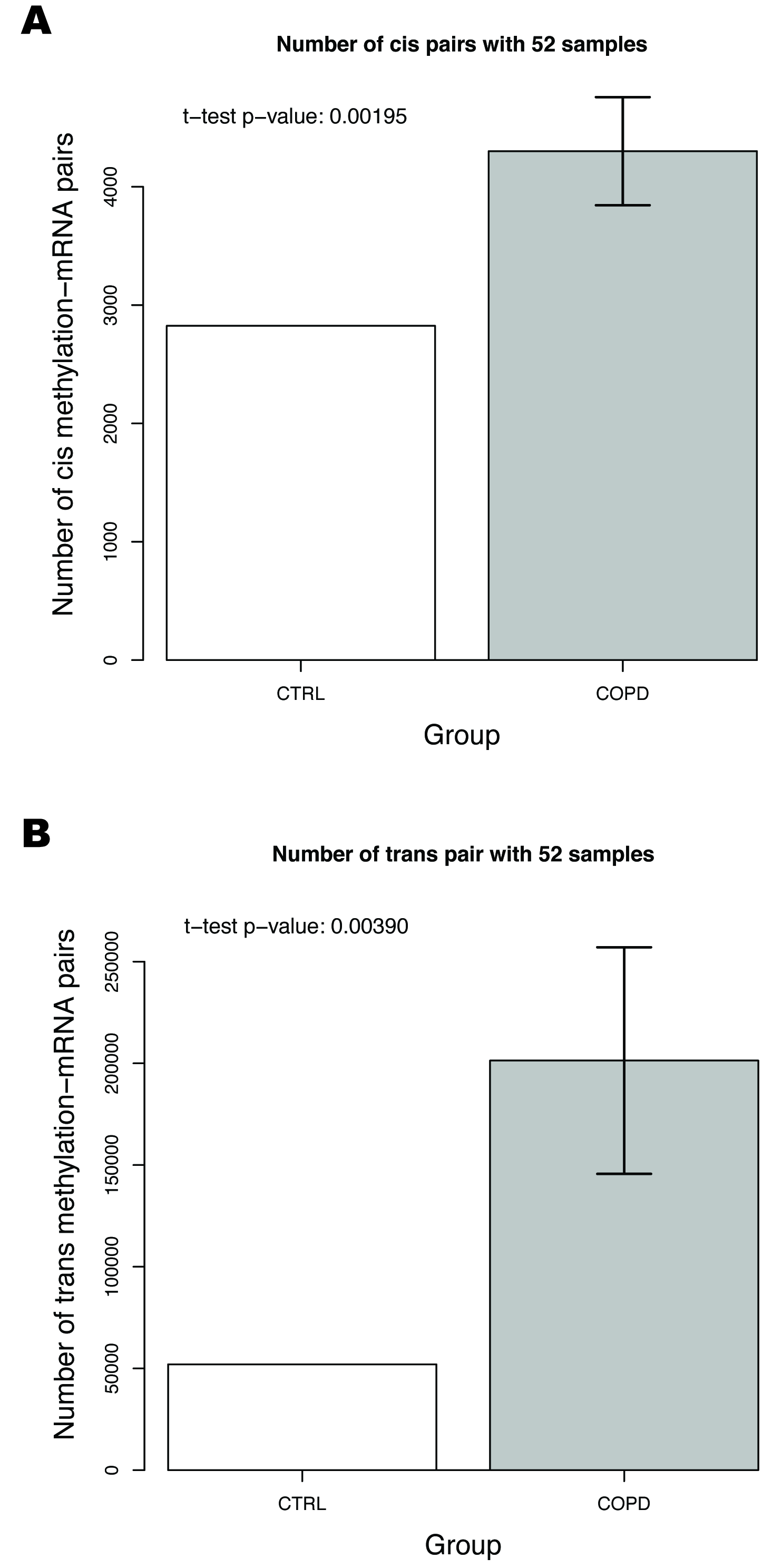

Supplement: S2 Fig — The numbers of cis and trans pairs derived using the same number of samples in CTRL and COPD. A) The numbers of cis pairs; B) The numbers of trans pairs. (TIF) [file pgen.1004898.s002.tif]

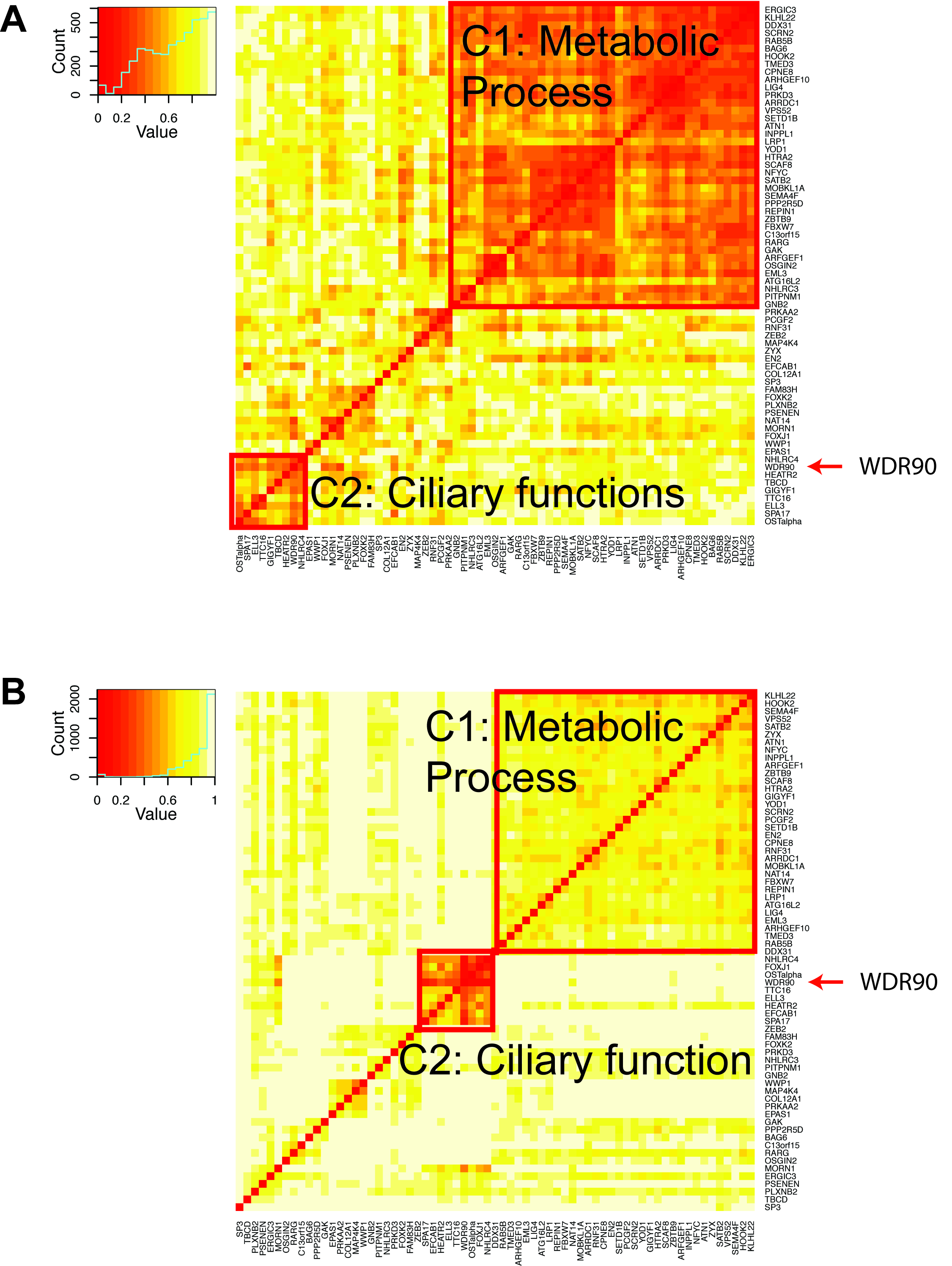

Supplement: S3 Fig — Clustering results of 67 CTRL key regulators A) The clustering result based on the methylation levels of key regulators. The distance was measured as (1-Spearman correlation coefficient). There were two large clusters of key regulators (shown in red boxes). B) The clustering result based on topological overlaps of key regulators' downstream genes. The distance was measured as . Key regulators were grouped into two clusters similar as shown in S3A Fig. (TIF) [file pgen.1004898.s003.tif]

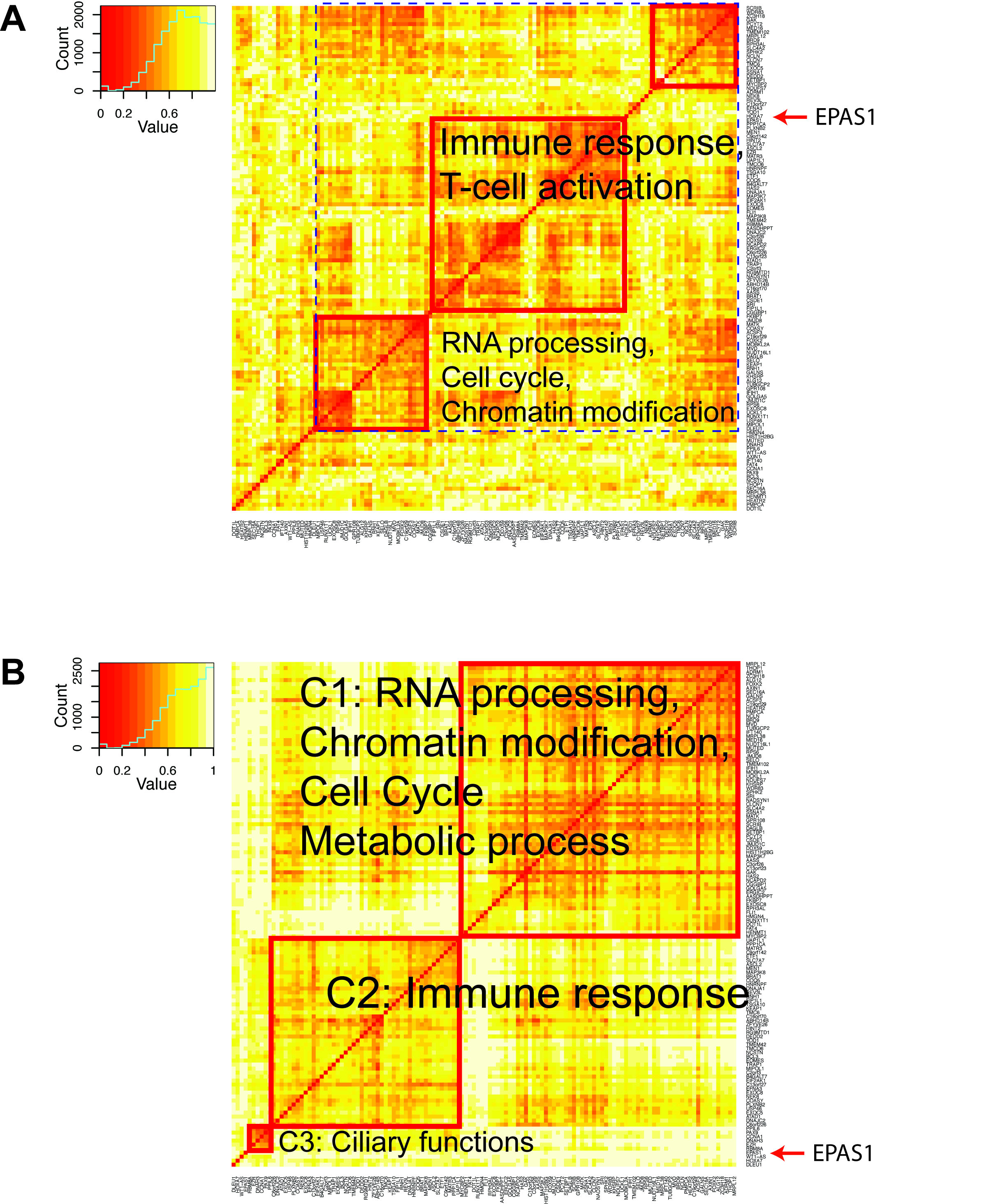

Supplement: S4 Fig — Clustering results of 126 COPD key regulators. A) The clustering result based on similarity of key regulators' methylation levels. The distance between methylation levels of COPD key regulators was measured in the same way as in S3 Fig. Key regulators were grouped into three clusters. The key regulators in the blue dashed box were enriched for the GO biological processes metabolic process, RNA processing, cell cycle, chromatin modification. Genes in the cluster in the middle were enriched for genes involved in the GO biological process immune response and T-cell activation. EPAS1 was not included in any cluster. B) The clustering result based on the topological overlaps of COPD key regulators' downstream genes. There were three distinct clusters of COPD key regulators. The first and second cluster, C1 and C2, shared some common downstream genes but key regulators in the C2 cluster regulated genes involved in immune response and other defense processes specifically. The C3 cluster consisted of regulators involved in ciliary related function. EPAS1 downstream genes were also unique compared to others, and EPAS1 was not included in the three clusters. (TIF) [file pgen.1004898.s004.tif]

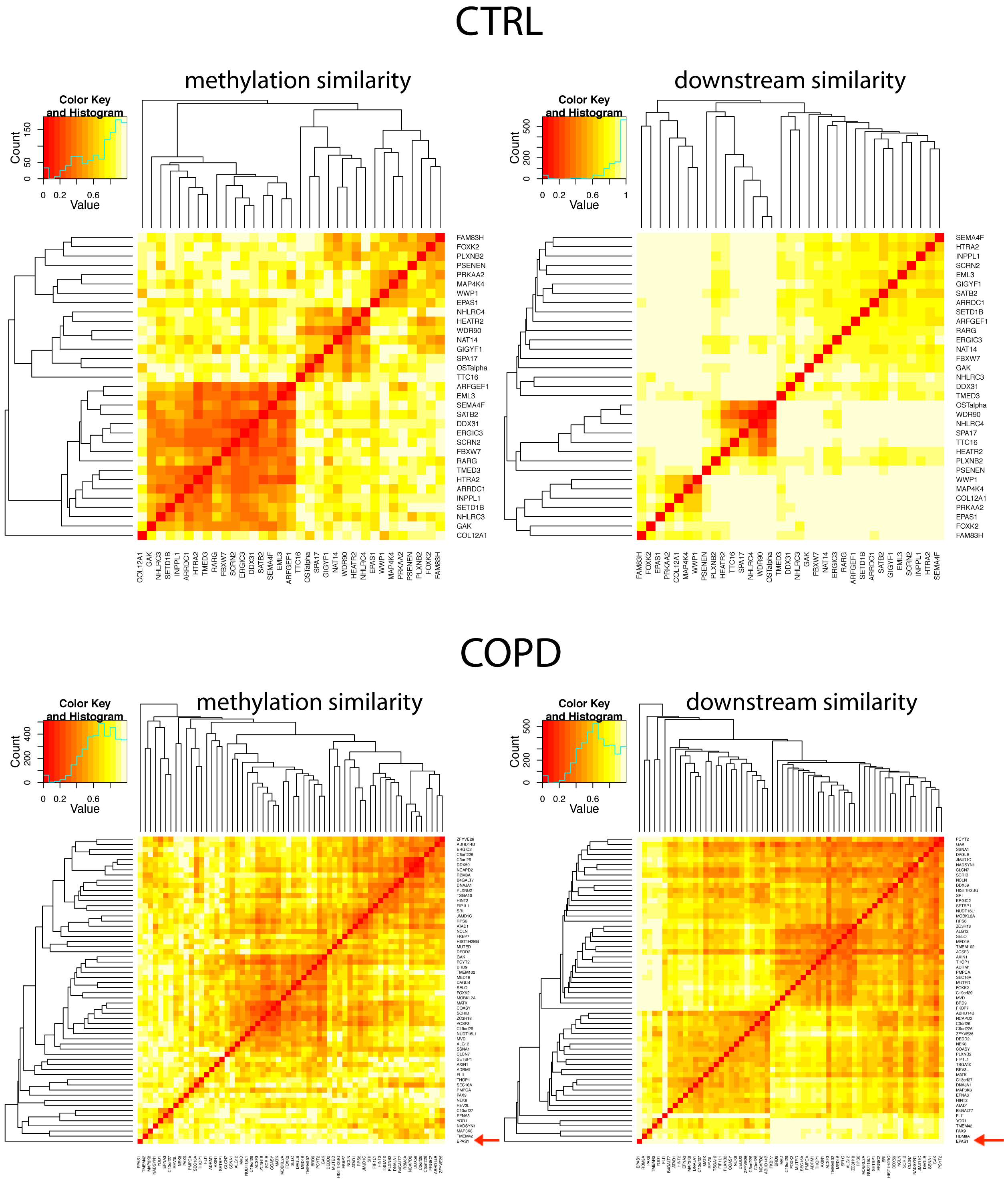

Supplement: S5 Fig — The clustering results of key regulators with higher number of downstream genes>mean+3SD. There were 33 and 60 key regulators for CTRL and COPD set, respectively. Key regulators were clustered based on their methylation profiles or overlaps of their downstream genes. The clustering patterns were similar to the corresponding ones based on key regulators of the number of downstream genes>mean+2SD in S3–S4 Fig. EPAS1 (marked with the red arrow) was not included in these clusters in COPD. (TIF) [file pgen.1004898.s005.tif]

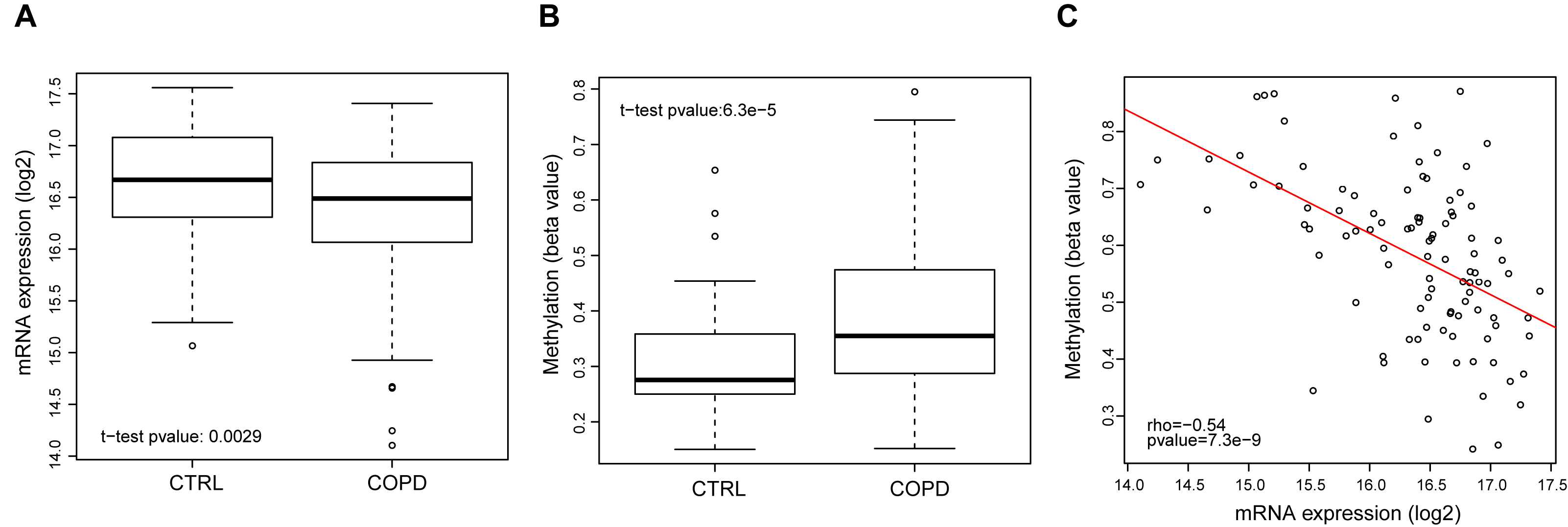

Supplement: S6 Fig — EPAS1 methylation and gene expression levels in CTRL and COPD lung tissues. A) EPAS1 gene expression level was lower in lung tissues of COPD patients; B) Methylation level of EPAS1 promoter region was higher in lung tissues of COPD patients; C) Methylation and gene expression levels of EPAS1 were anti-correlated in lung tissues of COPD patients. (TIF) [file pgen.1004898.s006.tif]

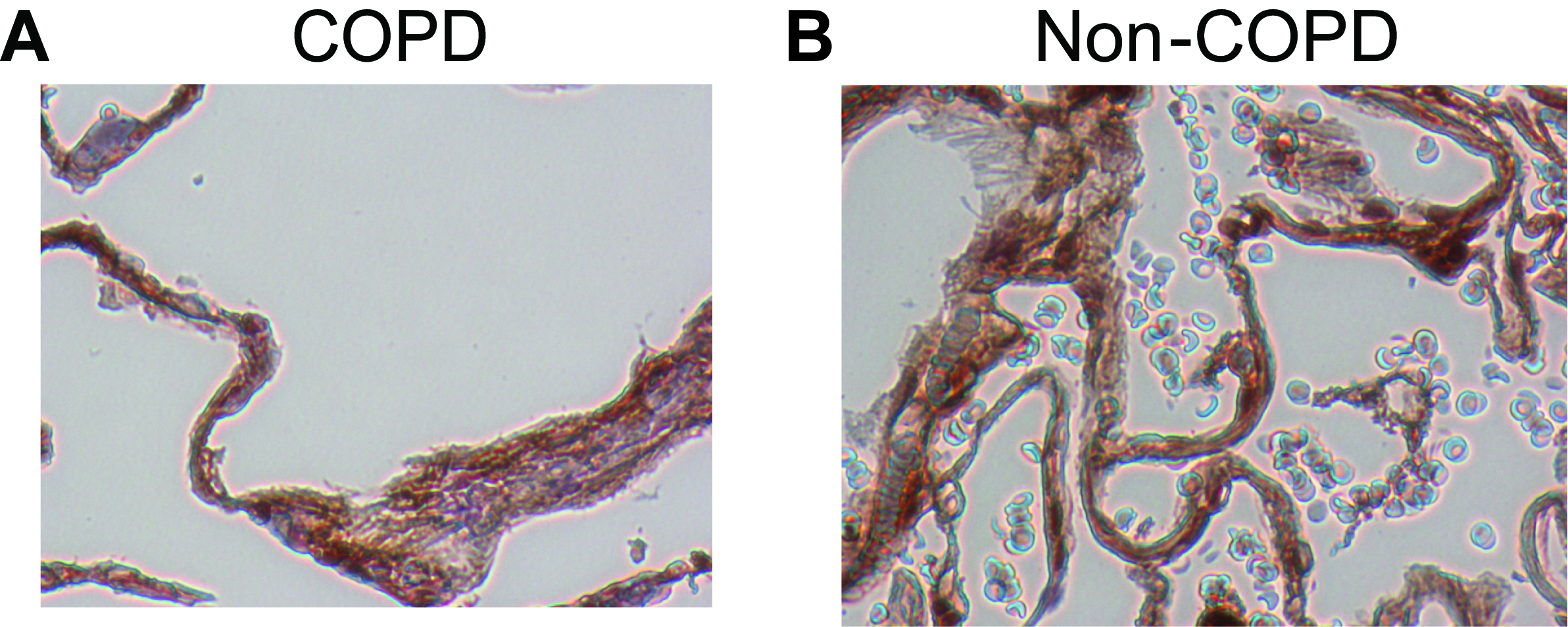

Supplement: S7 Fig — An example of immunohistochemistry staining of lung tissues from COPD (A) and non-COPD (B) patients using EPAS1 antibody. (TIF) [file pgen.1004898.s007.tif]

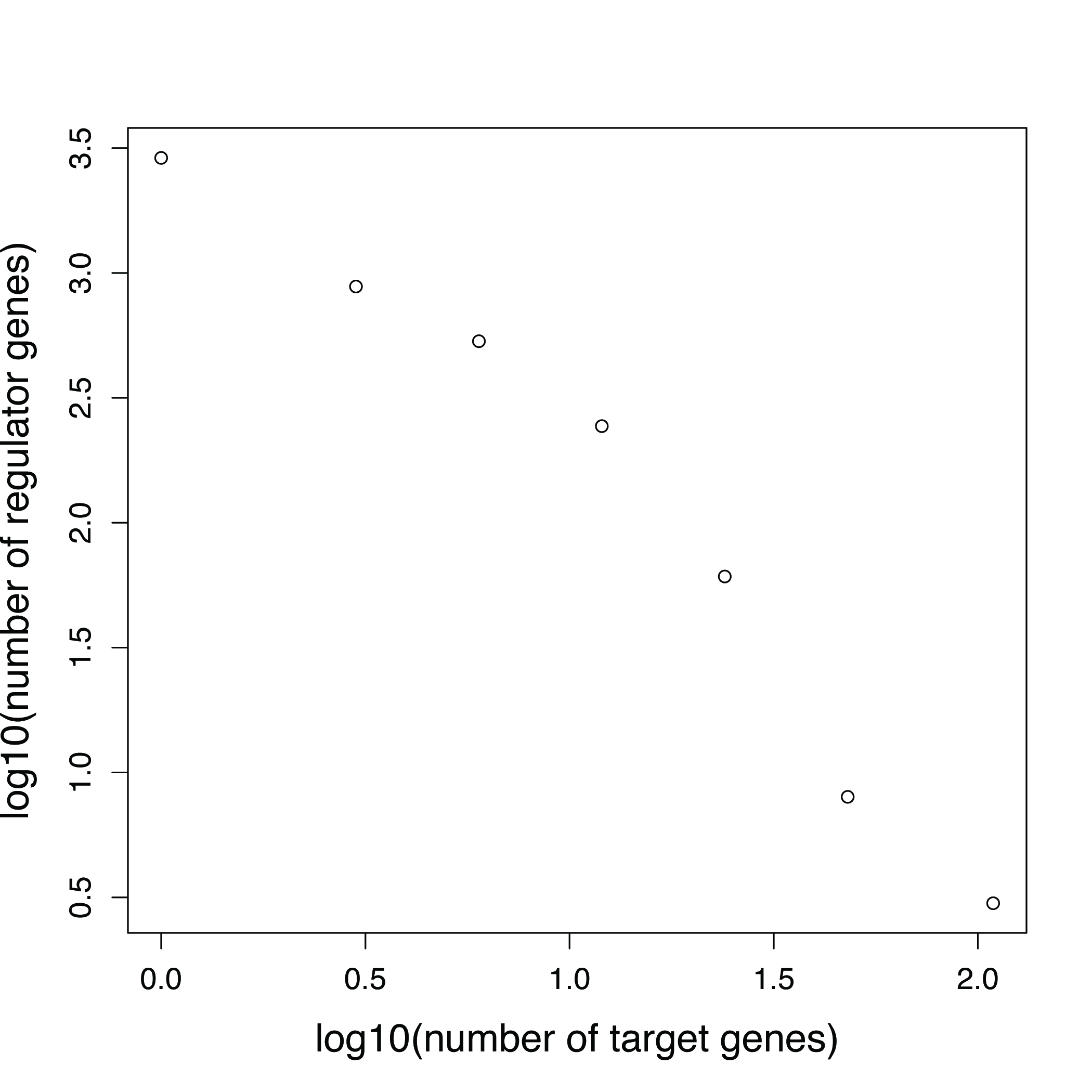

Supplement: S8 Fig — Numbers of downstream genes' methylation levels trans regulated a key regulator gene in lung tissues of COPD patients followed a scale-free distribution. (TIF) [file pgen.1004898.s008.tif]

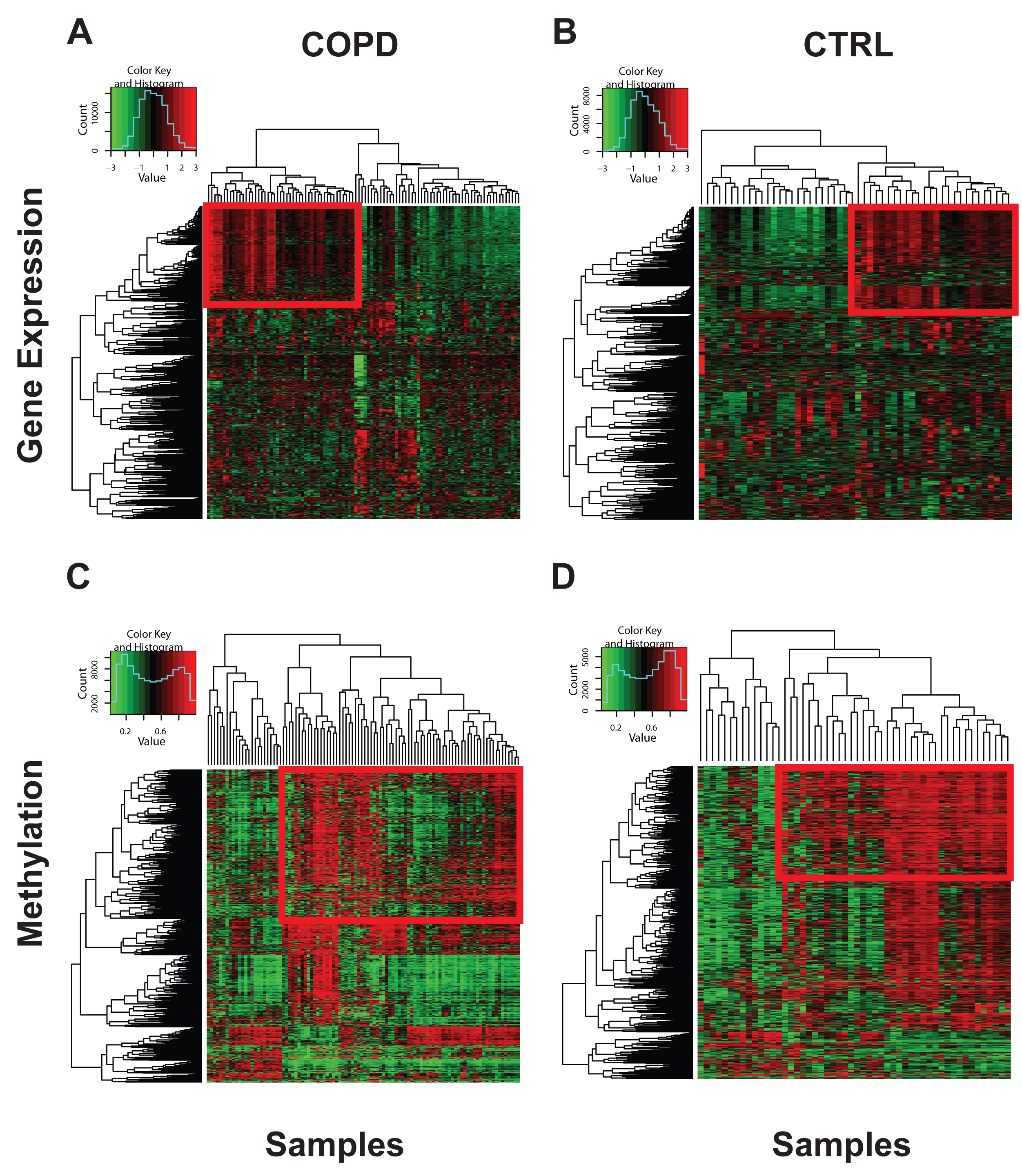

Supplement: S9 Fig — Heterogeneities of molecular traits in CTRL and COPD lung samples. A) The clustering result of COPD samples by gene expression levels of 1000 genes with largest variances in COPD. COPD samples can be partitioned into two groups based on expression levels of 306 cilium related genes (marked by a red box in the top-left corner). B) The clustering result of CTRL samples based on gene expression levels of 1000 genes with largest variances in CTRL samples. A set of 339 genes classified CTRL samples into two subgroups. 250 of these genes overlap with COPD classifier genes in S9A Fig. C) The clustering result of COPD samples based on methylation profiles of 1000 methylation probes with largest variances in COPD. A set of 447 genes (in the red box) classified COPD samples into two groups. D) The clustering result of CTRL samples based on methylation profiles of 1000 methylation probes with largest variances in CTRL. A set of 391 genes (in the red box) clustered CTRL samples into two groups. Among them, 95 out of 391 genes overlap with the COPD classifier genes in S9C Fig. For figures, rows are molecular traits (mRNA expression or methylation probes) and columns are samples. (TIF) [file pgen.1004898.s009.tif]
