# Supplementary material for: Integrative Analysis of DNA Methylation and Gene Expression Data Identifies EPAS1 as a Key Regulator of COPD
Source: PLoS Genet. 2015 Jan 8;11(1):e1004898. doi: 10.1371/journal.pgen.1004898 (PMC4287352; doi:10.1371/journal.pgen.1004898)
Supplement: S1 Table — Demographic characteristics of samples in LGRC cohort. (PDF) [file pgen.1004898.s010.pdf]

STable 1. Demographics information of CTRL and COPD samples

		CTRL	COPD
**Number of samples**		52	100
**Age, years (mean ± SD)**		66 ± 9	64 ± 10
**Gender (%)**	**Male**	44	56
	**Female**	56	44
**Race (%)**	**White**	96	96
	**African-American**	2	4
	**Asian or Pacific Islander**	2	0
**Smoking Status (%)**	**Never Smoker**	32	4
	**Former Smoker**	54	90
	**Current Smoker**	4	5
	**No information**	10	1
**Pack per year (median)**		33.5	44
