# Supplementary material for: Integrative Analysis of DNA Methylation and Gene Expression Data Identifies EPAS1 as a Key Regulator of COPD
Source: PLoS Genet. 2015 Jan 8;11(1):e1004898. doi: 10.1371/journal.pgen.1004898 (PMC4287352; doi:10.1371/journal.pgen.1004898)
Supplement: S2 Table — Characteristics of data used in the analysis. (PDF) [file pgen.1004898.s011.pdf]

STable 2. Characteristics of data used in the analysis			
**Groups**	**CTRL**	**COPD**	**Both**
**Number of Samples**	52	100	N/A
**Number of mRNA probes**	15261	15261	15261
**Number of methyl probes after QC**	1,709,407	1,778,677	1,704,551
**Number of methyl probes within promoter**	660,235	685,788	658,108
**Number of methyl probes within CpG islands**	398,720	421,400	395,443
**Number of methyl probes within CpG islands and promoter regions**	173,276	182,805	171,750
