# Supplementary material for: Integrative Analysis of DNA Methylation and Gene Expression Data Identifies EPAS1 as a Key Regulator of COPD
Source: PLoS Genet. 2015 Jan 8;11(1):e1004898. doi: 10.1371/journal.pgen.1004898 (PMC4287352; doi:10.1371/journal.pgen.1004898)
Supplement: S3 Table — Significance of overlaps between differentially methylated and expressed genes. (PDF) [file pgen.1004898.s012.pdf]

STable 3. Overlaps between differentially methylated and differentially expressed genes between COPD and CTRL lung tissue samples

	No filter	CpG	Multiple probes (>1)	Distance (<1kb)	CpG + Multiple probes	CpG + Distance	Multiple probes + Distance	CpG + Multiple probes + Distance
**Differentially methylated**	8848	6416	8841	7438	6317	5237	7429	5117
**Differentially methylated and expressed**	990	704	990	821	695	549	821	537
**Background*1**	0.85	0.62	0.85	0.72	0.61	0.5	0.72	0.49
**Overlap*2**	0.88	0.68	0.88	0.75	0.72	0.57	0.76	0.61
**p-value*3**	0.009	6.6×10^{-06}	0.001	0.002	5.4×10^{-14}	2.1×10^{-05}	1.3×10^{-04}	2.1×10^{-10}

*1. Background: (Number of differentially methylated genes)/(Number of genes in total = 10375)

*2. Overlap: (Number of differentially methylated and expressed genes)/(Number of differentially expressed genes)

*3. P-value: p-value from Fisher's exact test using genes from background and overlap.
