# Supplementary material for: Integrative Analysis of DNA Methylation and Gene Expression Data Identifies EPAS1 as a Key Regulator of COPD
Source: PLoS Genet. 2015 Jan 8;11(1):e1004898. doi: 10.1371/journal.pgen.1004898 (PMC4287352; doi:10.1371/journal.pgen.1004898)
Supplement: S4 Table — The distribution of probes differentially methylated between controls and COPD samples. (PDF) [file pgen.1004898.s013.pdf]

Stable 4. Numbers of differentially methylated probes within/outside of CpG islands between controls and COPD samples

Chromosome	cpg island methyl probes					nocpg island methyl probes				
	total	t-test p-value<0.01 (FDR<0.054)		BH corrected p-value<0.01 (FDR=0)		total	t-test p-value<0.01 (FDR<0.0590)		BH corrected p-value<0.01 (FDR=0)	
		hypo	hyper	hypo	hyper		hypo	hyper	hypo	hyper
1	13668	43	2425	15	1008	39950	2641	3669	1212	1249
2	11710	46	1991	23	420	28308	2227	2253	1073	662
3	8220	27	1534	8	587	20222	1649	1503	836	419
4	7253	17	1441	6	588	17378	915	1926	459	693
5	8701	67	1491	16	595	20499	1436	1661	660	533
6	9338	31	1650	6	631	20765	1300	1651	603	430
7	9247	45	1873	19	839	25018	1951	2676	939	1069
8	6419	41	1444	18	653	19985	1805	2040	901	857
9	7168	32	1574	6	737	23500	2069	2724	1037	1203
10	7535	40	1628	16	702	18222	1904	1566	947	665
11	9709	29	1968	8	880	36229	3377	3931	1735	1744
12	8180	44	1760	12	726	21296	1529	1895	726	650
13	3583	29	780	13	383	8275	644	752	303	310
14	5838	12	1231	5	530	16247	1327	1595	673	663
15	4538	25	805	12	311	12377	1306	802	657	243
16	9828	70	2326	25	1318	30180	2173	4310	1053	2292
17	11965	67	1915	16	1075	37376	3121	3292	1486	1592
18	2911	32	387	8	167	6743	618	288	284	128
19	15216	86	2941	35	1576	43719	3758	4898	1839	2539
20	4875	38	836	11	420	16389	1371	1422	701	707
21	2277	13	481	4	260	7952	736	992	381	550
22	3571	36	723	9	385	15728	1290	1476	640	726
All	171750	870	33204	291	15125	486358	39147	47322	19145	19924
