# Supplementary material for: Integrative Analysis of DNA Methylation and Gene Expression Data Identifies EPAS1 as a Key Regulator of COPD
Source: PLoS Genet. 2015 Jan 8;11(1):e1004898. doi: 10.1371/journal.pgen.1004898 (PMC4287352; doi:10.1371/journal.pgen.1004898)
Supplement: S5 Table — Gene expression levels and DNA methylation levels of 704 genes in S3 Table. (PDF) [file pgen.1004898.s014.pdf]

STable 5. Gene expression levels and DNA methylation levels of 704 genes in Stable3

		Gene Expression	
		**Downregulated**	**Upregulated**
**Methylation**	**Hypermethylated**	378	318
	**Hypomethylated**	27	27
