# Supplementary material for: Integrative Analysis of DNA Methylation and Gene Expression Data Identifies EPAS1 as a Key Regulator of COPD
Source: PLoS Genet. 2015 Jan 8;11(1):e1004898. doi: 10.1371/journal.pgen.1004898 (PMC4287352; doi:10.1371/journal.pgen.1004898)
Supplement: S6 Table — GO enrichment analysis of the 378 genes that were hypermethylated and downregulated in COPD. (PDF) [file pgen.1004898.s015.pdf]

STable 6. GO enrichment analysis of the 378 genes that were hypermethylated and downregulated in COPD

GOBPID	Pvalue	OddsRatio	ExpCount	Count	Size	Term
GO:0010646	1.98E-08	2.1669741	46.7454662	84	1432	regulation of cell communication
GO:0003008	3.67E-08	2.30307514	34.3082996	67	1051	system process
GO:0023051	3.69E-08	2.1390268	46.5822488	83	1427	regulation of signaling
GO:0009653	5.98E-08	2.09596236	48.6714316	85	1491	anatomical structure morphogenesis
GO:0048646	1.64E-07	2.15499666	38.7151696	71	1186	anatomical structure formation involved in morphogenesis
GO:2000026	2.13E-07	2.36968085	26.3106465	54	806	regulation of multicellular organismal development
GO:0050877	2.82E-07	2.36452542	25.8209942	53	791	neurological system process
GO:0051239	2.99E-07	2.10593559	40.0861959	72	1228	regulation of multicellular organismal process
GO:0048729	4.34E-07	3.00721314	12.1107318	32	371	tissue morphogenesis
GO:0040011	4.96E-07	2.2652549	28.4651163	56	872	locomotion
GO:0002009	5.61E-07	3.2325789	9.85833156	28	302	morphogenesis of an epithelium
GO:0022603	6.07E-07	2.80604648	14.1672712	35	434	regulation of anatomical structure morphogenesis
GO:0007399	9.74E-07	2.00456189	43.7096224	75	1339	nervous system development
GO:0010648	1.04E-06	2.61735315	16.4523149	38	504	negative regulation of cell communication
GO:0048585	1.39E-06	2.51813487	17.9865586	40	551	negative regulation of response to stimulus
GO:0022612	1.41E-06	6.05160823	2.57883508	13	79	gland morphogenesis
GO:0009966	1.58E-06	2.00595458	41.0655003	71	1258	regulation of signal transduction
GO:0000902	1.94E-06	2.28766282	23.8297418	48	730	cell morphogenesis
GO:0023057	2.28E-06	2.55634419	16.321741	37	500	negative regulation of signaling
GO:0050793	2.68E-06	2.06665829	33.8186473	61	1036	regulation of developmental process
GO:0000904	2.72E-06	2.44203665	18.4762108	40	566	cell morphogenesis involved in differentiation
GO:0032989	2.92E-06	2.23079271	24.9069767	49	763	cellular component morphogenesis
GO:0008015	3.76E-06	3.51861913	6.75720077	21	207	blood circulation
GO:0022008	3.97E-06	2.11031606	29.6402816	55	908	neurogenesis
GO:0003013	4.06E-06	3.49940895	6.78984425	21	208	circulatory system process

GO:0048667	5.68E-06	2.54834906	14.9507147	34	458	cell morphogenesis involved in neuron differentiation
GO:0048812	6.54E-06	2.52956674	15.0486452	34	461	neuron projection morphogenesis
GO:0072358	7.01E-06	2.36252979	18.5088543	39	567	cardiovascular system development
GO:0072359	7.01E-06	2.36252979	18.5088543	39	567	circulatory system development
GO:0048699	7.06E-06	2.10428174	27.9428206	52	856	generation of neurons
GO:0048666	7.11E-06	2.28605547	20.6306806	42	632	neuron development
GO:0030182	7.20E-06	2.14887612	25.7230638	49	788	neuron differentiation
GO:0030030	8.18E-06	2.18935784	23.6338809	46	724	cell projection organization
GO:0072657	8.51E-06	5.48339933	2.57883508	12	79	protein localization to membrane
GO:0048858	1.00E-05	2.37711356	17.3989759	37	533	cell projection morphogenesis
GO:0044057	1.02E-05	2.92133638	9.56454022	25	293	regulation of system process
GO:0007435	1.07E-05	11.7707172	0.81608705	7	25	salivary gland morphogenesis
GO:0048468	1.16E-05	1.92963487	37.7685086	64	1157	cell development
GO:0031175	1.17E-05	2.33082664	18.2150629	38	558	neuron projection development
GO:0006873	1.22E-05	2.60659959	12.8288884	30	393	cellular ion homeostasis
GO:0035023	1.28E-05	4.1977507	4.08043525	15	125	regulation of Rho protein signal transduction
GO:0009887	1.36E-05	2.26309136	19.7493066	40	605	organ morphogenesis
GO:0032990	1.40E-05	2.33719832	17.6601237	37	541	cell part morphogenesis
GO:0001944	1.48E-05	2.62334537	12.3065927	29	377	vasculature development
GO:0007409	1.54E-05	2.52864459	13.6449755	31	418	axonogenesis
GO:0001568	1.54E-05	2.66692828	11.6863665	28	358	blood vessel development
GO:0050801	1.58E-05	2.48526695	14.3304886	32	439	ion homeostasis
GO:0032879	1.60E-05	2.00898987	30.2931513	54	928	regulation of localization
GO:0006935	2.28E-05	2.5127197	13.2532537	30	406	chemotaxis
GO:0042330	2.28E-05	2.5127197	13.2532537	30	406	taxis
GO:0007431	2.42E-05	10.0858417	0.9140175	7	28	salivary gland development
GO:0060429	2.56E-05	2.45564185	14.0040538	31	429	epithelium development
GO:0040012	2.99E-05	2.72204794	10.1847664	25	312	regulation of locomotion

GO:0007009	3.23E-05	5.16469361	2.48090463	11	76	plasma membrane organization
GO:0048732	3.44E-05	3.19640963	6.62662684	19	203	gland development
GO:0065009	3.61E-05	1.82577572	41.587796	67	1274	regulation of molecular function
GO:0048514	3.70E-05	2.68340854	10.3153403	25	316	blood vessel morphogenesis
GO:0009888	4.06E-05	1.96678614	28.987412	51	888	tissue development
GO:0009605	5.08E-05	2.01479613	25.396629	46	778	response to external stimulus
GO:0035637	5.48E-05	2.2007722	18.084489	36	554	multicellular organismal signaling
GO:0003018	5.92E-05	4.79312349	2.64412204	11	81	vascular process in circulatory system
GO:0019226	7.10E-05	2.19454695	17.5948368	35	539	transmission of nerve impulse
GO:0055082	7.27E-05	2.34317543	14.1019842	30	432	cellular chemical homeostasis
GO:0001525	7.48E-05	2.81881978	8.22615746	21	252	angiogenesis
GO:0007411	8.14E-05	2.65841292	9.53189674	23	292	axon guidance
GO:0009968	8.69E-05	2.25242084	15.6362279	32	479	negative regulation of signal transduction
GO:0007267	9.13E-05	2.00404687	23.7318114	43	727	cell-cell signaling
GO:0045785	9.27E-05	4.53202016	2.77469597	11	85	positive regulation of cell adhesion
GO:0035272	0.00011286	7.55852843	1.14252187	7	35	exocrine system development
GO:0007204	0.00011487	4.4117752	2.83998293	11	87	elevation of cytosolic calcium ion concentration
GO:0007266	0.00012753	4.35399516	2.87262641	11	88	Rho protein signal transduction
GO:0048878	0.00013187	2.1187257	18.149776	35	556	chemical homeostasis
GO:2000021	0.00014136	4.29769665	2.9052699	11	89	regulation of ion homeostasis
GO:0060485	0.00014744	3.48674685	4.47215703	14	137	mesenchyme development
GO:0016477	0.00014936	2.08107448	18.9985065	36	582	cell migration
GO:0061061	0.00015278	2.43216192	11.2620013	25	345	muscle structure development
GO:0045216	0.0001531	3.68095058	3.94986132	13	121	cell-cell junction organization
GO:0035295	0.00015987	2.42430793	11.2946448	25	346	tube development
GO:0060445	0.00016064	12.5359911	0.55493919	5	17	branching involved in salivary gland morphogenesis
GO:0016044	0.00016457	2.5228808	9.98890548	23	306	cellular membrane organization
GO:0031644	0.00018569	3.40258752	4.57008748	14	140	regulation of neurological system process
GO:0061138	0.00018569	3.40258752	4.57008748	14	140	morphogenesis of a branching epithelium

GO:0051480	0.00019076	4.13714166	3.00320034	11	92	cytosolic calcium ion homeostasis
GO:0061024	0.00019928	2.48658598	10.1194794	23	310	membrane organization
GO:0034332	0.00021225	4.96584022	2.08918285	9	64	adherens junction organization
GO:0007507	0.00021895	2.46881534	10.1847664	23	312	heart development
GO:0044092	0.00023068	2.17820034	15.0486452	30	461	negative regulation of molecular function
GO:0001738	0.00023639	8.22363636	0.9140175	6	28	morphogenesis of a polarized epithelium
GO:0055065	0.00023944	2.90457516	6.43076595	17	197	metal ion homeostasis
GO:0007186	0.00025704	2.38843426	10.9682099	24	336	G-protein coupled receptor signaling pathway
GO:0055074	0.00026853	3.2708878	4.73330489	14	145	calcium ion homeostasis
GO:0010719	0.00030638	17.1447493	0.3590783	4	11	negative regulation of epithelial to mesenchymal transition
GO:0014706	0.00031825	2.65018052	7.83443567	19	240	striated muscle tissue development
GO:0043410	0.00034153	2.71568627	7.246853	18	222	positive regulation of MAPK cascade
GO:0050878	0.00034841	2.29085966	11.8822274	25	364	regulation of body fluid levels
GO:0048870	0.00034846	1.96223699	20.5980371	37	631	cell motility
GO:0051674	0.00034846	1.96223699	20.5980371	37	631	localization of cell
GO:0001763	0.00035598	3.172552	4.86387881	14	149	morphogenesis of a branching structure
GO:2000027	0.00035908	3.55260551	3.75400043	12	115	regulation of organ morphogenesis
GO:0042692	0.00036072	2.70213415	7.27949648	18	223	muscle cell differentiation
GO:0050805	0.00037131	10.0254707	0.65286964	5	20	negative regulation of synaptic transmission
GO:0006928	0.00037289	1.83715983	26.8655857	45	823	cellular component movement
GO:0007167	0.00040161	1.92867787	21.5120546	38	659	enzyme linked receptor protein signaling pathway
GO:0003007	0.00040817	3.12553745	4.92916578	14	151	heart morphogenesis
GO:0030168	0.00042219	3.28070967	4.37422658	13	134	platelet activation
GO:0051960	0.00042746	2.25626914	12.0454448	25	369	regulation of nervous system development
GO:0030003	0.00045337	2.73390822	6.78984425	17	208	cellular cation homeostasis
GO:0042391	0.00045337	2.73390822	6.78984425	17	208	regulation of membrane potential
GO:2000145	0.0004749	2.42881924	9.40132281	21	288	regulation of cell motility
GO:0030334	0.00048073	2.4870291	8.74845317	20	268	regulation of cell migration

GO:0072659	0.00048265	4.94124093	1.86067847	8	57	protein localization to plasma membrane
GO:0050817	0.00048484	2.37009864	10.0868359	22	309	coagulation
GO:0030324	0.00049195	3.41827198	3.88457435	12	119	lung development
GO:0060284	0.0004968	2.15913426	13.5796885	27	416	regulation of cell development
GO:0055080	0.00050458	2.54388729	8.12822701	19	249	cation homeostasis
GO:0060537	0.00050458	2.54388729	8.12822701	19	249	muscle tissue development
GO:1901342	0.00051499	3.63802506	3.36227864	11	103	regulation of vasculature development
GO:0019725	0.0005446	2.05757286	15.8320888	30	485	cellular homeostasis
GO:0022604	0.00055101	2.59829812	7.54064434	18	231	regulation of cell morphogenesis
GO:0007155	0.00057534	1.94000756	19.6187327	35	601	cell adhesion
GO:0022610	0.000593	1.93635825	19.6513761	35	602	biological adhesion
GO:0051970	0.00059774	8.84404925	0.7181566	5	22	negative regulation of transmission of nerve impulse
GO:0072507	0.00060566	2.99238433	5.12502667	14	157	divalent inorganic cation homeostasis
GO:0050808	0.0006072	3.5598269	3.42756561	11	105	synapse organization
GO:0030323	0.00061689	3.32393321	3.9825048	12	122	respiratory tube development
GO:0035282	0.00064039	4.1972028	2.41561767	9	74	segmentation
GO:0042060	0.00065897	2.18359982	12.4045231	25	380	wound healing
GO:0034330	0.00068835	3.09886945	4.60273096	13	141	cell junction organization
GO:0006875	0.00070829	2.81607741	5.81053979	15	178	cellular metal ion homeostasis
GO:0044087	0.00070887	2.46679604	8.35673138	19	256	regulation of cellular component biogenesis
GO:0014031	0.00071279	3.48488701	3.49285257	11	107	mesenchymal cell development
GO:0051056	0.00074321	2.45615319	8.38937487	19	257	regulation of small GTPase mediated signal transduction
GO:0007517	0.00077897	2.44559941	8.42201835	19	258	muscle organ development
GO:0031623	0.00084041	6.2337931	1.14252187	6	35	receptor internalization
GO:0042310	0.00084041	6.2337931	1.14252187	6	35	vasoconstriction
GO:0051270	0.00087855	2.25625848	10.5438447	22	323	regulation of cellular component movement
GO:0045595	0.00093032	1.79385744	24.8416898	41	761	regulation of cell differentiation
GO:0007156	0.00094679	3.61325611	3.06848731	10	94	homophilic cell adhesion

GO:0050807	0.00098125	6.02533333	1.17516535	6	36	regulation of synapse organization
GO:0051145	0.00098125	6.02533333	1.17516535	6	36	smooth muscle cell differentiation
GO:0007596	0.00104206	2.27076639	9.98890548	21	306	blood coagulation
GO:0060021	0.00106121	4.32023011	2.08918285	8	64	palate development
GO:0031645	0.00111289	7.51495017	0.81608705	5	25	negative regulation of neurological system process
GO:0042551	0.00111289	7.51495017	0.81608705	5	25	neuron maturation
GO:0043408	0.00116298	2.20321932	10.7723491	22	330	regulation of MAPK cascade
GO:0007599	0.00117868	2.24634503	10.0868359	21	309	hemostasis
GO:0030855	0.0012404	2.65031881	6.13697461	15	188	epithelial cell differentiation
GO:0051049	0.00126203	1.81436492	22.0669938	37	676	regulation of transport
GO:0010721	0.00130789	3.44748157	3.19906123	10	98	negative regulation of cell development
GO:0007200	0.0013167	5.6475	1.24045231	6	38	phospholipase C-activating G-protein coupled receptor signaling pathway
GO:0051969	0.00132553	3.01804689	4.3415831	12	133	regulation of transmission of nerve impulse
GO:0042592	0.00133716	1.74412329	26.1147856	42	800	homeostatic process
GO:0017145	0.00134152	7.15630438	0.84873053	5	26	stem cell division
GO:0030155	0.00145416	2.60426429	6.23490506	15	191	regulation of cell adhesion
GO:0002090	0.00148469	9.99558499	0.52229571	4	16	regulation of receptor internalization
GO:0010771	0.00148469	9.99558499	0.52229571	4	16	negative regulation of cell morphogenesis involved in differentiation
GO:0048762	0.0014962	3.15260633	3.81928739	11	117	mesenchymal cell differentiation
GO:0042127	0.00150812	1.73165138	26.278003	42	805	regulation of cell proliferation
GO:0051093	0.00153111	2.07664342	12.4371666	24	381	negative regulation of developmental process
GO:0003015	0.0016051	3.63353535	2.74205249	9	84	heart process
GO:0060047	0.0016051	3.63353535	2.74205249	9	84	heart contraction
GO:0006874	0.00160689	2.94404213	4.43951355	12	136	cellular calcium ion homeostasis
GO:0060541	0.00160689	2.94404213	4.43951355	12	136	respiratory system development
GO:0009611	0.00164435	1.82931034	20.0430979	34	614	response to wounding
GO:0032844	0.00165164	2.66934932	5.67996586	14	174	regulation of homeostatic process

GO:0010038	0.00171095	2.92016327	4.47215703	12	137	response to metal ion
GO:0051346	0.00171095	2.92016327	4.47215703	12	137	negative regulation of hydrolase activity
GO:0010811	0.00173334	5.31411765	1.30573928	6	40	positive regulation of cell-substrate adhesion
GO:0050803	0.00173334	5.31411765	1.30573928	6	40	regulation of synapse structure and activity
GO:0050880	0.00174162	3.96391242	2.25240026	8	69	regulation of blood vessel size
GO:0090090	0.00174162	3.96391242	2.25240026	8	69	negative regulation of canonical Wnt receptor signaling pathway
GO:0051100	0.00174956	4.49348893	1.76274803	7	54	negative regulation of binding
GO:0040013	0.00177406	3.29612221	3.32963516	10	102	negative regulation of locomotion
GO:0010035	0.00178653	2.54524628	6.36547898	15	195	response to inorganic substance
GO:0010769	0.00185612	2.74962078	5.12502667	13	157	regulation of cell morphogenesis involved in differentiation
GO:0046903	0.00188441	1.90665728	16.3543845	29	501	secretion
GO:0001954	0.00189208	9.22567499	0.55493919	4	17	positive regulation of cell-matrix adhesion
GO:0048103	0.00189208	9.22567499	0.55493919	4	17	somatic stem cell division
GO:0055078	0.00189208	9.22567499	0.55493919	4	17	sodium ion homeostasis
GO:0048286	0.00190011	6.53257258	0.9140175	5	28	lung alveolus development
GO:0035150	0.00191185	3.89954536	2.28504374	8	70	regulation of tube size
GO:0043112	0.00191185	3.89954536	2.28504374	8	70	receptor metabolic process
GO:0031589	0.00196467	2.73035189	5.15767015	13	158	cell-substrate adhesion
GO:0001701	0.00204274	2.28915975	8.45466183	18	259	in utero embryonic development
GO:0046578	0.00207555	2.50267591	6.46340943	15	198	regulation of Ras protein signal transduction
GO:0007519	0.00218421	2.82834994	4.60273096	12	141	skeletal muscle tissue development
GO:0060711	0.00223555	6.25968992	0.94666098	5	29	labyrinthine layer development
GO:0010959	0.0022363	2.98171913	4.01514828	11	123	regulation of metal ion transport
GO:0007588	0.00224305	5.01777778	1.37102624	6	42	excretion
GO:0043086	0.00228184	2.07729197	11.3599317	22	348	negative regulation of catalytic activity
GO:0048568	0.00229793	2.20773942	9.2381054	19	283	embryonic organ development
GO:0043113	0.00237081	8.56575213	0.58758268	4	18	receptor clustering

GO:0060317	0.00237081	8.56575213	0.58758268	4	18	cardiac epithelial to mesenchymal transition
GO:0001570	0.00240326	4.22247492	1.86067847	7	57	vasculogenesis
GO:0051129	0.00241142	2.31189542	7.89972264	17	242	negative regulation of cellular component organization
GO:0016337	0.00241565	2.25076531	8.58523576	18	263	cell-cell adhesion
GO:0007169	0.00242439	1.91595146	15.1139322	27	463	transmembrane receptor protein tyrosine kinase signaling pathway
GO:0001666	0.0024526	2.65586275	5.28824408	13	162	response to hypoxia
GO:0035265	0.00250235	3.71832731	2.38297418	8	73	organ growth
GO:0043405	0.00251513	2.53995434	5.94111372	14	182	regulation of MAP kinase activity
GO:0009790	0.00253246	1.72163098	23.7644549	38	728	embryo development
GO:0060828	0.00253687	3.12447757	3.49285257	10	107	regulation of canonical Wnt receptor signaling pathway
GO:0036293	0.00258898	2.63786121	5.32088756	13	163	response to decreased oxygen levels
GO:0000165	0.00259799	2.01837254	12.2086623	23	374	MAPK cascade
GO:0051094	0.00259885	1.86085013	16.7134628	29	512	positive regulation of developmental process
GO:0007528	0.00261213	6.00863787	0.97930446	5	30	neuromuscular junction development
GO:0048259	0.00261213	6.00863787	0.97930446	5	30	regulation of receptor-mediated endocytosis
GO:0048566	0.00261213	6.00863787	0.97930446	5	30	embryonic digestive tract development
GO:0051928	0.00261213	6.00863787	0.97930446	5	30	positive regulation of calcium ion transport
GO:0035239	0.00262817	2.29100803	7.9650096	17	244	tube morphogenesis
GO:0061053	0.00265842	4.13922224	1.89332195	7	58	somite development
GO:0043393	0.00272805	3.66158227	2.41561767	8	74	regulation of protein binding
GO:0050767	0.00273508	2.08169779	10.8049925	21	331	regulation of neurogenesis
GO:0030879	0.00280209	3.32076866	2.97055686	9	91	mammary gland development
GO:0048469	0.00280209	3.32076866	2.97055686	9	91	cell maturation
GO:0060538	0.00292115	2.7212915	4.76594837	12	146	skeletal muscle organ development
GO:0007416	0.00293388	4.0591716	1.92596544	7	59	synapse assembly
GO:0043270	0.00296933	3.6065311	2.44826115	8	75	positive regulation of ion transport

GO:0044093	0.00300501	1.67997174	25.6251333	40	785	positive regulation of molecular function
GO:0072503	0.00309033	2.70083144	4.79859185	12	147	cellular divalent inorganic cation homeostasis
GO:0060402	0.00320937	4.63025641	1.46895669	6	45	calcium ion transport into cytosol
GO:0022602	0.00323068	3.98214173	1.95860892	7	60	ovulation cycle process
GO:0032412	0.00323068	3.98214173	1.95860892	7	60	regulation of ion transmembrane transporter activity
GO:0007187	0.00324943	3.24098124	3.03584382	9	93	G-protein coupled receptor signaling pathway, coupled to cyclic nucleotide second messenger
GO:0030178	0.00324943	3.24098124	3.03584382	9	93	negative regulation of Wnt receptor signaling pathway
GO:0035556	0.00326948	1.53114356	42.6323875	60	1306	intracellular signal transduction
GO:0051128	0.00336246	1.63001884	29.0853424	44	891	regulation of cellular component organization
GO:0008283	0.00342502	1.58289408	34.1450821	50	1046	cell proliferation
GO:0000098	0.00348473	12.8161245	0.32643482	3	10	sulfur amino acid catabolic process
GO:0051930	0.00348473	12.8161245	0.32643482	3	10	regulation of sensory perception of pain
GO:0051931	0.00348473	12.8161245	0.32643482	3	10	regulation of sensory perception
GO:0045765	0.00349324	3.20249554	3.06848731	9	94	regulation of angiogenesis
GO:0035567	0.00349992	5.56232312	1.04459142	5	32	non-canonical Wnt receptor signaling pathway
GO:0034765	0.00350156	3.50121584	2.51354811	8	77	regulation of ion transmembrane transport
GO:0007265	0.00354858	2.43643836	6.16961809	14	189	Ras protein signal transduction
GO:0061098	0.0035656	7.49337748	0.65286964	4	20	positive regulation of protein tyrosine kinase activity
GO:0045892	0.00356902	1.73319672	21.0224024	34	644	negative regulation of transcription, DNA-dependent
GO:0060401	0.00359169	4.514	1.50160017	6	46	cytosolic calcium ion transport
GO:0042698	0.00389252	3.83648525	2.02389588	7	62	ovulation cycle
GO:0070482	0.0039193	2.5020521	5.58203542	13	171	response to oxygen levels
GO:0009880	0.00400684	4.40341463	1.53424365	6	47	embryonic pattern specification
GO:0051336	0.00402523	1.81802793	16.4523149	28	504	regulation of hydrolase activity
GO:0052547	0.00405942	2.60291545	4.96180926	12	152	regulation of peptidase activity
GO:0050790	0.00405989	1.58051869	32.7414124	48	1003	regulation of catalytic activity
GO:0051098	0.00410891	2.73425952	4.3415831	11	133	regulation of binding

GO:0045995	0.0042597	3.76755853	2.05653936	7	63	regulation of embryonic development
GO:0090066	0.00428015	2.38092141	6.30019202	14	193	regulation of anatomical structure size
GO:0050730	0.00431179	3.0922865	3.16641775	9	97	regulation of peptidyl-tyrosine phosphorylation
GO:0010720	0.00434989	2.71172661	4.37422658	11	134	positive regulation of cell development
GO:0043406	0.00434989	2.71172661	4.37422658	11	134	positive regulation of MAP kinase activity
GO:0060562	0.00435274	2.22931822	7.67121826	16	235	epithelial tube morphogenesis
GO:0048041	0.00458587	5.17756902	1.10987839	5	34	focal adhesion assembly
GO:0001892	0.00465249	3.70105028	2.08918285	7	64	embryonic placenta development
GO:0030857	0.00467661	11.2128713	0.3590783	3	11	negative regulation of epithelial cell differentiation
GO:0060347	0.00467661	11.2128713	0.3590783	3	11	heart trabecula formation
GO:0048608	0.00490559	2.34088514	6.39812247	14	196	reproductive structure development
GO:0061458	0.00490559	2.34088514	6.39812247	14	196	reproductive system development
GO:0001756	0.00494223	4.19767442	1.59953062	6	49	somitogenesis
GO:0022898	0.005072	3.63683543	2.12182633	7	65	regulation of transmembrane transporter activity
GO:0044273	0.00511409	6.65930831	0.7181566	4	22	sulfur compound catabolic process
GO:0060441	0.00511409	6.65930831	0.7181566	4	22	epithelial tube branching involved in lung morphogenesis
GO:0061418	0.00511409	6.65930831	0.7181566	4	22	regulation of transcription from RNA polymerase II promoter in response to hypoxia
GO:0050804	0.00514245	2.8028028	3.85193087	10	118	regulation of synaptic transmission
GO:0050769	0.0052729	2.98934399	3.2643482	9	100	positive regulation of neurogenesis
GO:0001508	0.00545677	2.77677907	3.88457435	10	119	regulation of action potential
GO:0043269	0.00549479	2.39402213	5.81053979	13	178	regulation of ion transport
GO:0001837	0.00551933	3.57479735	2.15446981	7	66	epithelial to mesenchymal transition
GO:0048167	0.00551933	3.57479735	2.15446981	7	66	regulation of synaptic plasticity
GO:0007015	0.00552988	2.49426894	5.15767015	12	158	actin filament organization
GO:0030336	0.00554937	3.21897092	2.709409	8	83	negative regulation of cell migration
GO:0034762	0.00554937	3.21897092	2.709409	8	83	regulation of transmembrane transport
GO:0031333	0.00602882	4.01022222	1.66481758	6	51	negative regulation of protein complex assembly

GO:0043266	0.0060342	6.3081213	0.75080009	4	23	regulation of potassium ion transport
GO:0043009	0.00610326	1.81826977	14.6242799	25	448	chordate embryonic development
GO:0051051	0.0063091	2.35047944	5.90847024	13	181	negative regulation of transport
GO:0001890	0.00639228	2.89297228	3.36227864	9	103	placenta development
GO:0048747	0.00640295	3.13466399	2.77469597	8	85	muscle fiber development
GO:0051253	0.0064117	1.66003937	21.8384894	34	669	negative regulation of RNA metabolic process
GO:0010717	0.00663479	4.69061462	1.20780883	5	37	regulation of epithelial to mesenchymal transition
GO:0061097	0.00663479	4.69061462	1.20780883	5	37	regulation of protein tyrosine kinase activity
GO:0007160	0.00680337	2.86220096	3.39492213	9	104	cell-matrix adhesion
GO:0009792	0.0068131	1.80040338	14.7548539	25	452	embryo development ending in birth or egg hatching
GO:0003206	0.00686537	3.09413182	2.80733945	8	86	cardiac chamber morphogenesis
GO:0019228	0.00686537	3.09413182	2.80733945	8	86	regulation of action potential in neuron
GO:2000146	0.00686537	3.09413182	2.80733945	8	86	negative regulation of cell motility
GO:0048754	0.00686713	2.67729012	4.01514828	10	123	branching morphogenesis of an epithelial tube
GO:0060428	0.00705815	5.99205298	0.78344357	4	24	lung epithelium development
GO:0090130	0.00705815	5.99205298	0.78344357	4	24	tissue migration
GO:2001257	0.00705815	5.99205298	0.78344357	4	24	regulation of cation channel activity
GO:0051050	0.00708283	1.96878285	10.2500533	19	314	positive regulation of transport
GO:0051130	0.00715643	1.89731408	11.7516535	21	360	positive regulation of cellular component organization
GO:0010464	0.00744056	4.54797141	1.24045231	5	38	regulation of mesenchymal cell proliferation
GO:0050731	0.00760905	3.34633965	2.28504374	7	70	positive regulation of peptidyl-tyrosine phosphorylation
GO:0000122	0.00767706	1.82799026	13.3511841	23	409	negative regulation of transcription from RNA polymerase II promoter
GO:0007620	0.00772299	8.96831683	0.42436527	3	13	copulation
GO:0060487	0.00772299	8.96831683	0.42436527	3	13	lung epithelial cell differentiation
GO:0060841	0.00772299	8.96831683	0.42436527	3	13	venous blood vessel development
GO:0070528	0.00772299	8.96831683	0.42436527	3	13	protein kinase C signaling cascade

GO:0009890	0.00794206	1.59104075	25.4619159	38	780	negative regulation of biosynthetic process
GO:0030010	0.00797112	3.75833333	1.76274803	6	54	establishment of cell polarity
GO:0001655	0.00803573	2.13267174	7.47535737	15	229	urogenital system development
GO:0007600	0.00809867	2.07377843	8.19351397	16	251	sensory perception
GO:0051963	0.00819058	5.70608641	0.81608705	4	25	regulation of synapse assembly
GO:0060674	0.00819058	5.70608641	0.81608705	4	25	placenta blood vessel development
GO:0045934	0.00825236	1.60633094	23.8623853	36	731	negative regulation of nucleobase-containing compound metabolic process
GO:0010629	0.00830547	1.61621966	23.0462983	35	706	negative regulation of gene expression
GO:0002576	0.0083121	4.41371898	1.2730958	5	39	platelet degranulation
GO:0060688	0.0083121	4.41371898	1.2730958	5	39	regulation of morphogenesis of a branching structure
GO:0051146	0.00849822	2.34707044	5.45146149	12	167	striated muscle cell differentiation
GO:0001934	0.00882758	1.80357514	13.5144015	23	414	positive regulation of protein phosphorylation
GO:0032409	0.00884976	3.242655	2.3503307	7	72	regulation of transporter activity
GO:0052548	0.00905812	2.43080539	4.83123533	11	148	regulation of endopeptidase activity
GO:0010562	0.00912468	1.75295341	15.1139322	25	463	positive regulation of phosphorus metabolic process
GO:0045937	0.00912468	1.75295341	15.1139322	25	463	positive regulation of phosphate metabolic process
GO:0001947	0.00925192	4.28713811	1.30573928	5	40	heart looping
GO:0007405	0.00925192	4.28713811	1.30573928	5	40	neuroblast proliferation
GO:0034333	0.00925192	4.28713811	1.30573928	5	40	adherens junction assembly
GO:0007268	0.00936332	1.74875769	15.1465756	25	464	synaptic transmission
GO:0003197	0.00943587	5.4461168	0.84873053	4	26	endocardial cushion development
GO:0007618	0.00943587	5.4461168	0.84873053	4	26	mating
GO:0018108	0.00949422	2.54059732	4.21100917	10	129	peptidyl-tyrosine phosphorylation
GO:0051091	0.00949422	2.54059732	4.21100917	10	129	positive regulation of sequence-specific DNA binding transcription factor activity
GO:0030111	0.00950495	2.41292066	4.86387881	11	149	regulation of Wnt receptor signaling pathway
GO:0051271	0.00956599	2.90612113	2.97055686	8	91	negative regulation of cellular component movement
GO:0021533	0.00959474	8.15211521	0.45700875	3	14	cell differentiation in hindbrain

GO:0045746	0.00959474	8.15211521	0.45700875	3	14	negative regulation of Notch signaling pathway
GO:0051150	0.00959474	8.15211521	0.45700875	3	14	regulation of smooth muscle cell differentiation
GO:0051491	0.00959474	8.15211521	0.45700875	3	14	positive regulation of filopodium assembly
GO:0060479	0.00959474	8.15211521	0.45700875	3	14	lung cell differentiation
GO:0051172	0.00972006	1.5865339	24.1235332	36	739	negative regulation of nitrogen compound metabolic process
GO:0060070	0.0099686	2.39529326	4.8965223	11	150	canonical Wnt receptor signaling pathway
GO:0018212	0.00999797	2.51914414	4.24365266	10	130	peptidyl-tyrosine modification
