# Supplementary material for: Integrative Analysis of DNA Methylation and Gene Expression Data Identifies EPAS1 as a Key Regulator of COPD
Source: PLoS Genet. 2015 Jan 8;11(1):e1004898. doi: 10.1371/journal.pgen.1004898 (PMC4287352; doi:10.1371/journal.pgen.1004898)
Supplement: S7 Table — GO enrichment analysis of the 318 genes that were hypermethylated and upregulated in COPD. (PDF) [file pgen.1004898.s016.pdf]

STable 7. GO enrichment analysis of the 318 genes that were hypermethylated and upregulated in COPD

GOBPID	Pvalue	OddsRatio	ExpCount	Count	Size	Term
GO:0006613	2.27E-13	11.01675297	2.459462343	20	87	cotranslational protein targeting to membrane
GO:0006614	1.98E-12	10.42337095	2.431192661	19	86	SRP-dependent cotranslational protein targeting to membrane
GO:0045047	3.07E-12	10.11900554	2.487732025	19	88	protein targeting to ER
GO:0072599	3.80E-12	9.973344948	2.516001707	19	89	establishment of protein localization to endoplasmic reticulum
GO:0006612	2.17E-11	7.676026619	3.448901216	21	122	protein targeting to membrane
GO:0070972	2.31E-11	8.828342081	2.770428846	19	98	protein localization to endoplasmic reticulum
GO:0006414	1.21E-09	8.54334987	2.374653296	16	84	translational elongation
GO:0006413	5.34E-09	6.499539023	3.36409217	18	119	translational initiation
GO:0006415	1.23E-08	8.555608076	2.063686793	14	73	translational termination
GO:0000184	4.30E-08	6.946923077	2.629080435	15	93	nuclear-transcribed mRNA catabolic process, nonsense-mediated decay
GO:0072594	2.04E-07	4.708755047	4.692767229	19	166	establishment of protein localization to organelle
GO:0006412	2.38E-07	3.310634813	10.03573715	29	355	translation
GO:0006402	7.11E-07	4.771817954	4.127373587	17	146	mRNA catabolic process
GO:0043624	1.06E-06	5.246213592	3.335822488	15	118	cellular protein complex disassembly
GO:0000956	1.32E-06	4.813386881	3.844676766	16	136	nuclear-transcribed mRNA catabolic process
GO:0043241	1.63E-06	5.047850467	3.448901216	15	122	protein complex disassembly
GO:0019080	1.80E-06	5.000555556	3.477170898	15	123	viral genome expression
GO:0019083	1.80E-06	5.000555556	3.477170898	15	123	viral transcription
GO:0032984	2.15E-06	4.618281124	3.986025176	16	141	macromolecular complex disassembly
GO:0006401	3.95E-06	4.150419573	4.664497546	17	165	RNA catabolic process
GO:0022411	9.00E-06	3.549262426	6.021442287	19	213	cellular component disassembly
GO:0019058	1.42E-05	3.917493102	4.607958182	16	163	viral infectious cycle
GO:0006605	2.79E-05	2.736714072	10.14881587	25	359	protein targeting
GO:0016482	0.000109477	2.257121097	15.15254961	31	536	cytoplasmic transport

GO:0070167	0.000154079	7.034108527	1.187326648	7	42	regulation of biomineral tissue development
GO:0051924	0.000273677	4.74451959	2.14849584	9	76	regulation of calcium ion transport
GO:0033365	0.000601131	2.283341658	10.94036697	23	387	protein localization to organelle
GO:0030500	0.00071806	6.371358371	1.102517602	6	39	regulation of bone mineralization
GO:0070838	0.000765851	3.225655767	4.070834222	12	144	divalent metal ion transport
GO:0072511	0.000814388	3.201046093	4.099103904	12	145	divalent inorganic cation transport
GO:0006812	0.000824123	2.225954401	11.19479411	23	396	cation transport
GO:0070169	0.000842396	7.943181818	0.763281417	5	27	positive regulation of biomineral tissue development
GO:0050853	0.001110983	10.72325376	0.480584596	4	17	B cell receptor signaling pathway
GO:0071702	0.001206757	1.644823086	37.65521656	56	1332	organic substance transport
GO:0030001	0.001807182	2.260108581	9.046298272	19	320	metal ion transport
GO:0071695	0.001872645	6.468660969	0.904629827	5	32	anatomical structure maturation
GO:0006816	0.001912828	3.038601132	3.929485812	11	139	calcium ion transport
GO:0044703	0.002021778	1.97301431	14.19138041	26	502	multi-organism reproductive process
GO:0006886	0.002048192	1.945068466	14.95466183	27	529	intracellular protein transport
GO:0031214	0.002091977	4.30871753	1.809259654	7	64	biomineral tissue development
GO:0001657	0.002144506	3.800609948	2.318113932	8	82	ureteric bud development
GO:0016071	0.00215821	1.990434143	13.51290804	25	478	mRNA metabolic process
GO:0010959	0.00249456	3.121985077	3.477170898	10	123	regulation of metal ion transport
GO:0006811	0.002654561	1.861739692	16.76392148	29	593	ion transport
GO:0043277	0.003114433	13.02624046	0.310966503	3	11	apoptotic cell clearance
GO:0030282	0.003299232	4.564210173	1.470023469	6	52	bone mineralization
GO:0030198	0.003528687	2.962596803	3.646788991	10	129	extracellular matrix organization
GO:0042596	0.003617617	7.332123412	0.650202688	4	23	fear response
GO:0043062	0.003730063	2.937581699	3.675058673	10	130	extracellular structure organization
GO:0002548	0.004066455	11.57760814	0.339236185	3	12	monocyte chemotaxis
GO:0022415	0.004360413	1.993433926	11.25133348	21	398	viral reproductive process
GO:0051704	0.004621895	1.669460206	23.85961169	37	844	multi-organism process

GO:0030278	0.004639535	3.044057993	3.194474077	9	113	regulation of ossification
GO:0006364	0.004774212	3.661575842	2.091956475	7	74	rRNA processing
GO:0046907	0.004866248	1.642568824	25.5840623	39	905	intracellular transport
GO:0030199	0.004944254	6.632366357	0.706742052	4	25	collagen fibril organization
GO:0009057	0.005118834	1.750936651	18.34702368	30	649	macromolecule catabolic process
GO:0002063	0.005176911	10.41870229	0.367505867	3	13	chondrocyte development
GO:0045684	0.005176911	10.41870229	0.367505867	3	13	positive regulation of epidermis development
GO:0044265	0.005375787	1.819106849	15.26562833	26	540	cellular macromolecule catabolic process
GO:0033555	0.005669488	4.846688034	1.159056966	5	41	multicellular organismal response to stress
GO:0071300	0.005669488	4.846688034	1.159056966	5	41	cellular response to retinoic acid
GO:0030501	0.005715495	6.330198537	0.735011735	4	26	positive regulation of bone mineralization
GO:0048610	0.005776492	1.939700089	11.5340303	21	408	cellular process involved in reproduction
GO:0071822	0.005898779	1.687810491	20.94783444	33	741	protein complex subunit organization
GO:0030217	0.00605524	3.15481135	2.742159164	8	97	T cell differentiation
GO:0051953	0.006452537	9.470506593	0.395775549	3	14	negative regulation of amine transport
GO:0006446	0.006957312	4.590587045	1.21559633	5	43	regulation of translational initiation
GO:0016072	0.007307748	3.358394393	2.261574568	7	80	rRNA metabolic process
GO:0034754	0.007672259	4.472386588	1.243866012	5	44	cellular hormone metabolic process
GO:0010466	0.007884193	3.745035852	1.75272029	6	62	negative regulation of peptidase activity
GO:0010951	0.007884193	3.745035852	1.75272029	6	62	negative regulation of endopeptidase activity
GO:0019748	0.00789911	8.680343511	0.424045231	3	15	secondary metabolic process
GO:0048534	0.007994187	1.944432763	10.37497333	19	367	hematopoietic or lymphoid organ development
GO:0001558	0.008954185	2.326456153	5.484318327	12	194	regulation of cell growth
GO:0030097	0.00901141	1.95713702	9.753040324	18	345	hemopoiesis
GO:0015031	0.009436495	1.585018249	24.96212929	37	883	protein transport
GO:0022613	0.009557908	2.406905659	4.862385321	11	172	ribonucleoprotein complex biogenesis
GO:0048608	0.009673559	2.300653033	5.540857691	12	196	reproductive structure development
GO:0061458	0.009673559	2.300653033	5.540857691	12	196	reproductive system development
