# Supplementary material for: Integrative Analysis of DNA Methylation and Gene Expression Data Identifies EPAS1 as a Key Regulator of COPD
Source: PLoS Genet. 2015 Jan 8;11(1):e1004898. doi: 10.1371/journal.pgen.1004898 (PMC4287352; doi:10.1371/journal.pgen.1004898)
Supplement: S8 Table — 67 key regulators in CTRL lung tissues that regulated a large number of downstream genes. (PDF) [file pgen.1004898.s017.pdf]

STable 8. 67 key regulators in CTRL lung tissues that regulated a large number of downstream genes.

cis Gene	rho (cis methyl-mRNA correlation)	p-value (cis methyl-mRNA correlation)	# of down-stream genes
WDR90	-0.54	2.60E-05	318
SATB2	0.52	9.20E-05	228
COL12A1	-0.54	3.90E-05	206
FOXK2	0.62	8.00E-07	206
MAP4K4	-0.46	0.0005	201
TTC16	-0.48	0.0004	188
HEATR2	-0.55	2.20E-05	179
NHLRC4	-0.5	0.0001	177
OSTalpha	-0.55	2.30E-05	175
SCRN2	-0.39	0.0045	168
FAM83H	0.47	0.0005	162
RARG	-0.57	9.30E-06	162
HTRA2	-0.43	0.0013	158
EPAS1	-0.48	0.0003	157
WWP1	-0.36	0.0085	156
ERGIC3	-0.51	9.80E-05	150
TMED3	-0.51	0.0001	145
SEMA4F	-0.49	0.0002	151
FBXW7	0.47	0.0004	139
SETD1B	-0.45	0.0008	134
ARFGEF1	0.42	0.0018	133
ARRDC1	-0.5	0.0001	132
SPA17	-0.47	0.0005	130
PLXNB2	0.46	0.0005	127
GAK	-0.39	0.0048	124
INPPL1	-0.46	0.0006	122
EML3	-0.5	0.0002	121
PRKAA2	-0.36	0.008	119
DDX31	-0.32	0.0019	117
PSENEN	0.47	0.0004	113
NHLRC3	0.48	0.0003	111
NAT14	-0.55	2.00E-05	110
GIGYF1	-0.43	0.0015	109
ATG16L2	0.48	0.0003	104
CPNE8	0.47	0.0003	104
SP3	0.48	0.0003	103
ATN1	-0.46	0.0006	98
PRKD3	0.42	0.0016	98
BAG6	-0.39	0.0042	97

VPS52	-0.47	0.0005	97
ELL3	-0.5	0.0001	95
GNB2	-0.43	0.0014	91
LIG4	0.44	0.0012	91
NFYC	-0.49	0.0002	90
HOOK2	-0.5	0.0001	89
KLHL22	-0.45	0.0007	89
RNF31	-0.56	1.40E-05	89
ZEB2	-0.5	0.0002	89
OSGIN2	0.48	0.0003	87
TBCD	-0.48	0.0003	86
ZBTB9	-0.47	0.0005	86
ZYX	-0.4	0.0032	86
FOXJ1	-0.5	0.0002	85
PPP2R5D	-0.38	0.0053	85
PCGF2	-0.4	0.0033	84
EN2	-0.4	0.0031	83
MORN1	-0.5	0.0002	83
RAB5B	-0.46	0.0006	83
REPIN1	-0.46	0.0006	82
C13orf15	0.52	7.70E-05	80
SCAF8	0.5	0.0002	80
ARHGEF10	0.44	0.001	79
EFCAB1	-0.47	0.0004	79
MOBKL1A	0.43	0.0013	79
PITPNM1	-0.4	0.003	79
YOD1	0.45	0.0007	79
LRP1	-0.35	0.0098	78
