# Supplementary material for: Integrative Analysis of DNA Methylation and Gene Expression Data Identifies EPAS1 as a Key Regulator of COPD
Source: PLoS Genet. 2015 Jan 8;11(1):e1004898. doi: 10.1371/journal.pgen.1004898 (PMC4287352; doi:10.1371/journal.pgen.1004898)
Supplement: S9 Table — 126 key regulators in COPD lung tissues that regulated a large number of downstream genes. (PDF) [file pgen.1004898.s018.pdf]

**STable 9. 126 key regulators in COPD lung tissues that regulated a large number of downstream genes.**

| *cis* Gene | *rho (cis methyl-mRNA correlation)* | p-value (cis methyl-mRNA correlation) | # of down-stream genes |
|---|---|---|---|
| GAK | -0.47 | 1.10E-06 | 1817 |
| ACSF3 | -0.45 | 2.90E-06 | 1549 |
| CLCN7 | -0.41 | 2.50E-05 | 1506 |
| ALG12 | -0.45 | 3.50E-06 | 1357 |
| ABHD14B | -0.45 | 3.00E-06 | 1300 |
| SCRIB | -0.47 | 1.00E-06 | 1255 |
| SSNA1 | -0.44 | 5.60E-06 | 1224 |
| HIST1H2BG | 0.37 | 0.0002 | 1190 |
| SELO | -0.47 | 6.60E-07 | 1131 |
| ZFYVE26 | -0.47 | 6.70E-07 | 1126 |
| THOP1 | -0.35 | 0.0003 | 1124 |
| C6orf226 | -0.38 | 7.90E-05 | 1080 |
| SRI | 0.34 | 0.0005 | 1005 |
| DAGLB | -0.46 | 1.90E-06 | 1004 |
| MED16 | -0.32 | 0.0013 | 1004 |
| TSGA10 | 0.44 | 5.80E-06 | 1001 |
| C13orf27 | 0.37 | 0.0002 | 986 |
| SEC16A | -0.27 | 0.0061 | 975 |
| PMPCA | -0.38 | 8.90E-05 | 966 |
| DEDD2 | 0.4 | 3.50E-05 | 949 |
| SETBP1 | -0.37 | 0.0002 | 899 |
| DNAJA1 | 0.42 | 1.70E-05 | 884 |
| ADRM1 | -0.45 | 2.50E-06 | 858 |
| MATK | -0.34 | 0.0005 | 840 |
| NUDT16L1 | -0.31 | 0.0016 | 827 |
| PLXNB2 | 0.38 | 9.30E-05 | 826 |
| COASY | -0.43 | 7.60E-06 | 815 |
| JMJD1C | 0.38 | 0.0001 | 811 |
| HINT2 | -0.39 | 6.90E-05 | 804 |
| EPAS1 | -0.53 | 7.30E-09 | 780 |
| NADSYN1 | -0.39 | 7.00E-05 | 765 |
| NCAPD2 | -0.42 | 1.40E-05 | 753 |
| MOBKL2A | -0.41 | 1.80E-05 | 751 |
| PAX9 | -0.46 | 1.40E-06 | 751 |
| C3orf26 | 0.51 | 8.00E-08 | 750 |
| ATAD1 | 0.3 | 0.0024 | 745 |
| RPS6 | 0.37 | 0.0001 | 742 |
| TMEM102 | -0.39 | 5.50E-05 | 741 |
| FOXK2 | -0.44 | 4.40E-06 | 739 |
| C19orf29 | -0.43 | 7.50E-06 | 713 |

| | | | |
|---|---|---|---|
| MAP3K8 | 0.39 | 5.70E-05 | 710 |
| NCLN | 0.36 | 0.0002 | 697 |
| B4GALT7 | -0.38 | 7.70E-05 | 695 |
| YOD1 | 0.46 | 1.50E-06 | 688 |
| FKBP7 | 0.52 | 2.10E-08 | 686 |
| FIP1L1 | 0.42 | 1.40E-05 | 684 |
| BRD9 | -0.43 | 8.70E-06 | 681 |
| ERGIC2 | 0.41 | 2.10E-05 | 679 |
| TMEM42 | -0.45 | 2.10E-06 | 649 |
| FLI1 | 0.48 | 4.70E-07 | 639 |
| MVD | -0.35 | 0.0004 | 638 |
| NEK8 | 0.36 | 0.0002 | 635 |
| DDX59 | 0.38 | 8.80E-05 | 628 |
| AXIN1 | 0.35 | 0.0003 | 626 |
| EFNA3 | -0.42 | 1.30E-05 | 626 |
| REV3L | -0.51 | 6.30E-08 | 606 |
| MUTED | 0.47 | 1.10E-06 | 600 |
| RBM8A | 0.33 | 0.0008 | 600 |
| ZC3H18 | -0.45 | 3.30E-06 | 600 |
| PCYT2 | -0.46 | 1.30E-06 | 595 |
| EXOC8 | -0.37 | 0.0002 | 580 |
| MAP3K7 | 0.42 | 1.20E-05 | 579 |
| AASDHPPT | 0.45 | 3.00E-06 | 576 |
| USP46 | 0.39 | 6.60E-05 | 574 |
| UCKL1 | 0.41 | 2.30E-05 | 569 |
| TMC6 | 0.37 | 0.0001 | 557 |
| CCNA1 | -0.45 | 2.30E-06 | 556 |
| CSDE1 | 0.43 | 7.40E-06 | 543 |
| AASS | 0.35 | 0.0004 | 532 |
| CGGBP1 | 0.36 | 0.0002 | 532 |
| DNAH3 | -0.44 | 5.30E-06 | 531 |
| MRPL12 | -0.35 | 0.0004 | 531 |
| PPP1CA | -0.4 | 3.90E-05 | 531 |
| SPHK2 | 0.44 | 3.80E-06 | 530 |
| IFIH1 | 0.27 | 0.0072 | 521 |
| WT1-AS | 0.46 | 1.60E-06 | 519 |
| RUNX1T1 | 0.37 | 0.0001 | 516 |
| HAS2 | 0.32 | 0.0014 | 515 |
| SLC4A2 | 0.47 | 7.00E-07 | 514 |
| MEN1 | -0.4 | 4.50E-05 | 510 |
| C16orf70 | -0.43 | 8.80E-06 | 508 |
| EZR | 0.49 | 4.90E-07 | 506 |
| COQ5 | -0.36 | 0.0003 | 505 |
| WDR83 | -0.35 | 0.0004 | 504 |
| C9orf142 | -0.39 | 6.90E-05 | 503 |

| | | | |
|---|---|---|---|
| TUBGCP2 | 0.37 | 0.0002 | 503 |
| C2orf3 | 0.29 | 0.004 | 496 |
| DOT1L | -0.37 | 0.0001 | 496 |
| HENMT1 | -0.41 | 2.70E-05 | 494 |
| MATR3 | 0.48 | 3.70E-07 | 491 |
| RPH3AL | -0.35 | 0.0004 | 491 |
| TMCO6 | -0.36 | 0.0003 | 491 |
| EXOSC8 | 0.43 | 7.00E-06 | 487 |
| ASCL2 | -0.48 | 5.00E-07 | 486 |
| C13orf23 | 0.37 | 0.0001 | 485 |
| TRAP1 | -0.31 | 0.0016 | 483 |
| HNRNPF | 0.41 | 1.80E-05 | 479 |
| IFT140 | -0.34 | 0.0005 | 478 |
| EOMES | 0.36 | 0.0002 | 477 |
| GOLGA5 | 0.44 | 5.30E-06 | 474 |
| MYCBP2 | -0.51 | 6.10E-08 | 473 |
| BRAT1 | -0.43 | 6.30E-06 | 472 |
| BCL3 | -0.37 | 0.0002 | 468 |
| HOXA7 | -0.45 | 3.30E-06 | 462 |
| DLEU1 | 0.41 | 1.90E-05 | 461 |
| RG9MTD1 | 0.38 | 8.70E-05 | 461 |
| GALNS | -0.35 | 0.0004 | 458 |
| UAP1L1 | -0.29 | 0.0039 | 458 |
| HMGN4 | 0.38 | 0.0001 | 454 |
| RNH1 | -0.37 | 0.0002 | 453 |
| FAT4 | 0.37 | 0.0002 | 447 |
| EXOC5 | 0.41 | 2.60E-05 | 444 |
| KEAP1 | 0.35 | 0.0004 | 444 |
| EIF2AK1 | -0.4 | 4.50E-05 | 442 |
| GPR108 | 0.34 | 0.0005 | 439 |
| HEATR2 | -0.34 | 0.0006 | 437 |
| SLC7A7 | -0.35 | 0.0004 | 437 |
| MIPOL1 | 0.26 | 0.0083 | 435 |
| MRPL38 | -0.34 | 0.0005 | 434 |
| NDUFS7 | 0.31 | 0.0017 | 432 |
| JMJD8 | -0.31 | 0.0015 | 430 |
| DNAJC2 | 0.33 | 0.0007 | 429 |
| ETF1 | 0.31 | 0.0015 | 429 |
| NCSTN | 0.42 | 1.50E-05 | 428 |
| PPIL6 | -0.48 | 5.30E-07 | 428 |
| KHSRP | 0.41 | 1.80E-05 | 427 |
