# Supplementary material for: Integrative Analysis of DNA Methylation and Gene Expression Data Identifies EPAS1 as a Key Regulator of COPD
Source: PLoS Genet. 2015 Jan 8;11(1):e1004898. doi: 10.1371/journal.pgen.1004898 (PMC4287352; doi:10.1371/journal.pgen.1004898)
Supplement: S10 Table — Correlation between key regulators' methylation levels in promoter regions in COPD lung tissues and COPD severity Traits. (PDF) [file pgen.1004898.s019.pdf]

STable 10. Correlation between key regulators' methylation levels in promoter regions in COPD lung tissues and COPD severity traits

Regulator	DLCO_rho	DLCO_pvalue	BODE_rho	BODE_pvalue	FEV1_rho	FEV1_pvalue	Ratio_rho	Ratio_pvalue	Emphysema_rho	Emphysema_pvalue
GAK	0.2241	0.039211198	-0.335	0.000758834	-0.233	0.036337245	-0.184	0.099344969	-0.26448591	0.03777094
ACSF3	-0.31	0.003913845	-0.363	0.000236026	-0.254	0.022074892	-0.326	0.00297938	-0.37437989	0.00272008
CLCN7	0.2356	0.029924631	-0.255	0.011362673	-0.133	0.237905121	0.2566	0.020750151	-0.36425676	0.00360851
ALG12	0.2997	0.005327641	-0.333	0.000793572	0.166	0.137488414	0.1797	0.10845515	-0.44645061	0.00027567
ABHD14B	0.1362	0.214001273	0.1258	0.217269319	-0.107	0.339765427	0.1294	0.249408864	0.33565007	0.00765367
SCRIB	-0.238	0.0285243	-0.304	0.002311767	-0.173	0.123302682	-0.118	0.295208178	-0.27586815	0.0299851
SSNA1	0.1594	0.145136503	0.1932	0.056657131	-0.196	0.078942297	0.1111	0.323643026	-0.21170457	0.09857113
HIST1H2BG	0.1956	0.072802502	-0.171	0.092913316	0.119	0.290933581	0.2	0.073474546	-0.25819043	0.04275304
SELO	0.2577	0.017284891	-0.338	0.000674921	0.293	0.008040592	0.2575	0.020277642	-0.33539825	0.00770221
ZFYVE26	-0.185	0.0896718	0.3198	0.001325651	-0.271	0.014222025	-0.267	0.015929095	0.36737932	0.00331038
THOP1	0.172	0.115580332	-0.341	0.000590648	0.147	0.189941104	0.1449	0.196909028	-0.39497872	0.00148793
C6orf226	-0.105	0.340556731	0.2161	0.032593954	-0.164	0.142425809	-0.11	0.330327855	0.2653421	0.03713216
SRI	0.2861	0.007938028	-0.217	0.031543946	0.149	0.184194888	0.1555	0.165688698	-0.1412455	0.27350116
DAGLB	0.2251	0.038314839	-0.216	0.033014633	-0.092	0.41432996	0.1296	0.248943572	-0.24605273	0.05388707
MED16	0.25	0.021000597	-0.236	0.019207324	-0.157	0.160760373	0.232	0.037135736	-0.34980232	0.00532104
TSGA10	0.2601	0.016205475	-0.089	0.38487419	0.083	0.460274869	0.2187	0.0498207	-0.21734532	0.0897153
C13orf27	0.2144	0.048814884	0.1942	0.055303306	-0.157	0.161827978	-0.198	0.077083916	0.32129637	0.01088689
SEC16A	0.2265	0.037090399	-0.369	0.000187762	0.206	0.064945631	0.1636	0.144431938	-0.35901891	0.00416229
PMPCA	0.243	0.025046231	-0.285	0.004506989	0.187	0.094738555	0.1417	0.206871898	-0.38888469	0.00178583
DEDD2	-0.283	0.008629152	0.1354	0.183754082	0.162	0.149359168	-0.209	0.06087576	0.29606406	0.01946969
SETBP1	0.1553	0.155918822	-0.233	0.021105949	0.202	0.070346031	0.1842	0.099709517	-0.34385938	0.00621088
DNAJA1	0.1476	0.17776916	0.0606	0.553191682	-0.097	0.39011103	0.0548	0.626763387	-0.16824054	0.19117632
ADRM1	0.1612	0.140495922	-0.276	0.005940566	0.166	0.13792198	-0.147	0.18979729	-0.3230591	0.01043485
MATK	0.2086	0.055436098	-0.299	0.002748998	-0.232	0.03676777	0.1505	0.179866478	-0.36345093	0.00368921
NUDT16L1	0.1985	0.068613583	0.2836	0.004661602	-0.237	0.033387967	0.1443	0.198599224	-0.27863816	0.0283088

PLXNB2	0.2026	0.062956029	-0.181	0.075172532	0.214	0.054640282	0.2324	0.036847583	-0.1688449	0.1895636
COASY	-0.207	0.05693794	0.1802	0.07582905	-0.135	0.22875576	-0.316	0.004004461	0.20782655	0.10504404
JMJD1C	0.0839	0.445113229	0.1043	0.30696175	-0.148	0.188603696	-0.056	0.617832323	0.14084259	0.27488582
HINT2	0.0982	0.371074043	-0.099	0.334062029	-0.077	0.493852041	0.123	0.27397716	0.09964493	0.44096887
EPAS1	-0.285	0.008127095	0.2944	0.003253549	-0.311	0.004713931	-0.223	0.044996958	0.26060789	0.04077941
NADSYN1	0.1967	0.071254796	-0.199	0.049848034	-0.168	0.134695324	0.1357	0.22710834	-0.33439097	0.00789907
NCAPD2	0.2074	0.056806558	-0.095	0.353406184	-0.109	0.333722668	0.1297	0.248605551	0.1027675	0.42671131
MOBKL2A	0.2373	0.028760538	-0.264	0.008603866	-0.166	0.138383776	0.1225	0.276057503	-0.33590189	0.0076054
PAX9	-0.192	0.078689173	-0.156	0.1255235	-0.188	0.09362699	-0.295	0.007532742	0.26131299	0.04021815
C3orf26	0.1733	0.112750744	0.0607	0.55293874	0.056	0.620790987	0.1288	0.251829473	0.18108333	0.15898047
ATAD1	0.0822	0.454262786	-0.088	0.388583765	-0.046	0.68092044	0.0338	0.764536807	0.05436781	0.67471114
RPS6	0.2868	0.007783438	-0.158	0.121022875	-0.16	0.153933127	0.0992	0.378327715	-0.24841983	0.05154793
TMEM102	0.2197	0.043382629	-0.25	0.013090166	-0.11	0.326950313	0.1165	0.300356684	-0.33136914	0.00851622
FOXK2	0.2236	0.039652586	0.2697	0.00724604	0.175	0.118203738	0.2267	0.041869403	-0.31444688	0.01280818
C19orf29	0.1937	0.075632285	-0.29	0.003785041	0.129	0.249923506	0.1527	0.173405301	-0.33308151	0.00816154
MAP3K8	0.1876	0.085595622	0.1142	0.263035874	-0.143	0.202143269	0.1725	0.123484303	0.18838609	0.14255824
NCLN	0.2001	0.06634923	-0.238	0.018265881	-0.177	0.114699117	-0.225	0.043043548	-0.29007076	0.02219882
B4GALT7	0.1281	0.242818163	-0.122	0.230408003	-0.169	0.131055792	0.1226	0.275604359	-0.25310367	0.04716346
YOD1	0.0834	0.448151561	0.1942	0.055377358	-0.159	0.156482464	-0.155	0.1662585	0.21739568	0.08963914
FKBP7	0.217	0.046077726	-0.161	0.112366534	-0.189	0.091869858	-0.193	0.084086914	-0.21387021	0.09509395
FIP1L1	0.1739	0.111499137	-0.087	0.3924196	0.142	0.207075336	0.1166	0.299925413	-0.23064138	0.07130749
BRD9	0.2149	0.048279952	-0.3	0.002675766	-0.173	0.121963009	-0.153	0.171516752	-0.27838634	0.02845786
ERGIC2	0.0335	0.760745668	0.1045	0.306032931	-0.113	0.316102904	0.0679	0.546820653	0.09385309	0.46810587
TMEM42	-0.155	0.155550057	0.2259	0.025342542	-0.174	0.119438688	0.0491	0.663607928	0.23069175	0.071244
FLI1	0.194	0.075226996	-0.234	0.020359932	0.115	0.305919568	0.1161	0.302085819	-0.18395407	0.15236595
MVD	0.1553	0.155839747	-0.211	0.036629309	0.073	0.51535088	0.1	0.374568838	-0.26252172	0.03927086
NEK8	0.2425	0.025367239	0.1391	0.171801338	-0.106	0.347635109	-0.206	0.064630616	0.17786004	0.16665703
DDX59	0.0958	0.38319664	0.0431	0.673619971	-0.07	0.534801794	0.1002	0.373357596	0.11299136	0.38190397
AXIN1	0.1929	0.076950129	-0.277	0.005717036	0.101	0.371703289	-0.091	0.41744991	-0.35549344	0.00457612

EFNA3	0.2472	0.022578209	-0.168	0.097757373	0.184	0.099331	0.2471	0.026166235	-0.21714386	0.09002044
REV3L	0.2218	0.041340239	0.0491	0.63134727	-0.101	0.371217607	0.1689	0.131719541	-0.24157035	0.05855287
MUTED	0.2203	0.042755641	-0.181	0.07521475	0.177	0.114534253	0.0806	0.474579396	-0.19583994	0.12714018
RBM8A	0.1124	0.305663307	0.1748	0.085115643	-0.146	0.192185323	0.1578	0.159546445	0.17604694	0.17109268
ZC3H18	0.1916	0.078953495	-0.26	0.009656237	0.111	0.325755571	0.1254	0.264613647	-0.38515776	0.00199334
PCYT2	0.1854	0.089327795	-0.166	0.102541115	0.103	0.358514511	0.1379	0.219426285	-0.25652842	0.04415506
EXOC8	-0.199	0.068280399	0.1167	0.252652412	-0.067	0.553936568	-0.164	0.144061255	0.11017099	0.39397433
MAP3K7	-0.054	0.623657644	-0.122	0.232936783	-0.149	0.184026826	-0.136	0.225841446	0.08745688	0.49909234
AASDHPPT	0.1189	0.278563221	-0.131	0.200253318	-0.193	0.084876195	-0.089	0.430374247	0.20359598	0.11247532
USP46	0.2123	0.051080199	-0.187	0.065805087	-0.115	0.307402175	0.1588	0.156857946	-0.13520183	0.29476067
UCKL1	-0.212	0.050916354	-0.229	0.023096	0.224	0.044135559	0.2526	0.022920822	-0.29042331	0.02202976
TMC6	0.2088	0.055167608	0.1244	0.222310305	0.152	0.17482463	0.226	0.042490823	0.1586714	0.21803322
CCNA1	0.2117	0.051828407	0.1284	0.207821364	0.109	0.33422783	-0.158	0.159454232	-0.18163733	0.15768776
CSDE1	-0.11	0.314504985	0.1369	0.178906363	-0.198	0.076546808	-0.214	0.055557475	0.19019919	0.13868509
AASS	0.0793	0.470782709	0.1471	0.148264144	-0.147	0.18904874	-0.118	0.295492676	0.20420035	0.11138965
CGGBP1	0.1342	0.220882498	-0.23	0.022789396	-0.167	0.136947907	-0.112	0.320083417	0.10055148	0.43680241
DNAH3	0.0777	0.479661843	-0.107	0.293658948	0.095	0.400248384	-0.135	0.22913771	0.10191131	0.43059434
MRPL12	0.194	0.075167102	-0.17	0.093703028	-0.077	0.495312871	0.0963	0.39262523	-0.22857646	0.07394983
PPP1CA	0.2012	0.064846937	-0.246	0.014575525	-0.085	0.44964903	0.1412	0.208473282	-0.2334114	0.06788195
SPHK2	-0.161	0.14078874	0.1601	0.115369451	0.118	0.29362164	-0.209	0.060933715	0.15358465	0.23333881
IFIH1	0.1654	0.130396141	-0.173	0.087710485	0.137	0.222076215	0.1242	0.269401791	0.07073607	0.58484156
WT1-AS	-0.253	0.019459904	0.2437	0.015611254	-0.224	0.044409972	-0.183	0.101613344	0.28397673	0.02529958
RUNX1T1	0.238	0.028296385	-0.183	0.07123778	0.181	0.10572313	0.1925	0.0850488	-0.45249427	0.00022211
HAS2	0.0987	0.368687401	0.1224	0.229766495	-0.157	0.161430828	-0.119	0.291902339	0.19745159	0.12397946
SLC4A2	0.2317	0.032846753	-0.346	0.00047481	0.217	0.051431278	0.2248	0.04364629	-0.25632696	0.04432754
MEN1	-0.041	0.710986408	0.1703	0.093539639	-0.131	0.243678775	0.0654	0.561993577	-0.09334945	0.47050744
C16orf70	0.2487	0.021715782	0.1266	0.214016459	-0.146	0.19357585	0.1537	0.170836705	0.15690867	0.22325538
EZR	0.2276	0.036210124	-0.18	0.076511568	0.164	0.143652791	0.2277	0.040930528	-0.22580645	0.0776163
COQ5	0.2126	0.050774705	-0.114	0.263441987	0.11	0.329298093	0.2082	0.062190628	-0.18284606	0.15489429

WDR83	0.172	0.115412382	-0.177	0.080576885	-0.019	0.866479368	0.0316	0.779562795	-0.16476543	0.20064064
C9orf142	0.2611	0.015782981	-0.125	0.221588594	0.148	0.187069014	0.2029	0.069211082	-0.19614213	0.12654294
TUBGCP2	-0.28	0.009326239	0.2174	0.031549098	-0.18	0.107325345	-0.228	0.040794865	-0.28337237	0.0256261
C2orf3	0.1303	0.234612755	0.0684	0.503462383	-0.065	0.562642991	0.109	0.332831495	0.14895117	0.24791299
DOT1L	0.2441	0.024385106	-0.283	0.004771929	-0.18	0.107549359	-0.212	0.057978934	-0.3653144	0.00350497
HENMT1	0.2694	0.012644964	0.1916	0.058777552	0.088	0.436901986	0.2063	0.064661029	0.16295233	0.20570876
MATR3	0.1473	0.178438072	-0.074	0.466601948	-0.105	0.350595789	0.1296	0.248816777	-0.20158143	0.11615303
RPH3AL	-0.167	0.127223292	0.3173	0.001452477	-0.23	0.038611028	-0.128	0.25310991	-0.29833044	0.0185149
TMCO6	0.2269	0.036747817	0.1886	0.062910892	0.186	0.096435614	0.281	0.011042933	-0.2670041	0.03591775
EXOSC8	0.193	0.07675205	-0.092	0.367924864	-0.104	0.356616661	-0.111	0.32278843	0.16108887	0.21101146
ASCL2	0.2562	0.017962717	-0.243	0.01588996	0.174	0.119755059	0.2731	0.01363139	-0.29717207	0.01899779
C13orf23	0.026	0.813012197	0.034	0.739794632	-0.09	0.422262719	-0.057	0.613315796	0.20817909	0.10444234
TRAP1	0.096	0.381929969	-0.044	0.663999995	-0.067	0.549909234	0.0984	0.382332437	0.19458085	0.12965174
HNRNPF	0.2012	0.064860227	-0.1	0.328862521	-0.093	0.408137804	0.2048	0.066647256	-0.18611971	0.1475128
IFT140	0.2203	0.042802875	-0.263	0.008960809	0.183	0.101648722	-0.181	0.105371475	-0.46130795	0.00016091
EOMES	0.1368	0.211851466	0.0801	0.433005579	-0.104	0.357353974	-0.089	0.427866673	-0.13132381	0.30895583
GOLGA5	0.1982	0.069045885	-0.122	0.230704505	0.085	0.450312895	0.1456	0.194588581	-0.14220241	0.27023126
MYCBP2	0.1324	0.227224796	0.1476	0.14688903	-0.177	0.114793407	-0.205	0.066150011	0.22580645	0.0776163
BRAT1	0.0789	0.472815338	-0.103	0.313758191	-0.145	0.197613948	0.1307	0.244866303	0.19770341	0.12349104
BCL3	-0.181	0.097324282	-0.111	0.278149305	0.144	0.20074455	-0.12	0.285967124	0.06892297	0.59452573
HOXA7	0.1434	0.190366879	-0.114	0.265778582	0.103	0.36163832	-0.168	0.134591499	-0.19991942	0.1192559
DLEU1	-0.099	0.36503036	-0.104	0.306242504	0.087	0.438149592	-0.138	0.218768714	-0.19644431	0.12594783
RG9MTD1	0.0853	0.437383664	0.0679	0.506442927	-0.02	0.858611525	-0.049	0.661982037	0.08216867	0.52549056
GALNS	-0.185	0.090884071	-0.322	0.001208963	0.09	0.423311428	-0.077	0.492046938	-0.33962882	0.00692145
UAP1L1	0.1158	0.29120835	0.082	0.421901835	0.157	0.162624447	0.1639	0.143833498	-0.04766941	0.71293299
HMGN4	-0.131	0.230565697	-0.162	0.11002243	0.13	0.247885167	-0.15	0.182024845	-0.16345597	0.20429194
RNH1	-0.169	0.122805974	0.2433	0.015767145	-0.198	0.075727353	0.1951	0.080924655	-0.19629322	0.12624512
FAT4	0.1989	0.067976129	-0.228	0.024197033	0.164	0.143345303	0.1495	0.182737149	-0.30276246	0.01676276
EXOC5	0.1327	0.226187749	-0.086	0.40253348	0.141	0.209868509	0.1291	0.25072335	-0.18108333	0.15898047

KEAP1	0.2254	0.038048433	0.1552	0.127014708	0.15	0.180125915	0.1544	0.168872565	0.18843645	0.14244957
EIF2AK1	0.1491	0.173280511	-0.048	0.638840152	0.05	0.657269098	0.2025	0.069872394	-0.13968422	0.27889264
GPR108	-0.188	0.085264068	0.2027	0.045282284	-0.059	0.599286217	0.0876	0.436981207	0.22117297	0.08407043
HEATR2	0.224	0.039290344	-0.278	0.005497616	-0.206	0.064600868	-0.133	0.234773491	-0.2778827	0.02875797
SLC7A7	-0.207	0.057309533	0.2044	0.043553143	-0.141	0.207734473	0.1539	0.170061935	-0.10911334	0.3985581
MIPOL1	0.0992	0.366405068	0.0099	0.92277314	0.081	0.470913288	0.1496	0.182431628	0.0117096	0.92802644
MRPL38	0.205	0.059854317	-0.265	0.008253023	0.091	0.417157241	0.1531	0.172426571	-0.31207978	0.01353692
NDUFS7	0.127	0.246939263	-0.199	0.049878242	-0.115	0.30677987	0.1325	0.23848797	-0.2254539	0.07809312
JMJD8	0.1598	0.14394086	-0.3	0.002727806	0.115	0.307258493	0.1414	0.207988216	-0.29425096	0.02026341
DNAJC2	0.1924	0.077761918	0.1663	0.101653095	-0.13	0.247387727	-0.104	0.354196002	0.10518496	0.41585549
ETF1	0.1847	0.090605846	-0.019	0.855123675	0.043	0.701686097	0.175	0.118151791	-0.13751857	0.28648707
NCSTN	0.0259	0.814321832	-0.157	0.12179251	0.132	0.238631702	0.0221	0.844404003	-0.18143587	0.15815694
PPIL6	-0.26	0.016309481	0.1216	0.232911898	-0.156	0.164411633	0.1574	0.160439874	-0.19578958	0.12723993
KHSRP	-0.268	0.013190391	0.3138	0.001651304	-0.187	0.093908955	-0.178	0.111936998	0.38913651	0.00177254
