# Supplementary material for: Integrative Analysis of DNA Methylation and Gene Expression Data Identifies EPAS1 as a Key Regulator of COPD
Source: PLoS Genet. 2015 Jan 8;11(1):e1004898. doi: 10.1371/journal.pgen.1004898 (PMC4287352; doi:10.1371/journal.pgen.1004898)
Supplement: S11 Table — Overlap between downstream genes of key regulators in COPD lung tissues and COPD severity signatures in Human. (PDF) [file pgen.1004898.s020.pdf]

STable 11. Overlap between downstream genes of key regulators in COPD lung tissues and COPD severity signatures in human

Regulator	Downstream	DLCO_overlap	DLCO_pvalue	BODE_overlap	BODE_pvalue	FEV1_overlap	FEV1_pvalue	Ratio_overlap	Ratio_pvalue	Emphysema_overlap	Emphysema_pvalue
GAK	1817	32	1	96	0.99969	37	0.99984	26	0.99117	108	1
ACSF3	1549	35	0.99939	106	0.69323	14	1	10	1	105	0.999999513
CLCN7	1506	25	1	78	0.99947	17	1	8	1	100	0.999999758
ALG12	1357	39	0.94612	149	3.63E-08	11	1	6	1	113	0.992089698
ABHD14B	1300	17	1	31	1	9	1	5	1	138	0.288805616
SCRIB	1255	16	1	60	0.99985	8	1	1	1	70	0.999999999
SSNA1	1224	23	0.99991	62	0.99907	7	1	3	1	57	1
HIST1H2BG	1190	17	1	46	1	3	1	0	1	74	0.999999723
SELO	1131	29	0.98222	92	0.09751	8	1	0	1	70	0.999999552
ZFYVE26	1126	34	0.87989	83	0.38927	40	0.37076	19	0.87981	224	1.22E-24
THOP1	1124	31	0.95518	134	1.23E-09	16	0.99999	7	0.99999	109	0.70898873
C6orf226	1080	26	0.99123	49	0.99989	29	0.91413	14	0.9857	172	5.58E-10
SRI	1005	30	0.88027	65	0.81706	3	1	1	1	52	0.999999998
DAGLB	1004	18	0.99982	50	0.9983	3	1	0	1	47	1
MED16	1004	30	0.87905	102	0.00015	6	1	4	1	85	0.971483759
TSGA10	1001	10	1	34	1	5	1	1	1	43	1
C13orf27	986	18	0.99973	36	1	4	1	5	1	145	1.91E-06
SEC16A	974	30	0.83769	111	3.94E-07	7	1	3	1	101	0.418399012
PMPCA	966	25	0.97109	91	0.00348	16	0.99967	6	0.99997	48	0.999999999
DEDD2	949	14	0.99998	21	1	1	1	4	1	61	0.999986414
SETBP1	899	14	0.99995	30	1	6	1	4	1	32	1
DNAJA1	884	8	1	12	1	6	1	0	1	91	0.454045582
ADRM1	858	33	0.37005	130	5.26E-17	19	0.982	12	0.95639	100	0.074348286
MATK	840	10	1	28	1	6	1	2	1	61	0.998730138
NUDT16L1	827	21	0.96745	61	0.40868	18	0.98345	8	0.99706	66	0.98714083

PLXNB2	826	5	1	17	1	6	1	5	0.99992	70	0.956873826
COASY	814	12	0.99994	27	1	3	1	4	0.99998	79	0.680164978
JMJD1C	811	15	0.99902	43	0.98689	5	1	5	0.9999	45	0.999999415
HINT2	804	7	1	26	1	3	1	0	1	86	0.311711167
EPAS1	779	73	3.73E-14	100	4.45E-09	97	2.01E-30	48	2.41E-11	98	0.013729816
NADSYN1	765	12	0.99981	37	0.99681	6	1	3	0.99999	37	0.999999984
NCAPD2	753	11	0.99991	19	1	8	0.99999	3	0.99999	60	0.983829348
MOBKL2A	751	11	0.9999	44	0.93113	3	1	0	1	43	0.999996241
PAX9	751	7	1	18	1	9	0.99996	5	0.9997	30	1
C3orf26	750	9	0.99999	28	0.99998	1	1	1	1	41	0.999999038
ATAD1	745	9	0.99999	21	1	0	1	0	1	51	0.999459463
RPS6	742	28	0.42442	46	0.86133	10	0.99984	0	1	34	0.999999995
TMEM102	741	17	0.98488	47	0.82222	3	1	0	1	27	1
FOXK2	739	26	0.57629	99	5.39E-10	11	0.99954	5	0.99963	90	0.036639426
C19orf29	713	20	0.9011	68	0.00824	12	0.99801	3	0.99998	49	0.999236729
MAP3K8	710	9	0.99997	25	0.99999	5	1	2	1	105	4.16E-05
NCLN	697	11	0.99963	46	0.73226	7	0.99999	1	1	38	0.999997914
B4GALT7	695	17	0.9678	31	0.99885	10	0.99953	3	0.99997	50	0.997515856
YOD1	688	11	0.99954	41	0.90517	9	0.99981	2	1	157	1.82E-23
FKBP7	686	18	0.93956	32	0.99733	17	0.92528	18	0.20871	54	0.983616344
FIP1L1	684	16	0.97781	27	0.99987	6	1	2	1	48	0.998463302
BRD9	681	10	0.9998	35	0.98692	4	1	3	0.99996	24	1
ERGIC2	679	4	1	15	1	0	1	0	1	29	0.999999997
TMEM42	649	14	0.988	26	0.99975	4	1	3	0.99992	190	2.14E-44
FLI1	639	18	0.88629	88	1.33E-09	14	0.96853	7	0.98406	155	4.01E-26
MVD	638	17	0.92467	53	0.13585	7	0.99994	1	1	26	0.999999998
NEK8	635	6	1	18	1	2	1	1	1	41	0.999651341
DDX59	628	6	0.99999	12	1	4	1	2	0.99998	41	0.99952063
AXIN1	626	17	0.91109	64	0.00216	5	1	0	1	54	0.9133209

EFNA3	626	6	0.99999	7	1	0	1	0	1	31	0.999999449
REV3L	606	5	1	7	1	1	1	0	1	38	0.999757962
MUTED	600	25	0.25126	43	0.50928	8	0.99948	2	0.99997	41	0.998374305
RBM8A	600	2	1	20	0.99998	8	0.99948	2	0.99997	23	0.999999999
ZC3H18	600	14	0.97119	69	5.09E-05	5	0.99999	2	0.99997	42	0.997353357
PCYT2	595	15	0.94584	32	0.96623	5	0.99999	2	0.99997	25	0.999999984
EXOC8	580	11	0.99467	16	1	1	1	0	1	50	0.906122521
MAP3K7	579	12	0.9884	25	0.99837	7	0.99972	5	0.99483	50	0.903791973
AASDHPPT	576	11	0.99419	19	0.99998	5	0.99998	1	1	34	0.999910841
USP46	574	11	0.99394	14	1	1	1	1	1	47	0.953600837
UCKL1	569	12	0.98591	23	0.99937	4	1	2	0.99995	40	0.996419637
TMC6	557	2	1	18	0.99998	10	0.99115	4	0.99786	33	0.999876453
CCNA1	556	7	0.99983	8	1	5	0.99997	4	0.99782	32	0.999933311
CSDE1	543	7	0.99975	19	0.99991	5	0.99995	2	0.99991	28	0.999992858
AASS	532	4	1	11	1	2	1	1	0.99999	30	0.999939485
CGGBP1	532	14	0.91458	38	0.52016	6	0.99973	1	0.99999	31	0.99987934
DNAH3	531	10	0.99323	17	0.99998	12	0.94559	3	0.99923	56	0.396753538
MRPL12	531	10	0.99323	47	0.07217	1	1	0	1	34	0.999180295
PPP1CA	531	5	0.99998	14	1	2	1	1	0.99999	69	0.018320497
SPHK2	530	8	0.99892	20	0.99967	3	1	1	0.99999	23	0.99999981
IFIH1	521	14	0.89972	33	0.78712	2	1	0	1	22	0.99999987
WT1-AS	519	16	0.77213	41	0.26701	16	0.6691	21	0.00368	56	0.329097054
RUNX1T1	516	21	0.31103	93	3.94E-17	22	0.14693	9	0.7714	96	2.32E-09
HAS2	515	6	0.99985	18	0.99988	2	1	0	1	33	0.999023449
SLC4A2	514	5	0.99996	22	0.99756	1	1	0	1	15	1
MEN1	510	11	0.97804	19	0.99966	2	1	1	0.99999	131	1.33E-24
C16orf70	508	5	0.99996	6	1	1	1	0	1	68	0.010267787
EZR	506	5	0.99995	7	1	1	1	2	0.9998	42	0.932042305
COQ5	505	1	1	4	1	1	1	0	1	95	1.56E-09

WDR83	504	14	0.8727	42	0.16307	5	0.99986	0	1	22	0.999999584
C9orf142	503	7	0.99928	12	1	4	0.99997	2	0.99979	76	0.000242781
TUBGCP2	503	10	0.98777	43	0.12244	4	0.99997	1	0.99998	31	0.999470245
C2orf3	496	6	0.99974	13	1	4	0.99996	0	1	28	0.999895136
DOT1L	496	13	0.91015	55	0.0007	12	0.90793	7	0.90607	72	0.001097916
HENMT1	494	11	0.97032	20	0.99865	7	0.9978	4	0.99382	62	0.044781579
MATR3	491	4	0.99998	7	1	1	1	0	1	39	0.960373543
RPH3AL	491	7	0.99901	27	0.93971	8	0.9937	3	0.99839	20	0.999999866
TMCO6	491	3	1	6	1	0	1	2	0.99973	58	0.121322222
EXOSC8	487	6	0.99967	20	0.99825	7	0.9974	6	0.94918	21	0.999999508
ASCL2	486	4	0.99998	24	0.98138	2	1	1	0.99998	45	0.763913254
C13orf23	485	2	1	10	1	2	1	0	1	8	1
TRAP1	483	6	0.99963	5	1	1	1	0	1	69	0.002087554
HNRNPF	479	9	0.99084	9	1	2	1	0	1	68	0.002619685
IFT140	478	11	0.96024	39	0.20964	5	0.99971	2	0.99965	26	0.999933443
EOMES	477	3	1	11	1	1	1	0	1	99	2.03E-12
GOLGA5	474	10	0.97802	31	0.71947	1	1	0	1	30	0.998813877
MYCBP2	473	17	0.53709	29	0.82793	29	0.00131	16	0.04565	33	0.993772414
BRAT1	472	7	0.99838	11	1	3	0.99999	1	0.99997	98	2.57E-12
BCL3	468	7	0.99821	21	0.9935	17	0.40024	5	0.97248	48	0.488576399
HOXA7	462	9	0.98679	15	0.99991	10	0.94945	11	0.39233	29	0.998834346
DLEU1	461	11	0.94631	32	0.59004	7	0.99528	2	0.99951	145	1.00E-37
RG9MTD1	461	2	1	5	1	1	1	0	1	38	0.929337507
GALNS	458	5	0.9998	40	0.10561	9	0.97236	1	0.99995	22	0.999991473
UAP1L1	458	5	0.9998	13	0.99998	0	1	0	1	69	0.000500337
HMGN4	454	10	0.96762	44	0.02317	6	0.99808	1	0.99995	74	2.48E-05
RNH1	453	12	0.8949	27	0.85982	20	0.12718	6	0.92233	59	0.026386633
FAT4	447	15	0.64629	45	0.0119	4	0.99984	1	0.99994	41	0.775533422
EXOC5	444	4	0.99993	13	0.99997	1	1	1	0.99994	23	0.999953076

KEAP1	444	8	0.99152	15	0.99979	6	0.99755	3	0.99622	14	0.999999996
EIF2AK1	442	4	0.99993	8	1	2	1	1	0.99993	62	0.005156286
GPR108	439	8	0.99051	17	0.99869	1	1	0	1	30	0.994177904
HEATR2	437	15	0.61149	53	9.74E-05	3	0.99996	0	1	20	0.999994628
SLC7A7	437	4	0.99992	11	1	1	1	1	0.99993	46	0.415834206
MIPOL1	435	2	1	3	1	0	1	0	1	27	0.998704952
MRPL38	434	13	0.78859	48	0.00158	3	0.99996	0	1	28	0.997539399
NDUFS7	432	9	0.97538	30	0.58783	4	0.99976	2	0.99912	26	0.999198366
JMJD8	430	10	0.94946	31	0.50155	1	1	0	1	33	0.967824526
DNAJC2	429	4	0.99989	11	0.99999	5	0.99892	1	0.99991	19	0.999996475
ETF1	429	3	0.99998	12	0.99998	0	1	2	0.99907	33	0.966683994
NCSTN	428	6	0.99834	18	0.99594	9	0.9524	1	0.99991	75	1.51E-06
PPIL6	428	2	1	7	1	5	0.99889	0	1	10	1
KHSRP	427	19	0.20033	62	5.76E-08	15	0.46201	10	0.42138	59	0.008546646
