# Supplementary material for: Integrative Analysis of DNA Methylation and Gene Expression Data Identifies EPAS1 as a Key Regulator of COPD
Source: PLoS Genet. 2015 Jan 8;11(1):e1004898. doi: 10.1371/journal.pgen.1004898 (PMC4287352; doi:10.1371/journal.pgen.1004898)
Supplement: S12 Table — Overlap between downstream genes of key regulators in COPD lung tissues and COPD emphysema signature in mouse. (PDF) [file pgen.1004898.s021.pdf]

STable 12. Overlap between downstream genes of key regulators in COPD lung tissues and COPD emphysema signature in mouse

Regulator	Downstream	Emphysema signature (mouse)			
		p<=0.01	p(overlap_pvalue)	fdr<0.1	p(overlap_fdr)
GAK	1817	49	0.980436872	4	0.999051198
ACSF3	1549	47	0.884793869	3	0.999074349
CLCN7	1506	30	0.999899626	0	1
ALG12	1357	26	0.999822006	2	0.99931868
ABHD14B	1300	43	0.68673695	5	0.953612993
SCRIB	1255	20	0.999991735	1	0.999884368
SSNA1	1224	23	0.999901038	2	0.998801838
HIST1H2BG	1190	36	0.836235052	2	0.998091436
SELO	1131	19	0.999967904	0	1
ZFYVE26	1126	38	0.609403571	2	0.997111786
THOP1	1124	21	0.999694854	1	0.999692321
C6orf226	1080	33	0.82181731	4	0.947276465
SRI	1005	23	0.993774536	1	0.999380205
DAGLB	1004	24	0.985706635	1	0.999287189
MED16	1004	23	0.992894941	1	0.999341259
TSGA10	1001	16	0.999938891	0	1
C13orf27	986	34	0.563633798	2	0.992993335
SEC16A	974	10	0.99999984	2	0.992067223
PMPCA	966	24	0.975848094	2	0.992308529
DEDD2	949	36	0.363860207	4	0.90707129
SETBP1	899	22	0.976624367	2	0.988309248
DNAJA1	884	24	0.91548546	1	0.998088061
ADRM1	858	17	0.996764666	1	0.997677508
MATK	840	20	0.977227351	1	0.997529827
NUDT16L1	827	18	0.987584308	1	0.996888265
PLXNB2	826	24	0.866559701	2	0.981512441
COASY	814	19	0.980112001	1	0.997017536
JMJD1C	811	18	0.990384133	0	1
HINT2	804	28	0.590716276	4	0.828314398
EPAS1	779	105	2.15E-34	30	5.07E-15
NADSYN1	765	13	0.999054996	0	1
NCAPD2	753	24	0.701921727	1	0.995172691
MOBKL2A	751	15	0.996209323	0	1
PAX9	751	14	0.996056834	0	1
C3orf26	750	17	0.981820786	0	1
ATAD1	745	27	0.51634613	0	1
RPS6	742	10	0.999902031	0	1

TMEM102	741	16	0.989885643	1	0.995189156
FOXK2	739	12	0.999485786	1	0.994987756
C19orf29	713	14	0.99420352	2	0.959367079
MAP3K8	710	20	0.885754151	1	0.994085843
NCLN	697	17	0.967090939	0	1
B4GALT7	695	21	0.761941851	4	0.702046225
YOD1	688	32	0.057678572	3	0.858133821
FKBP7	686	26	0.443733392	4	0.733795216
FIP1L1	684	14	0.992097475	0	1
BRD9	681	17	0.94460706	1	0.991906259
ERGIC2	679	19	0.89740322	0	1
TMEM42	649	21	0.677626219	1	0.990062051
FLI1	639	14	0.980805078	1	0.98939161
MVD	638	16	0.953346094	0	1
NEK8	635	19	0.816477153	0	1
DDX59	628	17	0.907325322	0	1
AXIN1	626	12	0.994431125	0	1
EFNA3	626	21	0.586759933	4	0.624190518
REV3L	606	19	0.734881583	3	0.800497961
MUTED	600	14	0.961114791	0	1
RBM8A	600	10	0.997553054	1	0.98500976
ZC3H18	600	10	0.997852202	0	1
PCYT2	595	20	0.639732277	2	0.926540889
EXOC8	580	18	0.710390703	1	0.982502775
MAP3K7	579	10	0.997488651	0	1
AASDHPPT	576	12	0.986739249	0	1
USP46	574	7	0.999812022	0	1
UCKL1	569	15	0.883356529	2	0.902576355
TMC6	557	18	0.656756373	0	1
CCNA1	556	9	0.995722953	1	0.975965997
CSDE1	543	11	0.985782869	0	1
AASS	532	11	0.980991597	0	1
CGGBP1	532	9	0.995003702	0	1
DNAH3	531	11	0.975832912	1	0.974493003
MRPL12	531	7	0.999396634	0	1
PPP1CA	531	15	0.847144955	0	1
SPHK2	530	10	0.992205704	0	1
IFIH1	521	10	0.990312664	1	0.976160362
WT1-AS	519	27	0.027533301	1	0.975011662
RUNX1T1	516	12	0.95631546	2	0.880426946
HAS2	515	12	0.962726247	0	1

SLC4A2	514	11	0.982212618	0	1
MEN1	510	18	0.532508667	3	0.695520097
C16orf70	508	18	0.512531955	2	0.871315736
EZR	506	12	0.932221017	0	1
COQ5	505	21	0.212929521	4	0.451196726
WDR83	504	6	0.999657363	0	1
C9orf142	503	7	0.998441014	0	1
TUBGCP2	503	10	0.986501806	1	0.973180845
C2orf3	496	13	0.86988488	2	0.852683804
DOT1L	496	12	0.941653426	0	1
HENMT1	494	18	0.532508667	5	0.283524393
MATR3	491	11	0.962351186	0	1
RPH3AL	491	13	0.856902609	1	0.965470062
TMCO6	491	18	0.402754392	3	0.645423339
EXOSC8	487	18	0.508513123	1	0.972200549
ASCL2	486	14	0.839098101	1	0.970796858
C13orf23	485	8	0.99624407	0	1
TRAP1	483	8	0.994386198	1	0.964932808
HNRNPF	479	10	0.971463275	0	1
IFT140	478	11	0.942567681	1	0.963708005
EOMES	477	20	0.227096653	1	0.965114385
GOLGA5	474	13	0.876018582	0	1
MYCBP2	473	26	0.019720846	3	0.654816514
BRAT1	472	14	0.783081101	1	0.965170056
BCL3	468	12	0.892175285	1	0.962145162
HOXA7	462	13	0.797775846	2	0.822703628
DLEU1	461	21	0.143514545	3	0.631886331
RG9MTD1	461	13	0.811937294	2	0.8282317
GALNS	458	7	0.996520292	0	1
UAP1L1	458	15	0.616822436	1	0.958461001
HMGN4	454	7	0.995522657	1	0.956042821
RNH1	453	10	0.956172286	0	1
FAT4	447	13	0.80916435	1	0.959511596
EXOC5	444	15	0.608730903	0	1
KEAP1	444	6	0.998843417	0	1
EIF2AK1	442	16	0.478684046	1	0.955574813
GPR108	439	11	0.914342886	0	1
HEATR2	437	9	0.973726868	1	0.955278884
SLC7A7	437	14	0.674266017	1	0.954468621
MIPOL1	435	17	0.339772817	1	0.952091988
MRPL38	434	9	0.971217363	0	1

NDUFS7	432	11	0.893515347	2	0.804011333
JMJD8	430	6	0.997465409	0	1
DNAJC2	429	9	0.969882938	1	0.952977178
ETF1	429	13	0.760735149	1	0.953712095
NCSTN	428	9	0.967776364	0	1
PPIL6	428	9	0.948937469	1	0.942537051
KHSRP	427	11	0.904398625	1	0.954502046
