# Supplementary material for: Integrative Analysis of DNA Methylation and Gene Expression Data Identifies EPAS1 as a Key Regulator of COPD
Source: PLoS Genet. 2015 Jan 8;11(1):e1004898. doi: 10.1371/journal.pgen.1004898 (PMC4287352; doi:10.1371/journal.pgen.1004898)
Supplement: S13 Table — GO enrichment analysis of EPAS1 downstream genes in COPD. (PDF) [file pgen.1004898.s022.pdf]

STable 13. GO enrichment analysis of *EPAS1* downstream genes in COPD

GOBPID	Pvalue	OddsRatio	Overlap	Size	Term
GO:0048646	1.17E-06	1.72247338	112	1324	anatomical structure formation involved in morphogenesis
GO:0034333	3.53E-06	6.38268398	12	45	adherens junction assembly
GO:0003018	4.09E-06	3.8574196	19	106	vascular process in circulatory system
GO:0040011	5.92E-06	1.73892677	91	1053	locomotion
GO:0072358	7.54E-06	1.94872916	61	628	cardiovascular system development
GO:0072359	7.54E-06	1.94872916	61	628	circulatory system development
GO:0048514	8.29E-06	2.27426941	41	364	blood vessel morphogenesis
GO:0001525	1.22E-05	2.37185342	36	307	angiogenesis
GO:0001568	1.34E-05	2.15609408	44	410	blood vessel development
GO:0007044	1.44E-05	5.3978022	12	51	cell-substrate junction assembly
GO:0051301	1.52E-05	2.1439763	44	412	cell division
GO:0009117	1.80E-05	1.86630259	63	674	nucleotide metabolic process
GO:0006928	1.86E-05	1.70935765	85	994	cellular component movement
GO:0048585	2.08E-05	1.88543254	60	635	negative regulation of response to stimulus
GO:0001952	2.19E-05	5.13356562	12	53	regulation of cell-matrix adhesion
GO:0006753	2.26E-05	1.85027084	63	679	nucleoside phosphate metabolic process
GO:0023057	2.82E-05	1.94260699	53	544	negative regulation of signaling
GO:0044087	2.85E-05	2.329542	34	294	regulation of cellular component biogenesis
GO:0016477	2.89E-05	1.81546473	65	713	cell migration
GO:0034330	2.91E-05	2.88557385	23	164	cell junction organization
GO:0045216	3.20E-05	3.04042924	21	143	cell-cell junction organization
GO:0010648	3.27E-05	1.93026333	53	547	negative regulation of cell communication
GO:0048041	3.36E-05	6.03557399	10	39	focal adhesion assembly
GO:0051893	3.62E-05	8.21970681	8	25	regulation of focal adhesion assembly
GO:0090109	3.62E-05	8.21970681	8	25	regulation of cell-substrate junction assembly
GO:1901888	3.74E-05	6.84195343	9	32	regulation of cell junction assembly
GO:0034332	4.02E-05	4.09856688	14	74	adherens junction organization

GO:0001944	4.57E-05	2.03510409	44	431	vasculature development
GO:0016192	6.45E-05	1.76054087	65	732	vesicle-mediated transport
GO:0055086	6.58E-05	1.76705501	64	718	nucleobase-containing small molecule metabolic process
GO:0009966	6.78E-05	1.54430358	112	1447	regulation of signal transduction
GO:0071822	7.81E-05	1.68727079	74	869	protein complex subunit organization
GO:0007160	8.04E-05	3.01678886	19	130	cell-matrix adhesion
GO:0034329	8.94E-05	2.89099151	20	142	cell junction assembly
GO:0035024	9.67E-05	17.4034537	5	10	negative regulation of Rho protein signal transduction
GO:0009968	9.78E-05	1.88824457	49	514	negative regulation of signal transduction
GO:0009150	0.00010261	1.88401523	49	515	purine ribonucleotide metabolic process
GO:0009259	0.00010785	1.86708193	50	530	ribonucleotide metabolic process
GO:0010810	0.00011354	3.46732561	15	91	regulation of cell-substrate adhesion
GO:0048870	0.00011571	1.7062464	67	776	cell motility
GO:0051674	0.00011571	1.7062464	67	776	localization of cell
GO:0051174	0.000117	1.61251717	84	1031	regulation of phosphorus metabolic process
GO:0051056	0.00011981	2.2026687	32	290	regulation of small GTPase mediated signal transduction
GO:0007200	0.00012517	4.99819168	10	45	phospholipase C-activating G-protein coupled receptor signaling pathway
GO:0019693	0.00013602	1.84696294	50	535	ribose phosphate metabolic process
GO:0019220	0.00014364	1.60528557	83	1022	regulation of phosphate metabolic process
GO:0007264	0.00016123	1.96026283	41	414	small GTPase mediated signal transduction
GO:0046580	0.00016124	7.63041339	7	23	negative regulation of Ras protein signal transduction
GO:0044057	0.00018454	2.00151848	38	376	regulation of system process
GO:0007266	0.00018658	3.29276316	15	95	Rho protein signal transduction
GO:0003008	0.00019664	1.51047043	106	1390	system process
GO:0044085	0.00020214	1.50914107	106	1391	cellular component biogenesis

GO:0051058	0.00021698	7.18091709	7	24	negative regulation of small GTPase mediated signal transduction
GO:0042310	0.00022122	4.60234843	10	48	vasoconstriction
GO:0045765	0.00024674	2.82769574	18	130	regulation of angiogenesis
GO:0007010	0.00027017	1.7123682	58	666	cytoskeleton organization
GO:0060841	0.00027714	12.4287957	5	12	venous blood vessel development
GO:0006468	0.0003106	1.59139169	76	939	protein phosphorylation
GO:0008277	0.00031548	3.2743949	14	89	regulation of G-protein coupled receptor protein signaling pathway
GO:0022607	0.00034232	1.50775233	97	1268	cellular component assembly
GO:0030155	0.00034946	2.25253056	26	230	regulation of cell adhesion
GO:0050880	0.00035522	3.23101743	14	90	regulation of blood vessel size
GO:0006163	0.00035714	1.76315369	50	557	purine nucleotide metabolic process
GO:0042391	0.00038898	2.27593611	25	219	regulation of membrane potential
GO:0035150	0.00039916	3.18876665	14	91	regulation of tube size
GO:0070482	0.00041247	2.35880264	23	195	response to oxygen levels
GO:0090066	0.00045683	2.20840097	26	234	regulation of anatomical structure size
GO:0030334	0.00045768	1.97758844	34	339	regulation of cell migration
GO:0001932	0.00059349	1.65188274	58	687	regulation of protein phosphorylation
GO:0007155	0.00061904	1.60925824	64	778	cell adhesion
GO:0022610	0.00066203	1.60445381	64	780	biological adhesion
GO:0072521	0.00066717	1.69932381	51	587	purine-containing compound metabolic process
GO:0010562	0.00067057	1.71787422	49	558	positive regulation of phosphorus metabolic process
GO:0045937	0.00067057	1.71787422	49	558	positive regulation of phosphate metabolic process
GO:1901342	0.00067181	2.57223265	18	141	regulation of vasculature development
GO:0010632	0.00070103	6.96603774	6	21	regulation of epithelial cell migration
GO:0032879	0.00072846	1.49746702	87	1138	regulation of localization

GO:0019226	0.00073011	1.68271923	52	604	transmission of nerve impulse
GO:2000026	0.0007469	1.54967959	73	921	regulation of multicellular organismal development
GO:0031589	0.00075529	2.2932239	22	191	cell-substrate adhesion
GO:0030029	0.00075546	1.86035165	37	390	actin filament-based process
GO:0090130	0.00077045	5.54638511	7	29	tissue migration
GO:0016310	0.00077816	1.51075437	82	1062	phosphorylation
GO:0031644	0.00083581	2.32503874	21	180	regulation of neurological system process
GO:0050878	0.00083962	1.76273436	43	477	regulation of body fluid levels
GO:0009152	0.00085452	2.37746589	20	168	purine ribonucleotide biosynthetic process
GO:0035637	0.00085616	1.66089715	53	623	multicellular organismal signaling
GO:0050678	0.00086448	2.43847172	19	156	regulation of epithelial cell proliferation
GO:0007188	0.00090587	3.23106227	12	77	adenylate cyclase-modulating G-protein coupled receptor signaling pathway
GO:0043114	0.00091979	6.53007075	6	22	regulation of vascular permeability
GO:0009260	0.0009648	2.29573619	21	182	ribonucleotide biosynthetic process
GO:1901068	0.00098775	1.99431259	29	286	guanosine-containing compound metabolic process
GO:0031577	0.001045	4.50188257	8	39	spindle checkpoint
GO:0051017	0.00106916	3.37459338	11	68	actin filament bundle assembly
GO:0040012	0.00109747	1.83390236	36	384	regulation of locomotion
GO:0009165	0.00109963	2.09944432	25	235	nucleotide biosynthetic process
GO:0003013	0.00110186	1.97854718	29	288	circulatory system process
GO:0008015	0.00110186	1.97854718	29	288	blood circulation
GO:0001666	0.00111057	2.26715272	21	184	response to hypoxia
GO:0042325	0.0011419	1.58833038	60	736	regulation of phosphorylation
GO:1901293	0.00116823	2.08930231	25	236	nucleoside phosphate biosynthetic process
GO:0036293	0.00119029	2.25312242	21	185	response to decreased oxygen levels
GO:0007051	0.0012105	3.31611017	11	69	spindle organization
GO:0002040	0.00124472	4.36080442	8	40	sprouting angiogenesis

GO:2000145	0.0012571	1.85244939	34	359	regulation of cell motility
GO:0046390	0.00127486	2.23926219	21	186	ribose phosphate biosynthetic process
GO:0045785	0.00127731	2.78738419	14	102	positive regulation of cell adhesion
GO:0006164	0.00132357	2.28358458	20	174	purine nucleotide biosynthetic process
GO:0003085	0.00143052	11.5830721	4	10	negative regulation of systemic arterial blood pressure
GO:0051239	0.00143447	1.40972424	107	1485	regulation of multicellular organismal process
GO:0006898	0.00154497	2.72494692	14	104	receptor-mediated endocytosis
GO:0045859	0.00157566	1.68897043	44	507	regulation of protein kinase activity
GO:0035295	0.00158665	1.84168078	33	350	tube development
GO:0046578	0.00158912	2.10922355	23	215	regulation of Ras protein signal transduction
GO:0001945	0.00172259	7.24686028	5	17	lymph vessel development
GO:0043149	0.00173479	4.10354426	8	42	stress fiber assembly
GO:0051640	0.00177196	2.40566452	17	141	organelle localization
GO:0007599	0.00178243	1.76221008	37	409	hemostasis
GO:0042060	0.00181066	1.68578978	43	496	wound healing
GO:0046903	0.00185486	1.59583066	53	645	secretion
GO:0007015	0.00186775	2.2107626	20	179	actin filament organization
GO:0009888	0.00187006	1.46741372	79	1047	tissue development
GO:0007187	0.00203293	2.63632628	14	107	G-protein coupled receptor signaling pathway, coupled to cyclic nucleotide second messenger
GO:0051270	0.00204936	1.76068049	36	398	regulation of cellular component movement
GO:0010595	0.00209324	4.51723535	7	34	positive regulation of endothelial cell migration
GO:0031503	0.00215123	9.92745186	4	11	protein complex localization
GO:0007186	0.00217398	1.70080547	40	457	G-protein coupled receptor signaling pathway
GO:0019637	0.00225553	1.46932257	75	991	organophosphate metabolic process
GO:0001763	0.00232461	2.27282789	18	157	morphogenesis of a branching structure
GO:0048729	0.00232787	1.74572087	36	401	tissue morphogenesis
GO:0030111	0.00239634	2.3296375	17	145	regulation of Wnt receptor signaling pathway

GO:0006140	0.00244285	1.91160741	27	276	regulation of nucleotide metabolic process
GO:0050877	0.00258584	1.45680439	76	1012	neurological system process
GO:0000904	0.0026036	1.61231303	47	565	cell morphogenesis involved in differentiation
GO:0043549	0.00267649	1.62728101	45	536	regulation of kinase activity
GO:0001824	0.00274244	3.76980135	8	45	blastocyst development
GO:0031623	0.00274244	3.76980135	8	45	receptor internalization
GO:0007596	0.00274966	1.7261531	36	405	blood coagulation
GO:0032940	0.00279007	1.60580802	47	567	secretion by cell
GO:0046039	0.00284415	1.88832753	27	279	GTP metabolic process
GO:0055001	0.00284526	2.35659248	16	135	muscle cell development
GO:0008283	0.00285353	1.40494915	92	1272	cell proliferation
GO:0030036	0.00288009	1.78294461	32	349	actin cytoskeleton organization
GO:0003300	0.00288916	4.97304582	6	27	cardiac muscle hypertrophy
GO:0010631	0.00288916	4.97304582	6	27	epithelial cell migration
GO:0014897	0.00288916	4.97304582	6	27	striated muscle hypertrophy
GO:0090132	0.00288916	4.97304582	6	27	epithelium migration
GO:0031399	0.00292089	1.48291218	67	875	regulation of protein modification process
GO:1901657	0.00305237	1.63310013	43	510	glycosyl compound metabolic process
GO:0036303	0.00308828	8.68573668	4	12	lymph vessel morphogenesis
GO:0050817	0.00310769	1.71175343	36	408	coagulation
GO:0043933	0.00311984	1.43171908	80	1083	macromolecular complex subunit organization
GO:0050804	0.00319463	2.25821818	17	149	regulation of synaptic transmission
GO:0006813	0.00322261	2.72455164	12	89	potassium ion transport
GO:0006897	0.00332296	1.84163061	28	296	endocytosis
GO:0050673	0.00332668	2.09060634	20	188	epithelial cell proliferation
GO:0072522	0.00332668	2.09060634	20	188	purine-containing compound biosynthetic process
GO:0030879	0.00335088	2.5841704	13	101	mammary gland development
GO:0048754	0.00337183	2.38821227	15	125	branching morphogenesis of an epithelial tube
GO:0042327	0.00337816	1.63256579	42	498	positive regulation of phosphorylation

GO:0071560	0.00350122	2.17758621	18	163	cellular response to transforming growth factor beta stimulus
GO:0009116	0.00350194	1.62884026	42	499	nucleoside metabolic process
GO:0014896	0.00350992	4.74656947	6	28	muscle hypertrophy
GO:0032091	0.00350992	4.74656947	6	28	negative regulation of protein binding
GO:0050999	0.00350992	4.74656947	6	28	regulation of nitric-oxide synthase activity
GO:0001936	0.00353495	3.27103081	9	57	regulation of endothelial cell proliferation
GO:0007589	0.00353495	3.27103081	9	57	body fluid secretion
GO:1901069	0.0036049	1.87634394	26	270	guanosine-containing compound catabolic process
GO:0016044	0.00361684	1.77035895	31	340	cellular membrane organization
GO:0009119	0.00367066	1.65553527	39	456	ribonucleoside metabolic process
GO:0050808	0.00368042	2.45022293	14	114	synapse organization
GO:0055002	0.00368042	2.45022293	14	114	striated muscle cell development
GO:0071559	0.0037386	2.16247366	18	164	response to transforming growth factor beta stimulus
GO:0001954	0.00376551	5.79591837	5	20	positive regulation of cell-matrix adhesion
GO:0006760	0.00376551	5.79591837	5	20	folic acid-containing compound metabolic process
GO:0060562	0.00377488	1.95368875	23	230	epithelial tube morphogenesis
GO:0001934	0.00387578	1.62824664	41	487	positive regulation of protein phosphorylation
GO:0032989	0.00390033	1.4790769	63	823	cellular component morphogenesis
GO:0001508	0.00392547	2.34513841	15	127	regulation of action potential
GO:0007265	0.00393811	2.01568068	21	204	Ras protein signal transduction
GO:0008361	0.00398626	3.20398491	9	58	regulation of cell size
GO:0002009	0.0040022	1.79373229	29	314	morphogenesis of an epithelium
GO:0006956	0.00406277	3.93294387	7	38	complement activation
GO:0019229	0.00406277	3.93294387	7	38	regulation of vasoconstriction
GO:0001935	0.00409595	2.95859258	10	69	endothelial cell proliferation

GO:0032412	0.00409595	2.95859258	10	69	regulation of ion transmembrane transporter activity
GO:0016525	0.00415726	3.48611987	8	48	negative regulation of angiogenesis
GO:0043154	0.00415726	3.48611987	8	48	negative regulation of cysteine-type endopeptidase activity involved in apoptotic process
GO:0060389	0.00422456	4.53978671	6	29	pathway-restricted SMAD protein phosphorylation
GO:0048207	0.00426967	7.7199582	4	13	vesicle targeting, rough ER to cis-Golgi
GO:0048208	0.00426967	7.7199582	4	13	COPII vesicle coating
GO:0090114	0.00426967	7.7199582	4	13	COPII-coated vesicle budding
GO:0061024	0.00430022	1.74708617	31	344	membrane organization
GO:0022603	0.00431351	1.61691168	41	490	regulation of anatomical structure morphogenesis
GO:0032956	0.00449057	2.17480876	17	154	regulation of actin cytoskeleton organization
GO:0046128	0.00449093	1.65603933	37	432	purine ribonucleoside metabolic process
GO:0070848	0.00453552	1.70824726	33	374	response to growth factor stimulus
GO:0010517	0.0045508	2.90901899	10	70	regulation of phospholipase activity
GO:0002274	0.00463913	2.58906526	12	93	myeloid leukocyte activation
GO:0050679	0.00463913	2.58906526	12	93	positive regulation of epithelial cell proliferation
GO:0001938	0.00472291	3.80969488	7	39	positive regulation of endothelial cell proliferation
GO:0021846	0.00472315	5.43318289	5	21	cell proliferation in forebrain
GO:0043266	0.00472315	5.43318289	5	21	regulation of potassium ion transport
GO:0007202	0.00473604	3.4007848	8	49	activation of phospholipase C activity
GO:0071363	0.00480673	1.7159998	32	361	cellular response to growth factor stimulus
GO:0007167	0.00487467	1.49586447	56	722	enzyme linked receptor protein signaling pathway
GO:0051338	0.00494666	1.56706914	45	554	regulation of transferase activity

GO:0042278	0.00502293	1.64309564	37	435	purine nucleoside metabolic process
GO:0050793	0.00503184	1.3852828	86	1200	regulation of developmental process
GO:0007528	0.00504116	4.35023585	6	30	neuromuscular junction development
GO:0000279	0.00506697	1.63072237	38	450	M phase
GO:0051969	0.00513406	2.0899134	18	169	regulation of transmission of nerve impulse
GO:0006461	0.00513605	1.51434287	52	662	protein complex assembly
GO:0050790	0.00525952	1.38252301	86	1202	regulation of catalytic activity
GO:0051259	0.00529233	1.79353889	27	292	protein oligomerization
GO:2000117	0.00537464	3.31951329	8	50	negative regulation of cysteine-type endopeptidase activity
GO:0070271	0.00544887	1.50910602	52	664	protein complex biogenesis
GO:0032231	0.0054607	3.69391553	7	40	regulation of actin filament bundle assembly
GO:0007163	0.00551372	2.52621916	12	95	establishment or maintenance of cell polarity
GO:0090257	0.00551829	2.41790414	13	107	regulation of muscle system process
GO:0022898	0.00557918	2.81466925	10	72	regulation of transmembrane transporter activity
GO:0048588	0.00561435	3.01831936	9	61	developmental cell growth
GO:0060429	0.00568664	1.59708421	40	483	epithelium development
GO:0010611	0.00572187	6.94733542	4	14	regulation of cardiac muscle hypertrophy
GO:0007268	0.00586642	1.5664482	43	529	synaptic transmission
GO:1901135	0.00605225	1.39145781	79	1095	carbohydrate derivative metabolic process
GO:0009719	0.00605922	1.43333841	66	887	response to endogenous stimulus
GO:0045446	0.00628156	3.58494673	7	41	endothelial cell differentiation
GO:0002682	0.00637873	1.47845271	55	716	regulation of immune system process
GO:0006184	0.00638572	1.80883206	25	268	GTP catabolic process
GO:0051128	0.00638653	1.39630083	76	1049	regulation of cellular component organization
GO:0042127	0.00642088	1.4113929	71	969	regulation of cell proliferation
GO:0032268	0.00649846	1.38945283	78	1082	regulation of cellular protein metabolic process
GO:0051246	0.00692187	1.36298904	87	1231	regulation of protein metabolic process

GO:0010594	0.00695669	2.90600316	9	63	regulation of endothelial cell migration
GO:0051100	0.00695669	2.90600316	9	63	negative regulation of binding
GO:0035239	0.00698142	1.84460132	23	242	tube morphogenesis
GO:0009199	0.00698175	1.65317118	33	385	ribonucleoside triphosphate metabolic process
GO:0045931	0.00701265	4.01487663	6	32	positive regulation of mitotic cell cycle
GO:0009141	0.00706099	1.63867019	34	400	nucleoside triphosphate metabolic process
GO:0019725	0.00745554	1.5054617	48	613	cellular homeostasis
GO:0051098	0.0074646	2.10583968	16	149	regulation of binding
GO:0014743	0.00746951	6.31518951	4	15	regulation of muscle hypertrophy
GO:0046653	0.00746951	6.31518951	4	15	tetrahydrofolate metabolic process
GO:0051930	0.00746951	6.31518951	4	15	regulation of sensory perception of pain
GO:0051931	0.00746951	6.31518951	4	15	regulation of sensory perception
GO:0070528	0.00746951	6.31518951	4	15	protein kinase C signaling cascade
GO:0060828	0.00765351	2.4091954	12	99	regulation of canonical Wnt receptor signaling pathway
GO:1900542	0.00766928	1.77888306	25	272	regulation of purine nucleotide metabolic process
GO:0010863	0.00768798	3.09737119	8	53	positive regulation of phospholipase C activity
GO:1900274	0.00768798	3.09737119	8	53	regulation of phospholipase C activity
GO:0010518	0.00771371	2.85290823	9	64	positive regulation of phospholipase activity
GO:0050707	0.00771371	2.85290823	9	64	regulation of cytokine secretion
GO:0040017	0.00780732	1.91660956	20	203	positive regulation of locomotion
GO:0048468	0.00790543	1.35679016	86	1221	cell development
GO:0061138	0.00794689	2.08993372	16	150	morphogenesis of a branching epithelium
GO:0000902	0.00812174	1.44340681	58	772	cell morphogenesis
GO:0009124	0.00818366	3.86582809	6	33	nucleoside monophosphate biosynthetic process
GO:0031401	0.0082668	1.48933672	49	632	positive regulation of protein modification process

GO:0030178	0.00833313	2.49354766	11	88	negative regulation of Wnt receptor signaling pathway
GO:0043112	0.00833313	2.49354766	11	88	receptor metabolic process
GO:0048545	0.00835447	1.87003114	21	218	response to steroid hormone stimulus
GO:0000087	0.00841726	1.70449602	28	317	M phase of mitotic cell cycle
GO:0030335	0.00848638	1.93606518	19	191	positive regulation of cell migration
GO:0051648	0.00853195	2.80170955	9	65	vesicle localization
GO:0018208	0.00861454	4.57407254	5	24	peptidyl-proline modification
GO:0042558	0.00861454	4.57407254	5	24	pteridine-containing compound metabolic process
GO:0030168	0.0087082	1.97073317	18	178	platelet activation
GO:0007346	0.00876516	1.75705024	25	275	regulation of mitotic cell cycle
GO:0035023	0.00900617	2.11587436	15	139	regulation of Rho protein signal transduction
GO:0032970	0.00920551	1.95831343	18	179	regulation of actin filament-based process
GO:0031032	0.00929686	3.29338157	7	44	actomyosin structure organization
GO:0048675	0.00929686	3.29338157	7	44	axon extension
GO:0016049	0.00929716	1.70761537	27	305	cell growth
GO:0006182	0.00953511	5.78840125	4	16	cGMP biosynthetic process
GO:0036296	0.00953511	5.78840125	4	16	response to increased oxygen levels
GO:0045907	0.00953511	5.78840125	4	16	positive regulation of vasoconstriction
GO:0055093	0.00953511	5.78840125	4	16	response to hyperoxia
GO:0006936	0.00960085	1.87492477	20	207	muscle contraction
GO:0009205	0.0096603	1.62426418	32	379	purine ribonucleoside triphosphate metabolic process
GO:0006887	0.00969286	1.84147343	21	221	exocytosis
GO:0051302	0.00977672	2.56491996	10	78	regulation of cell division
GO:0048747	0.00982179	2.42997854	11	90	muscle fiber development
