# Supplementary material for: Integrative Analysis of DNA Methylation and Gene Expression Data Identifies EPAS1 as a Key Regulator of COPD
Source: PLoS Genet. 2015 Jan 8;11(1):e1004898. doi: 10.1371/journal.pgen.1004898 (PMC4287352; doi:10.1371/journal.pgen.1004898)
Supplement: S14 Table — Matched methylation and gene expression profile pairs identified by MODMatcher. (PDF) [file pgen.1004898.s023.pdf]

STable 14. Matched methylation and gene expression profile pairs identified by MODMatcher

mRNA	Methylation	Group
LT009615LU	LT009615LU	CTRL
LT010012LU	LT010012LU	CTRL
LT017811RM	LT017811RM	CTRL
LT026501RL	LT026501RL	CTRL
LT028264RL	LT028264RL	CTRL
LT037781RU	LT037781RU	CTRL
LT044225RL	LT044225RL	CTRL
LT045399RL	LT045399RL	CTRL
LT052751LL	LT022271LU	CTRL
LT055745RU	LT055745RU	CTRL
LT058319LU	LT209425LU	CTRL
LT075094LU	LT075094LU	CTRL
LT077800RU	LT077800RU	CTRL
LT079487LL	LT079487LL	CTRL
LT081282RM	LT081282RM	CTRL
LT093297LU	LT093297LU	CTRL
LT102131RL	LT102131RL	CTRL
LT104535RL	LT104535RL	CTRL
LT106997RU	LT106997RU	CTRL
LT115873LU	LT115873LU	CTRL
LT119682RL	LT119682RL	CTRL
LT120371LL	LT120371LL	CTRL
LT121655RL	LT121655RL	CTRL
LT123313RM	LT123313RM	CTRL
LT132955RU	LT132955RU	CTRL
LT139649RU	LT139649RU	CTRL
LT145196LU	LT145196LU	CTRL
LT148286LL	LT148286LL	CTRL
LT156171LL	LT156171LL	CTRL
LT156481LL	LT156481LL	CTRL
LT159753LL	LT159753LL	CTRL
LT163513RL	LT163513RL	CTRL
LT166240RM	LT166240RM	CTRL
LT171169LU	LT171169LU	CTRL
LT173597LL	LT173597LL	CTRL
LT178307RU	LT178307RU	CTRL
LT178986RU	LT178986RU	CTRL
LT180102RL	LT180102RL	CTRL
LT190870RU	LT190870RU	CTRL
LT195484RU	LT195484RU	CTRL

LT197821RL	LT197821RL	CTRL
LT221983RM	LT221983RM	CTRL
LT232107RU	LT232107RU	CTRL
LT241811LL	LT241811LL	CTRL
LT247728RL	LT247728RL	CTRL
LT256221LL	LT256221LL	CTRL
LT256920RU	LT256920RU	CTRL
LT265662LU	LT265662LU	CTRL
LT270821RU	LT270821RU	CTRL
LT272570RL	LT272570RL	CTRL
LT282562RM	LT282562RM	CTRL
LT290677RU	LT290677RU	CTRL
LT001098RU	LT001098RU	COPD
LT002501RL	LT002501RL	COPD
LT007392RU	LT007392RU	COPD
LT009099RL	LT075462RU	COPD
LT010491LL	LT010491LL	COPD
LT017275LL	LT017275LU	COPD
LT020426LU	LT020426LU	COPD
LT024952RU	LT024952RU	COPD
LT024967LU	LT024967LU	COPD
LT025997RU	LT025997RU	COPD
LT026458RL	LT026458RL	COPD
LT028044RU	LT028776LL	COPD
LT028427LU	LT028427LU	COPD
LT030151RU	LT109154RU	COPD
LT032411RU	LT032411RU	COPD
LT032775RU	LT032775RU	COPD
LT037710RU	LT057972LU	COPD
LT038075LL	LT184901RU	COPD
LT038591LU	LT038591LU	COPD
LT042988LU	LT042988RU	COPD
LT043343LU	LT184347RU	COPD
LT043798LU	LT043798LU	COPD
LT046103LU	LT046103LU	COPD
LT050246RU	LT050246RU	COPD
LT051568RU	LT051568RU	COPD
LT051993RU	LT051993RU	COPD
LT059224RL	LT059224RL	COPD
LT059975LU	LT059975LU	COPD
LT070021RU	LT257031RU	COPD
LT076181LI	LT076181LI	COPD
LT076617LL	LT076617LL	COPD

LT080176RU	LT080176RU	COPD
LT083759RL	LT083759RL	COPD
LT083950RU	LT083950RU	COPD
LT084038RM	LT084038RM	COPD
LT084406RU	LT081498RL	COPD
LT085240RU	LT085240RU	COPD
LT089723LL	LT089723LL	COPD
LT095342LU	LT095342LU	COPD
LT100984RL	LT100984RL	COPD
LT108067RU	LT108067RU	COPD
LT112339LI	LT112339LI	COPD
LT112597RU	LT112597RU	COPD
LT113077LL	LT113077LL	COPD
LT115251RU	LT115251RU	COPD
LT118064RL	LT118064RL	COPD
LT122336LU	LT122336LU	COPD
LT123457RU	LT123457RU	COPD
LT126327LU	LT126327LU	COPD
LT132625RU	LT132625RU	COPD
LT137832LU	LT178967RL	COPD
LT140046RU	LT140046RU	COPD
LT140471RU	LT140471RU	COPD
LT145086RU	LT145086RU	COPD
LT147658RU	LT147658RU	COPD
LT150340LU	LT150340LU	COPD
LT150981RU	LT115840LU	COPD
LT151920RL	LT151920RL	COPD
LT152979LL	LT152979LL	COPD
LT154785RU	LT154785RU	COPD
LT158647RU	LT158647RU	COPD
LT160089RU	LT160089RU	COPD
LT161707RU	LT161707RU	COPD
LT174536LU	LT174536LU	COPD
LT176510LU	LT176510LU	COPD
LT178929RL	LT169564RU	COPD
LT184347RU	LT213735RU	COPD
LT190004RU	LT190004RU	COPD
LT192758RU	LT192758RU	COPD
LT194990RU	LT194990RU	COPD
LT196677RU	LT196677RU	COPD
LT197511LU	LT197511LU	COPD
LT198134LU	LT198134LU	COPD
LT200930RL	LT200930RL	COPD

LT203541RU	LT203541RU	COPD
LT208505LU	LT208505LU	COPD
LT212777RU	LT212777RU	COPD
LT215341RU	LT215341RU	COPD
LT220968RU	LT060717LU	COPD
LT229669RU	LT229669RU	COPD
LT233821RL	LT233821RL	COPD
LT234774LU	LT176562LL	COPD
LT235584RU	LT235584RU	COPD
LT242420LU	LT242420LU	COPD
LT244399LU	LT244399LU	COPD
LT245084RU	LT245084RU	COPD
LT249811RU	LT249811RU	COPD
LT253131RU	LT253131RU	COPD
LT261141RU	LT261141RU	COPD
LT262496LU	LT262496LU	COPD
LT263636RU	LT030041RU	COPD
LT271100LU	LT271100LU	COPD
LT271679LU	LT271679LU	COPD
LT273284LL	LT204017RL	COPD
LT282031RU	LT282031RU	COPD
LT282467LL	LT282467LL	COPD
LT284144LU	LT159988LU	COPD
LT286056RU	LT238765RL	COPD
LT294945LU	LT294945RU	COPD
LT295167LU	LT295167LU	COPD
