# Supplementary material for: Integrative Analysis of DNA Methylation and Gene Expression Data Identifies EPAS1 as a Key Regulator of COPD
Source: PLoS Genet. 2015 Jan 8;11(1):e1004898. doi: 10.1371/journal.pgen.1004898 (PMC4287352; doi:10.1371/journal.pgen.1004898)
Supplement: S15 Table — GO enrichment analysis of the 306 genes in the upper left corner of S9A Fig. (PDF) [file pgen.1004898.s024.pdf]

STable 15. GO enrichment analysis of the 306 genes in the upper left corner of Supplementary Figure 9A

GOBPID	Pvalue	OddsRatio	Overlap	Size	Term
GO:0001539	4.42E-13	49.3229167	10	28	ciliary or flagellar motility
GO:0007018	1.96E-10	9.36863137	16	172	microtubule-based movement
GO:0003341	2.87E-09	43.5858896	7	21	cilium movement
GO:0007017	3.65E-08	4.76024351	21	430	microtubule-based process
GO:0006928	3.38E-06	2.70493501	33	1196	cellular component movement
GO:0003351	1.24E-05	38.0562249	4	13	epithelial cilium movement
GO:0001578	2.43E-05	17.209697	5	30	microtubule bundle formation
GO:0016266	4.46E-05	10.5828771	6	55	O-glycan processing
GO:0035082	0.00018278	36.4824636	3	10	axoneme assembly
GO:0007283	0.0001928	3.35074988	14	385	spermatogenesis
GO:0048232	0.00019803	3.34150124	14	386	male gamete generation
GO:0006493	0.00020383	7.84756098	6	72	protein O-linked glycosylation
GO:0009410	0.00026841	5.23159751	8	141	response to xenobiotic stimulus
GO:0006805	0.0012567	4.61967873	7	138	xenobiotic metabolic process
GO:0071466	0.00131043	4.58435583	7	139	cellular response to xenobiotic stimulus
GO:0060271	0.0016011	5.16695122	6	106	cilium morphogenesis
GO:0007276	0.00211593	2.58059758	14	492	gamete generation
GO:0042384	0.00228369	5.87380656	5	78	cilium assembly
GO:0061337	0.00520023	9.44510978	3	30	cardiac conduction
GO:0030317	0.00570802	9.10714286	3	31	sperm motility
GO:0009437	0.00586434	21.1532738	2	10	carnitine metabolic process
GO:0043267	0.00586434	21.1532738	2	10	negative regulation of potassium ion transport
GO:0048870	0.00589604	1.97847068	20	918	cell motility
GO:0051674	0.00589604	1.97847068	20	918	localization of cell
GO:0000226	0.00665751	2.8352795	9	284	microtubule cytoskeleton organization
GO:0010927	0.00774518	3.68022648	6	146	cellular component assembly involved in morphogenesis
GO:0019953	0.00805664	2.19366084	14	573	sexual reproduction

GO:0006641	0.008104	4.27969697	5	105	triglyceride metabolic process
GO:0060306	0.00846873	16.9202381	2	12	regulation of membrane repolarization
GO:0086009	0.00846873	16.9202381	2	12	membrane repolarization
GO:0006639	0.00980236	4.07445887	5	110	acylglycerol metabolic process
GO:0010881	0.00993134	15.3809524	2	13	regulation of cardiac muscle contraction by regulation of the release of sequestered calcium ion
GO:0086019	0.00993134	15.3809524	2	13	cell-cell signaling involved in cardiac conduction
