# Supplementary material for: Integrative Analysis of DNA Methylation and Gene Expression Data Identifies EPAS1 as a Key Regulator of COPD
Source: PLoS Genet. 2015 Jan 8;11(1):e1004898. doi: 10.1371/journal.pgen.1004898 (PMC4287352; doi:10.1371/journal.pgen.1004898)
Supplement: S16 Table — GO enrichment analysis of GAK downstream genes in COPD. (PDF) [file pgen.1004898.s025.pdf]

STable 16. GO enrichment analysis of *GAK* downstream genes in COPD

GOBPID	Pvalue	OddsRatio	Count	Size	Term
GO:0000375	1.09E-06	2.45224313	46	184	RNA splicing, via transesterification reactions
GO:0000377	1.21E-06	2.46972836	45	179	RNA splicing, via transesterification reactions with bulged adenosine as nucleophile
GO:0000398	1.21E-06	2.46972836	45	179	mRNA splicing, via spliceosome
GO:0006397	4.67E-06	1.91545595	71	345	mRNA processing
GO:0008380	6.02E-06	2.00824603	61	285	RNA splicing
GO:0006396	1.90E-05	1.6439089	101	557	RNA processing
GO:0016071	2.05E-05	1.65754414	97	531	mRNA metabolic process
GO:0016568	2.48E-05	1.79544367	72	368	chromatin modification
GO:0043484	0.00011189	3.43674699	17	53	regulation of RNA splicing
GO:0048024	0.00035643	4.43648553	11	29	regulation of mRNA splicing, via spliceosome
GO:0006325	0.00043152	1.5734551	78	443	chromatin organization
GO:0006338	0.00062849	2.50648397	21	82	chromatin remodeling
GO:0050684	0.00100623	3.48474576	12	37	regulation of mRNA processing
GO:0000380	0.00103214	5.27221511	8	19	alternative mRNA splicing, via spliceosome
GO:0030199	0.00114072	4.0303244	10	28	collagen fibril organization
GO:0006890	0.00222256	4.46023835	8	21	retrograde vesicle-mediated transport, Golgi to ER
GO:0006405	0.00348204	2.4213881	16	64	RNA export from nucleus
GO:0060765	0.00366732	4.60994178	7	18	regulation of androgen receptor signaling pathway
GO:0030521	0.00428497	2.54084158	14	54	androgen receptor signaling pathway
GO:0051168	0.00435347	1.9918912	23	107	nuclear export
GO:0051169	0.00475149	1.53886169	52	299	nuclear transport
GO:0006913	0.00578801	1.52717061	51	295	nucleocytoplasmic transport
GO:0000381	0.00606496	4.82700422	6	15	regulation of alternative mRNA splicing, via spliceosome
GO:0034390	0.00650385	6.03127196	5	11	smooth muscle cell apoptotic process
GO:0034391	0.00650385	6.03127196	5	11	regulation of smooth muscle cell apoptotic process
GO:0070647	0.00667386	1.3733062	85	539	protein modification by small protein conjugation or removal
GO:0007160	0.00695234	1.81900856	26	130	cell-matrix adhesion
GO:0010657	0.0075771	3.4094449	8	25	muscle cell apoptotic process

GO:0018205	0.0076979	1.80150805	26	131	peptidyl-lysine modification
GO:0018394	0.00824333	1.93198564	21	100	peptidyl-lysine acetylation
GO:0031214	0.00824333	1.93198564	21	100	biomineral tissue development
GO:0051276	0.00905631	1.34160013	90	582	chromosome organization
GO:0042787	0.0095089	2.35768551	13	53	protein ubiquitination involved in ubiquitin-dependent protein catabolic process
