# Supplementary material for: Integrative Analysis of DNA Methylation and Gene Expression Data Identifies EPAS1 as a Key Regulator of COPD
Source: PLoS Genet. 2015 Jan 8;11(1):e1004898. doi: 10.1371/journal.pgen.1004898 (PMC4287352; doi:10.1371/journal.pgen.1004898)
Supplement: S17 Table — GO enrichment analysis of ETF1 downstream genes in COPD. (PDF) [file pgen.1004898.s026.pdf]

STable 17. GO enrichment analysis of *ETF1* downstream genes in COPD

GOBPID	Pvalue	OddsRatio	Count	Size	Term
GO:0002376	2.43E-06	1.92404116	75	1479	immune system process
GO:0050863	2.65E-06	3.87849668	19	184	regulation of T cell activation
GO:0050776	2.81E-06	2.71012346	32	437	regulation of immune response
GO:0050852	3.22E-06	6.05080214	12	78	T cell receptor signaling pathway
GO:0042110	3.95E-06	3.16712537	24	281	T cell activation
GO:0051249	4.20E-06	3.35105105	22	244	regulation of lymphocyte activation
GO:0006955	6.58E-06	2.13889851	49	849	immune response
GO:0002694	8.39E-06	3.09994066	23	274	regulation of leukocyte activation
GO:0050851	9.62E-06	4.92119201	13	101	antigen receptor-mediated signaling pathway
GO:0050778	9.62E-06	2.98843257	24	296	positive regulation of immune response
GO:0002696	9.64E-06	3.66336418	18	183	positive regulation of leukocyte activation
GO:0046649	1.18E-05	2.6919509	28	382	lymphocyte activation
GO:0002429	1.64E-05	4.65455007	13	106	immune response-activating cell surface receptor signaling
GO:0050867	1.74E-05	3.49146793	18	191	positive regulation of cell activation
GO:0002682	2.19E-05	2.15226381	42	716	regulation of immune system process
GO:0050865	2.77E-05	2.85520964	23	295	regulation of cell activation
GO:0002768	3.27E-05	4.3260472	13	113	immune response-regulating cell surface receptor signaling
GO:0001775	3.96E-05	2.19937828	37	614	cell activation
GO:0051251	4.81E-05	3.49455338	16	169	positive regulation of lymphocyte activation
GO:0002684	6.68E-05	2.37838949	29	443	positive regulation of immune system process
GO:0001817	7.30E-05	2.66422252	23	314	regulation of cytokine production
GO:0045321	7.84E-05	2.35477062	29	447	leukocyte activation
GO:0042102	8.86E-05	5.60822928	9	62	positive regulation of T cell proliferation
GO:0072676	0.00012123	9.38067713	6	27	lymphocyte migration
GO:0050870	0.00016455	3.62921098	13	132	positive regulation of T cell activation
GO:0002757	0.0002046	3.0492517	16	191	immune response-activating signal transduction
GO:0032946	0.00026687	4.29254956	10	87	positive regulation of mononuclear cell proliferation
GO:0050671	0.00026687	4.29254956	10	87	positive regulation of lymphocyte proliferation
GO:0070665	0.00032171	4.18313717	10	89	positive regulation of leukocyte proliferation

GO:0002764	0.00034502	2.89777433	16	200	immune response-regulating signaling pathway
GO:0034097	0.00035577	2.23577376	26	418	response to cytokine stimulus
GO:0042129	0.00042137	4.02902582	10	92	regulation of T cell proliferation
GO:0001816	0.00042337	2.3338139	23	354	cytokine production
GO:0046634	0.00052576	5.61187699	7	48	regulation of alpha-beta T cell activation
GO:0032944	0.00052932	3.36879362	12	130	regulation of mononuclear cell proliferation
GO:0050670	0.00052932	3.36879362	12	130	regulation of lymphocyte proliferation
GO:0044764	0.00062582	1.9584516	33	604	multi-organism cellular process
GO:0070663	0.00069539	3.25718419	12	134	regulation of leukocyte proliferation
GO:0002819	0.00075638	4.06453932	9	82	regulation of adaptive immune response
GO:0019221	0.00082386	2.42144052	19	281	cytokine-mediated signaling pathway
GO:0072678	0.0008361	11.8808777	4	15	T cell migration
GO:0045580	0.00084407	4.46275128	8	67	regulation of T cell differentiation
GO:2000514	0.00110629	7.43974325	5	27	regulation of CD4-positive, alpha-beta T cell activation
GO:0050900	0.00112752	2.5705084	16	223	leukocyte migration
GO:0045619	0.00116141	3.80230993	9	87	regulation of lymphocyte differentiation
GO:0016032	0.00119196	1.89666667	32	602	viral reproduction
GO:0007040	0.00131408	7.11564967	5	28	lysosome organization
GO:0060760	0.00139019	10.0512821	4	17	positive regulation of response to cytokine stimulus
GO:0002443	0.00147812	2.81419785	13	166	leukocyte mediated immunity
GO:0005975	0.00161453	1.83936497	33	639	carbohydrate metabolic process
GO:0071345	0.00163577	2.17202772	21	344	cellular response to cytokine stimulus
GO:0001819	0.00164681	2.77739081	13	168	positive regulation of cytokine production
GO:0002821	0.00188253	5.17630058	6	44	positive regulation of adaptive immune response
GO:0002253	0.00194107	2.42704936	16	235	activation of immune response
GO:0042098	0.00195115	3.23328747	10	112	T cell proliferation
GO:0002449	0.00205863	3.00186617	11	132	lymphocyte mediated immunity
GO:0048584	0.00258072	1.64773442	44	952	positive regulation of response to stimulus
GO:0002703	0.00267963	3.65277778	8	80	regulation of leukocyte mediated immunity
GO:0016239	0.00274588	13.9672534	3	10	positive regulation of macroautophagy
GO:2000757	0.00274588	13.9672534	3	10	negative regulation of peptidyl-lysine acetylation

GO:0030217	0.00308762	2.8359375	11	139	T cell differentiation
GO:0007049	0.00316805	1.56780229	52	1185	cell cycle
GO:0032943	0.00317905	2.67877583	12	160	mononuclear cell proliferation
GO:0046651	0.00317905	2.67877583	12	160	lymphocyte proliferation
GO:0046637	0.00321161	5.64046507	5	34	regulation of alpha-beta T cell differentiation
GO:0042384	0.00321892	3.96106947	7	65	cilium assembly
GO:0050871	0.00328987	4.57238876	6	49	positive regulation of B cell activation
GO:0002706	0.00351032	3.8935888	7	66	regulation of lymphocyte mediated immunity
GO:0006368	0.00351032	3.8935888	7	66	transcription elongation from RNA polymerase II promoter
GO:0021549	0.0036461	4.46807672	6	50	cerebellum development
GO:0032663	0.00365639	5.45196926	5	35	regulation of interleukin-2 production
GO:0044723	0.00368764	1.89211535	25	467	single-organism carbohydrate metabolic process
GO:0002250	0.00408041	2.59008074	12	165	adaptive immune response
GO:0070661	0.00428318	2.57303285	12	166	leukocyte proliferation
GO:0006470	0.00434623	2.86448004	10	125	protein dephosphorylation
GO:0031295	0.0044434	4.27305856	6	52	T cell costimulation
GO:0045621	0.0044434	4.27305856	6	52	positive regulation of lymphocyte differentiation
GO:0001909	0.00467558	5.11032061	5	37	leukocyte mediated cytotoxicity
GO:0030098	0.00479546	2.42741196	13	190	lymphocyte differentiation
GO:0019722	0.00483483	3.28517442	8	88	calcium-mediated signaling
GO:0031294	0.00488703	4.18177346	6	53	lymphocyte costimulation
GO:0046635	0.00525428	4.95502576	5	38	positive regulation of alpha-beta T cell activation
GO:0006370	0.00526021	6.52931034	4	24	7-methylguanosine mRNA capping
GO:0051325	0.00535765	1.9802424	20	356	interphase
GO:0007033	0.00587069	4.01038103	6	55	vacuole organization
GO:0035710	0.00588187	4.80886591	5	39	CD4-positive, alpha-beta T cell activation
GO:0030902	0.00591806	3.1655926	8	91	hindbrain development
GO:0051320	0.00603974	2.7209898	10	131	S phase
GO:0032268	0.00606643	1.54038806	47	1082	regulation of cellular protein metabolic process
GO:0032729	0.00611647	6.21784346	4	25	positive regulation of interferon-gamma production
GO:0043370	0.00611647	6.21784346	4	25	regulation of CD4-positive, alpha-beta T cell differentiation

GO:0021680	0.00612093	9.77449857	3	13	cerebellar Purkinje cell layer development
GO:0046641	0.00612093	9.77449857	3	13	positive regulation of alpha-beta T cell proliferation
GO:0002705	0.00656044	4.67105805	5	40	positive regulation of leukocyte mediated immunity
GO:0002708	0.00656044	4.67105805	5	40	positive regulation of lymphocyte mediated immunity
GO:0032623	0.00656044	4.67105805	5	40	interleukin-2 production
GO:0046631	0.00661997	3.42626	7	74	alpha-beta T cell activation
GO:0050790	0.00673512	1.50592725	51	1202	regulation of catalytic activity
GO:0006184	0.00697485	2.10298564	16	268	GTP catabolic process
GO:0006488	0.00706058	5.93469175	4	26	dolichol-linked oligosaccharide biosynthetic process
GO:0009452	0.00706058	5.93469175	4	26	7-methylguanosine RNA capping
GO:0036260	0.00706058	5.93469175	4	26	RNA capping
GO:0042454	0.00736298	1.95336513	19	342	ribonucleoside catabolic process
GO:0019932	0.00742782	2.63298246	10	135	second-messenger-mediated signaling
GO:1901069	0.00747062	2.08605174	16	270	guanosine-containing compound catabolic process
GO:0022037	0.00760635	3.77801245	6	58	metencephalon development
GO:0006925	0.00761903	8.88512633	3	14	inflammatory cell apoptotic process
GO:0032106	0.00761903	8.88512633	3	14	positive regulation of response to extracellular stimulus
GO:0032109	0.00761903	8.88512633	3	14	positive regulation of response to nutrient levels
GO:0045061	0.00761903	8.88512633	3	14	thymic T cell selection
GO:0045577	0.00761903	8.88512633	3	14	regulation of B cell differentiation
GO:0051180	0.00761903	8.88512633	3	14	vitamin transport
GO:0051247	0.00788128	1.61565761	35	763	positive regulation of protein metabolic process
GO:0009225	0.00809611	5.67616192	4	27	nucleotide-sugar metabolic process
GO:0051208	0.00809611	5.67616192	4	27	sequestering of calcium ion
GO:0050792	0.00812155	2.98440803	8	96	regulation of viral reproduction
GO:0031399	0.00815573	1.57166715	39	875	regulation of protein modification process
GO:0045582	0.00892226	4.30115274	5	43	positive regulation of T cell differentiation
GO:2000756	0.00922645	5.43917625	4	28	regulation of peptidyl-lysine acetylation
GO:0008625	0.00931476	8.14398281	3	15	extrinsic apoptotic signaling pathway via death domain
GO:0031648	0.00931476	8.14398281	3	15	protein destabilization

GO:0045742	0.00931476	8.14398281	3	15	positive regulation of epidermal growth factor receptor signaling pathway
GO:0050856	0.00931476	8.14398281	3	15	regulation of T cell receptor signaling pathway
GO:0009164	0.00958193	1.89886573	19	351	nucleoside catabolic process
GO:0051329	0.00958193	1.89886573	19	351	interphase of mitotic cell cycle
