# Supplementary material for: Integrative Analysis of DNA Methylation and Gene Expression Data Identifies EPAS1 as a Key Regulator of COPD
Source: PLoS Genet. 2015 Jan 8;11(1):e1004898. doi: 10.1371/journal.pgen.1004898 (PMC4287352; doi:10.1371/journal.pgen.1004898)
Supplement: S18 Table — GO enrichment analysis of PAX9 downstream genes in COPD. (PDF) [file pgen.1004898.s027.pdf]

STable 18. GO enrichment analysis of *PAX9* downstream genes in COPD

GOBPID	Pvalue	OddsRatio	Count	Size	Term
GO:0003341	1.93E-10	36.1057382	10	16	cilium movement
GO:0001539	3.24E-10	18.6274619	12	26	ciliary or flagellar motility
GO:0042384	2.40E-09	7.74861111	17	65	cilium assembly
GO:0007017	6.12E-09	3.04377785	42	352	microtubule-based process
GO:0060271	1.09E-08	5.95005624	19	89	cilium morphogenesis
GO:0001578	1.22E-08	18.0431979	10	22	microtubule bundle formation
GO:0003351	3.78E-08	50.2698718	7	10	epithelial cilium movement
GO:0007018	1.03E-07	4.35447329	22	133	microtubule-based movement
GO:0030031	1.52E-07	3.79894216	25	170	cell projection assembly
GO:0010927	1.74E-06	4.0716381	19	121	cellular component assembly involved in morphogenesis
GO:0000226	0.00015646	2.47436138	24	236	microtubule cytoskeleton organization
GO:0060119	0.00018034	9.20647107	6	20	inner ear receptor cell development
GO:0007223	0.00068045	14.2766093	4	10	Wnt receptor signaling pathway, calcium modulating pathway
GO:0007283	0.00076482	2.08424735	27	310	spermatogenesis
GO:0048232	0.00080314	2.07671831	27	311	male gamete generation
GO:0051298	0.00129064	5.85447566	6	28	centrosome duplication
GO:0021591	0.00208002	9.51519014	4	13	ventricular system development
GO:0048839	0.00374654	2.52804361	13	124	inner ear development
GO:0030317	0.00538657	5.10171502	5	26	sperm motility
GO:0030030	0.00615971	1.51770423	49	758	cell projection organization
GO:0000086	0.00636277	2.35639081	13	132	G2/M transition of mitotic cell cycle
GO:0030520	0.00637136	4.86938349	5	27	intracellular estrogen receptor signaling pathway
GO:0060795	0.00637136	4.86938349	5	27	cell fate commitment involved in formation of primary germ layer
GO:0007098	0.00647113	4.02135317	6	38	centrosome cycle
GO:0060113	0.00647113	4.02135317	6	38	inner ear receptor cell differentiation
GO:0043583	0.00673472	2.25492421	14	148	ear development
GO:0007040	0.00747586	4.65725471	5	28	lysosome organization
GO:0030199	0.00747586	4.65725471	5	28	collagen fibril organization

GO:0006457	0.0087213	2.05721741	16	184	protein folding
