# Supplementary material for: Integrative Analysis of DNA Methylation and Gene Expression Data Identifies EPAS1 as a Key Regulator of COPD
Source: PLoS Genet. 2015 Jan 8;11(1):e1004898. doi: 10.1371/journal.pgen.1004898 (PMC4287352; doi:10.1371/journal.pgen.1004898)
Supplement: S19 Table — Motif enrichment analysis of downstream genes of key regulator EPAS1 in COPD. (PDF) [file pgen.1004898.s028.pdf]

STable 19. Motif enrichment analysis of downstream genes of key regulator *EPAS1* in COPD

MotifName	Size	Overlap	FET pvalue	Oddsratio
V$AREB6_03	258	24	0.00884701	1.775690244
CCTGTGA,MIR-513	125	14	0.00916934	2.175288131
V$AP1_C	275	25	0.01009532	1.731475406
AACTGGA,MIR-145	234	22	0.01055326	1.79489173
V$ZIC2_01	247	22	0.01876613	1.689519611
V$TAXCREB_01	137	14	0.01938402	1.961333867
V$PAX5_01	154	15	0.02350907	1.859803907
AGGCACT,MIR-515-3P	89	10	0.0248504	2.177064968
V$XBP1_01	133	13	0.0326059	1.864842999
GCAAGAC,MIR-431	45	6	0.03635799	2.640048514
V$HNF3ALPHA_Q6	208	18	0.03997613	1.632728304
MYAATNNNNNNNGGC_UNKNOWN	111	11	0.04308449	1.89143413
TATTATA,MIR-374	284	23	0.04312283	1.520510182
V$CEBPB_02	258	21	0.04930809	1.527644841
