# Supplementary material for: Integrative Analysis of DNA Methylation and Gene Expression Data Identifies EPAS1 as a Key Regulator of COPD
Source: PLoS Genet. 2015 Jan 8;11(1):e1004898. doi: 10.1371/journal.pgen.1004898 (PMC4287352; doi:10.1371/journal.pgen.1004898)
Supplement: S1 Text — Supplementary results and methods. (DOCX) [file pgen.1004898.s029.docx]

**Supplementary text for**

**Integrative analysis of DNA methylation and gene expression data identifies *EPAS1* as a key regulator of COPD**

Seungyeul Yoo^1,2^, Sachiko Takikawa^3^, Patrick Geraghty^4^, Carmen Argmann^1,2^, Joshua Campbell^5^, Luan Lin^1,2^, Tao Huang^1,2^, Zhidong Tu^1,2^, Robert Feronjy^4^, Avrum Spira^5^, Eric E. Schadt^1,2^, Charles A. Powell^3^, and Jun Zhu^1,2^*

^1^Institute of Genomics and Multiscale Biology, Mount Sinai School of Medicine, NY, 10029, USA;

^2^Department of Genetics and Genomic Sciences, Mount Sinai School of Medicine, NY, 10029, USA;

^3^Division of Pulmonary, Critical Care and Sleep Medicine, Mount Sinai School of Medicine, NY, 10029, USA;

^4^Department of Medicine, St. Luke’s Roosevelt Medical Center, Mount Sinai School of Medicine, NY, 10029, USA;

^5^Division of Computational Biomedicine, Department of Medicine, Boston University School of Medicine, 72 East Concord Street, Boston, MA 02118, USA

* All corresponds should be addressed to Dr. Jun Zhu (jun.zhu@mssm.edu)

**Supplementary Results**

Heterogeneous gene expression and methylation profiles in COPD and CTRL lung samples

Lung is a complex organ consisting of bronchioles and alveoli. To carry out its function, lung tissue is made up of more than 20 cell types including epithelial, connective tissue, blood vessels, nervous tissue, bone, muscle, and cartilage cells. COPD is a heterogeneous disease characterized by histopathologic features and respiratory symptoms. To explore whether molecular traits can be used to classify COPD and CTRL lung samples in an unsupervised fashion, we selected 1000 mRNA probes and 1000 methyl probes with the largest variances within each sample type and applied hierarchical clustering within the CTRL and COPD groups separately (shown in Supplementary Figure S9). Interestingly, the same set of genes classified samples into two distinct groups in both COPD (Supplementary Figure S9A) and CTRL (Supplementary Figure S9B). There were 250 genes in common as highlighted by the red boxes in Figures S9A and S9B, and these genes were enriched for cilium-related functions (Supplementary Table S15). While ciliary malfunctions in humans can cause several diseases including immotile-cilia syndrome or hydrocephalus [[1](#_ENREF_1),[2](#_ENREF_2)], none of the 250 genes in common were differentially expressed between CTRL and COPD. In addition, samples in the two subgroups of COPD samples classified by the ciliary related genes were not significantly different from each other with respect to disease phenotypes such as axial distribution of emphysema, coughing status, and FEV1 pre Bronchodilator predicted categorical (t-test p-values=0.51, 0.55, and 0.16, respectively). These results support that ciliary function is not directly associated with COPD pathophysiology, but rather important for lung tissue function in general.

In a similar cluster analysis using the top 1000 methyl probes with the largest variances, heterogeneity in methylation status was also observed. While the samples in COPD (Supplementary Figure S9C) showed less clear differences in methylation levels (as depicted in the red boxes in the figure) compared to those in the CTRL group (Supplementary Figure S9D), a common set of methylation loci comprised of 94 common genes, classified the samples into two subgroups in both the CTRL and COPD groups. While these 94 genes included 5 genes related to lung development (p-value=0.001): *CTNNB1*, *FGRR2*, *FOXA2*, *LAMA5*, and *SIM2*, the methylation levels of these genes were also not associated with disease status and severity. This suggests that the large heterogeneity in methylation levels of COPD is not directly related to the disease.

Overall, these results show that there are heterogeneities among molecular traits within COPD but these heterogeneities are not consistent with each other. Indeed, only 35 genes were in common between the distinguishing mRNA and methyl probes (Fisher’s exact p-value=1). In addition, the individual samples as clustered by mRNA probes were not similarly grouped by the methyl probes in either the CTRL or COPD groups. These data suggest that studying methylation and gene expression separately would yield only a limited understanding of epigenetic regulation driven by methylation. Thus, it is necessary to integrate both molecular traits together to achieve a clearer understanding of the mechanism.

Key regulators of methylation regulation in normal lung

To understand the consequences of normal epigenetic control of the lung, we examined the influences of methylation variation of the key regulators identified in the CTRL group. Since there are many key regulators, it is difficult to analyze them all in a biological context at once. Therefore, to put 67 key regulators into relevant biological context, we clustered the key regulators according to their methylation variations and their downstream target genes. The methylation levels of CTRL key regulators were highly correlated to each other, and more than half of the key regulators could be grouped into two clusters (Supplementary Figure S3A). We also compared downstream genes of these key regulators using topological overlap [[3](#_ENREF_3)]$O_{T}(i,j)=\frac{\left|D_{i}\cap D_{j}\right|}{\min(\left|D_{i}\right|,\left|D_{j}\right|)}$ where $D_{i}$ is the downstream gene set of key regulator $i$ and use the topological overlap as the similarity measurement in hierarchical clustering analysis. As expected, key regulators in the same clusters in Supplementary Figure S3A regulate similar sets of downstream genes (Supplementary Figure S3B). *WDR90*, a gene of unknown function, included in the second cluster C2 is the top key regulator, regulating 318 downstream genes. Its downstream genes are enriched for the function of cilium and microtubule related functions (p-value=1.18×10^-18^), which in the lung functions to keep the airways clear of mucus and dirt, allowing one to breathe easily and without irritation. Among the *WDR90* downstream genes, 204 genes overlap with the 339 cilium-related genes identified in the cluster of CTRL samples in Supplementary Figure S9B (Fisher’s exact test p-value=1.7×10^-293^). Half of the top 10 key regulators in CTRL share a common set of downstream genes with *WDR90* and, therefore, their downstream genes are significantly overlapped with the cilium-related genes, too. This indicates that the variation of cilium-related genes is regulated by methylation level of promoter regions of *WDR90* and other key regulators in normal lung. Other key regulators controlled completely different sets of genes (Supplementary Figure S3B), and downstream genes of these key regulators did not overlap with the cilium-related gene sets. Key regulators in the largest cluster in Supplementary Figure S3A share common downstream genes enriched for metabolic processes, RNA processing, RNA splicing, and histone modification. There are also a group of key regulators not included in any clusters but controlling relatively unique downstream genes. *COL12A1* downstream genes are enriched for organelle localization (p-value=0.0006), intracellular protein kinase cascade (p-value=0.0012), and system developments (p-value=0.0056). *FOXK2* regulates downstream genes enriched for cell cycle (p-value=0.0007) while *MAP4K4* downstream genes are responsible for cytoskeleton organization (p-value =8.3x10^-5^), regulation of cell adhesion (p-value=0.0002), and other cellular processes. In summary, these results highlight how DNA methylation in lung is associated with the most transcriptionally varied genes in non-disease samples suggesting a significant role for methylation control in normal lung function. Importantly, we reveal that multiple key regulators target similar sets of genes indicating that the epigenetic control by methylation in the normal lung is seemingly complex and not mediated by a single master gene.

Key regulators of methylation regulation in diseased COPD lung

With an understanding of epigenetic control by methylation in normal lung, we next sought to investigate the epigenetic control underlying diseased lung. Similar to the CTRL setting, 126 key regulators identified in COPD could be clustered according to their methylation level (Supplementary Figure S4A) or by the overlap of their downstream genes (Supplementary Figure S4B). Clusters are not well separated based on methylation variation (Supplementary Figure S4A), there are clearly three clusters as shown in Supplementary Figure S4B. The largest cluster, C1 in Supplementary Figure S4B, consisted of 67 regulators including *GAK* which regulates the largest number of downstream genes, 1,817 genes. *GAK* downstream genes are enriched for RNA splicing (p-value=6.02×10^-6^), mRNA metabolic process (p-value=2.05×10^-5^), chromatin modification (p-value=2.48×10^-5^), and several other functions (Supplementary Table S16). Other regulators in the same cluster share similar downstream genes (about 40-70% overlaps) and, therefore, share similar GO categories. The second cluster, C2, includes 47 regulators. *ETF1*, one of regulators in the C2, regulates downstream genes enriched for defense processes including immune system response (p-value=2.43×10^-6^), regulation of T cell activation (p-value=2.65×10^-6^), and regulation of immune response (p-value=2.81×10^-6^) (Supplementary Table S17). Some regulators in C2 share downstream genes with the regulators in the C1. For example, *ABHD14B* shares 45% of downstream genes with *GAK* (Fisher’ exact test p-value=5.5×10^-131^) and 30% with *ETF1* in C2 (Fisher’s exact test p-value=4.4×10^-20^), which suggests that immune response and metabolic process are co-regulated [[4](#_ENREF_4),[5](#_ENREF_5)]. The third cluster, C3, includes only 6 regulators; *PPIL6, PAX9, CCNA1, DNAH3, EZR,* and *RBM8A*. Downstream genes of these regulators are significantly overlapped with ciliary related genes (Fisher’s exact test p-value - *PPIL6*: 2.6×10^-270^, *PAX9*: 1.5×10^-212^, *CCNA1*: 1.3×10^-241^, *DNAH3*: 5.8×10^-237^, *EZR*: 7.9×10^-115^, and *RBM8A*: 4.4×10^-169^). GO terms enriched in *PAX9* downstream genes are listed in the Supplementary Table S18. *PAX9* itself is included in *WDR90* downstream genes in CTRL so it is consistent that ciliary related genes are regulated by methylation in both CTRL and COPD.

The above results suggest that there are three groups of key regulators as defined by their downstream target genes and that the overall epigenetic mechanism in COPD may be driven globally by multiple regulators rather than by a single master regulator. While several metabolic processes are co-regulated by regulators in C1 and C2, significant enrichment of immune response is observed mostly in C2 regulators. Ciliary function is controlled similarly as in CTRL. While key regulators are different from each other, epigenetic regulations involved with metabolic process and ciliary function are consistent between CTRL and COPD.

*EPAS1* downstream genes share common regulatory motifs in promoters and 3’ untranslated regions

To further understand mechanisms of how *EPAS1* regulates downstream genes, we tested if its downstream genes are enriched of regulatory motifs. Molecular Signature Databases (MSigDB) C3 gene sets consist of a list of transcription factors, microRNA and their target genes [[6](#_ENREF_6)]. The *EPAS1* downstream genes are significantly overlapped with 15 motif sets (p-value<0.05) (Supplementary Table S19). The binding motif of *AREB6*, which plays a key role in the repression of *IL2* gene expression [[7](#_ENREF_7)], is the most significantly enriched in *EPAS1* downstream genes. *AREB6*, also known as *ZEB1*, is also one of several major epithelial-mesenchymal transition (EMT) regulatory genes, an important observation, as EMT has been implicated in the development of COPD as well as lung cancer [[8](#_ENREF_8)] .There are also 6 miRNA binding motifs enriched in *EPAS1* downstream genes. The common functions of genes within these sets are signal transduction, multicellular organismal development, and metabolic processes, and system developments. These results suggest that in addition to directly binding to HIF response elements, *EPAS1* regulates downstream gene expression by regulating or interacting with other transcription factors or miRNAs.

**Supplementary Methods**

Processing methylation data

We used the bioconductor package “charm” developed for processing DNA methylation data generated using Nimblegen microarrays and McrBC protocol. Charm version 2.8 was used. Probe annotation information was extracted from the NimbleGen platform design file “pd.feinberg_hg18_me_hx1”. After loading them, we followed the vignettes provided by authors to read and convert rawdata into beta values. The two key commands are “readCharm” to read two channel intensities of array data and “methp” to estimate DNA methylation from CHARM microarray in terms of percentages. “methp” also performs normalization within (loess normalization) and between (quantile normalization) samples in the command. Commands below were used to generate beta values.

setwd("/scratch/yoos01/Lung_Dataset/Raw/CHARM/Gender_Check/Read_methylation_all")

library("preprocessCore")

library("charm")

library("pd.feinberg.hg18.me.hx1")

library("BSgenome.Hsapiens.UCSC.hg18")

library("gplots")

library("GenomicFeatures")

library("GenomicRanges")

library("marray")

### Read list of samples

pd<-read.table("CTRL_fil_samples.txt",header=TRUE,sep="\t")

res<-validatePd(pd)

pd<-pd[!(pd$tissue=="None"),]

### Read raw methylation intenstiy

rawData<-readCharm(pd$filename,path="/projects/zhuj05a/Lung_Dataset/LGRC/Raw/Charm/3_CTRL",sampleKey=pd)

dim(rawData)

### Perform quality control based on pmsignal and pmquality

qual<-qcReport(rawData,file="CTRL_qcReport.pdf")

rawData=rawData[,qual$pmSignal>=78]

dim(rawData)

qual=qual[qual$pmSignal>=78,]

pd=pd[pd$sampleID%in%rownames(qual),]

pData(rawData)$qual=qual$pmSignal

chr<-pmChr(rawData)

pos<-pmPosition(rawData)

pns<-probeNames(rawData)

seq<-pmSequence(rawData)

pmq<-pmQuality(rawData)

rmpmq<-rowMeans(pmq)

length(rmpmq)

okqc<-which(rmpmq>75)

length(okqc)

### Get control index and remove control probes

ctrlind<-getControlIndex(rawData,subject=Hsapiens)

length(ctrlind)

cqc<-controlQC(rawData=rawData,controlIndex=ctrlind,IDcol="sampleID",expcol="tissue",ylimits=c(-6,8),outfile="CTRL_contrlQC.pdf",height=7,width=9)

### Get the beta value using methp

grp<-pData(rawData)$tissue

p<-methp(rawData,controlIndex=ctrlind,plotDensity="CTRL_dentity.pdf",plotDensityGroups=grp)

dim(p)

rownames(p)<-paste(chr,pos,sep=":")

colnames(p)<-unique(pd$sampleID)

### Sort probes based on their chromosomal locations. Remove probes from X and Y chromosome.

index<-setdiff(okqc,ctrlind)

index<-index[order(chr[index],pos[index])]

p<-p[index,]

dim(p)

seq<-seq[index]

chr<-chr[index]

pos<-pos[index]

pns<-pns[index]

### If multiple probes are observed average the intensity

p<-avereps(p,rownames(p))

dim(p)

colnames(p)<-sapply(strsplit(colnames(p),"_"),"[",1)

rnames<-rownames(p)

chr<-sapply(strsplit(rnames,":"),"[",1)

### Get the intensity for the sex chromosomes

q<-p[(chr=="chrX" | chr=="chrY"),]

write.table(q,file="CTRL_methyp_sex_chromosome.txt",row.names=TRUE,quote=FALSE,col.names=TRUE,sep="\t")

p<-p[!(chr=="chrX" | chr=="chrY"),]

dim(p)

### Save beta value of all probes

write.table(p,file="CTRL_methp_all.txt",row.names=TRUE,quote=FALSE,col.names=TRUE,sep="\t")

The causality test for inferring putative causal genes to COPD disease

Similar to infer causal/reactive relationships among gene expression traits using the model $m_{j}\to g_{j}\to g_{i}$ , we can test whether a gene’s methylation and expression levels are causal to the COPD disease status (COPD or non-COPD) using the causality test model $m_{j}\to g_{j}\to DiseaseStatus$. At t-test p-value <0.01, there were 1,594 differentially expressed genes (FDR=0.09) and 6,416 genes differentially methylated in CpG islands (FDR=0.04), and 704 genes were both differentially expressed and differentially methylated. To test whether a gene’s methylation level is independent of the COPD disease status conditioned on the gene’s expression level, we tested association between $m_{j}|g_{j}$ and the disease status. At the t-test p-value >0.01, 639 of 704 differentially expressed and methylated genes were causal to the COPD disease status, the corresponding FDR was 0.047 based on permutation tests. *EPAS1* was one of 639 inferred putative COPD causal genes.

**References**

1. Afzelius BA (2004) Cilia-related diseases. J Pathol 204: 470-477.

2. Pan J, Wang Q, Snell WJ (2005) Cilium-generated signaling and cilia-related disorders. Lab Invest 85: 452-463.

3. Ravasz E, Somera AL, Mongru DA, Oltvai ZN, Barabasi AL (2002) Hierarchical organization of modularity in metabolic networks. Science 297: 1551-1555.

4. McCarthy SA, Mufson RA, Pearce EJ, Rathmell JC, Howcroft TK (2013) Metabolic reprogramming of the immune response in the tumor microenvironment. Cancer Biol Ther 14: 315-318.

5. Lartigue L, Faustin B (2013) MITOCHONDRIA: Metabolic regulators of innate immune responses to pathogens and cell stress. Int J Biochem Cell Biol.

6. Xie X, Lu J, Kulbokas EJ, Golub TR, Mootha V, et al. (2005) Systematic discovery of regulatory motifs in human promoters and 3' UTRs by comparison of several mammals. Nature 434: 338-345.

7. Becker JC, Brabletz T, Kirchner T, Conrad CT, Brocker EB, et al. (1995) Negative transcriptional regulation in anergic T cells. Proc Natl Acad Sci U S A 92: 2375-2378.

8. Bartis D, Mise N, Mahida RY, Eickelberg O, Thickett DR (2013) Epithelial-mesenchymal transition in lung development and disease: does it exist and is it important? Thorax.
